# Effectiveness of Pharmacological Intervention Among Men with Infertility: A Systematic Review and Network Meta-Analysis

**DOI:** 10.3389/fphar.2021.638628

**Published:** 2021-08-16

**Authors:** Muhammad Nabeel Shahid, Tahir Mehmood Khan, Chin Fen Neoh, Qi Ying Lean, Allah Bukhsh, Mahmathi Karuppannan

**Affiliations:** ^1^ Department of Pharmacy Practice, Faculty of Pharmacy, Universiti Teknologi MARA (UiTM), Bandar Puncak Alam, Malaysia; ^2^ Department of Pharmacy Practice, Institute of Pharmaceutical Sciences, University of Veterinary and Animal Sciences, Lahore, Pakistan; ^3^ School of Pharmacy, Monash University, Subang Jaya, Malaysia; ^4^ Faculty of Pharmacy, Universiti Teknologi MARA (UiTM), Pulau Pinang, Malaysia; ^5^ Vector-Borne Diseases Research Group (VERDI), Pharmaceutical and Life Sciences CoRe, Universiti Teknologi MARA (UiTM), Shah Alam, Malaysia

**Keywords:** male infertility, meta-analysis, network meta-analysis, coenzyme Q10, follicle stimulating hormone, selective estrogen receptor modulator

## Abstract

***Background*.** Infertility is an emerging health issue for men. Comparative efficacy of different pharmacological interventions on male infertility is not clear. The aim of this review is to investigate the efficacy of various pharmacological interventions among men with idiopathic male infertility. All randomized control trials evaluating the effectuality of interventions on male infertility were included for network meta-analysis (NMA) from inception to 31 April 2020, systematically performed using STATA through the random effect model. The protocol was registered at PROSPERO (CRD42020152891).

***Results.*** The outcomes of interest were semen and hormonal parameters. Treatment effects (*p* < 0.05) were estimated through WMD at the confidence interval of 95%. Upon applying exclusion criteria, n=28 RCTs were found eligible for NMA. Results from NMA indicated that consumption of supplements increases sperm concentration levels [6.26, 95% CI 3.32, 9.21] in comparison to SERMs [4.97, 95% CI 1.61, 8.32], hormones [4.14, 95% CI 1.83, 6.46], and vitamins [0.15, 95% CI −20.86, 21.15)] with placebo, whereas the use of SERMs increased percentage sperm motility [6.69, 95% CI 2.38, 10.99] in comparison to supplements [6.46, 95% CI 2.57, 10.06], hormones [3.47, 95% CI 0.40, 6.54], and vitamins [−1.24, 95% CI −11.84, 9.43] with placebo. Consumption of hormones increased the sperm morphology [3.71, 95% CI, 1.34, 6.07] in contrast to supplements [2.22, 95% CI 0.12, 4.55], SERMs [2.21, 95% CI −0.78, 5.20], and vitamins [0.51, 95% CI −3.60, 4.62] with placebo. Supplements boosted the total testosterone levels [2.70, 95% CI 1.34, 4.07] in comparison to SERMs [1.83, 95% CI 1.16, 2.50], hormones [0.40, 95% CI −0.49, 1.29], and vitamins [−0.70, 95% CI −6.71, 5.31] with placebo. SERMs increase the serum FSH levels [3.63, 95% CI 1.48, 5.79] better than hormones [1.29, 95% CI −0.79, 3.36], vitamins [0.03, 95% CI −2.69, 2.76], and supplements [−4.45, 95% CI −7.15, −1.76] in comparison with placebo.

***Conclusion.*** This review establishes that all interventions had a significantly positive effect on male infertility. Statistically significant increased sperm parameters were noted in combinations of zinc sulfate (220 mg BID), clomiphene citrate (50 mg BID), and testosterone undecanoate and CoQ10; tamoxifen citrate and FSH were shown to improve the hormonal profile in infertile males.

**Systematic Review Registration**: PROSPERO, identifier [CRD42020152891].

## Introduction

Numerous studies have been published reporting male infertility and a drop in sperm quality (concentration, motility, and morphology) or dismissing the same (Carlsen et al., 1992; Auger et al., 1995; Fisch et al., 1996; Marimuthu et al., 2003; Lackner et al., 2005; Kumar and Singh, 2015). Retrospective data analysis indicates that overall sperm parameters have declined in some parts of the world with geographical variations in semen quality (Auger and Jouannet, 1997; Jørgensen et al., 2001; Swan, 2006; Kumar and Singh, 2015). These geographical variations in semen characteristics could be due to environmental, nutritional, socioeconomic, or other unknown causes (Fisch and Goluboff, 1996) and this coincides with ever-increasing male genital tract abnormalities including testicular cancer and cryptorchidism in various countries (Giwercman and Skakkebaek, 1992; Bussen et al., 2004; Kumar and Singh, 2015).

Inability to achieve clinical conception despite 1 year of unprotected intercourse with the same partner is categorized as human infertility by the World Health Organization (WHO) (Zhao et al., 2016; Buhling et al., 2019; Cao et al., 2019; Hosseini et al., 2019; Wang et al., 2020). A total of 15% (48.5 million) couples worldwide are affected by any kind of infertility, and male infertility dominates the trend with 70% of all cases. Higher male infertility rates are seen in the population of Central Europe, Eastern Europe, and Africa (Zhao et al., 2016; Buhling et al., 2019; Cao et al., 2019; Hosseini et al., 2019; Wang et al., 2020).

The etiology of male factor infertility is multifactorial, primarily based on reduced and poor sperm quality. Concentration, morphology, and motility are three primary endpoints to assess the sperm quality (Buhling et al., 2019). Other factors include genetics (chromosomal aberrations, gene mutations, congenital anomalies, and polymorphisms), morphology of the reproductive system (testicular cancer, aplasia of the germinal cells, alteration in reproductive hormone, varicocele, defects in the transport of sperm, and bilateral sperm ducts), addictive disorders (alcoholism, smoking, and drug addiction), environmental factors (cigarette, smoking, exposure to certain chemicals, and nutritional deficiency), and alteration in spermatogenesis due to pathophysiological conditions (infectious diseases, pregnancy-related infections, urogenital infections, genitourinary dysplasia, immune system-related factor, abnormal levels of biochemical components of seminal plasma, and presence of antiserum antibodies (ASAs)) (Zhao et al., 2016; Buhling et al., 2019; Cao et al., 2019; Hosseini et al., 2019; Wang et al., 2020). These factors can lead to numerous abnormalities of the reproductive system and capacity for fertilization. Moreover, infertility can have severe damaging impacts on social, psychological, and economic well-being and health of the couples (Zhao et al., 2016; Buhling et al., 2019; Cao et al., 2019; Hosseini et al., 2019; Wang et al., 2020). Hormones, selective estrogen receptor modulator, antioxidants, vitamins, and enzymes are some of the treatment protocols for male infertility these days. Although several micronutrient supplementation products are available in the market that promises to improve the spermiogram, there are only a few existing evidence based data that address male factor infertility and micronutrient treatment options (Hamada et al., 2012; Cannarella et al., 2019; Cardoso et al., 2019; Hosseini et al., 2019; Wang et al., 2020).

To date, many systematic reviews and meta-analyses revealed the impact of different interventions in male infertility (Kamischke and Nieschlag, 1999; Boivin, 2003; Lafuente et al., 2013; Giahi et al., 2016; Hosseini et al., 2019; Smits et al., 2019; Wang et al., 2019; Garolla et al., 2020). Though systematic reviews and pairwise meta-analysis are vital tools for decision makers for contriving guidelines and clinical protocols, they produce partial information, because amongst several accessible interventions merely few are examined in head-to-head comparisons (Greco et al., 2015; Tonin et al., 2017). To overcome these issues, WHO introduced network meta-analysis (NMA). One of the several advantages of NMA is its ability to compare interventions quantitatively which otherwise are not directly comparable (Greco et al., 2015). This NMA will facilitate clinicians to mold interventions according to their choice to achieve desired therapeutic outcomes in male infertility, with maximum utilization of resources available.

This study compares effectiveness of multiple pharmacological groups including hormones (follicle stimulating hormone, testosterone undecanoate), selective estrogen receptor modulators (SERMs) (tamoxifen citrate and clomiphene citrate), supplements (coenzyme Q10, carnitine, L-Carnitine, zinc sulfate, fish oil, and Profertil), vitamins (vitamins A, D, and E and folic acid), and enzymes (kallikrein) alone and in combination through NMA. We choose to use sperm concentration, sperm motility, and sperm morphology as primary outcomes for male infertility related complications (Kumar and Singh, 2015; Buhling et al., 2019). Other secondary outcomes include serum total testosterone and serum follicle stimulating hormones (FSH).

## Methodology

Seven electronic databases (PubMed, Scopus, Cochrane library, Embase, EBSCOhost, Ovid, and Google scholar) were searched for data sources and strategies from inception till 31 April 2020. Medical subject headings [MeSH] and text terms were included for search terms in this review. The strategic search terms were as follows: “infertility” or “subfertility” or “subfertile” and “azoospermia” or “oligospermia” or “oligozoospermia” or “oligoasthenoteratozoospermia” or “genital disease” or “genitalia” or “genital” or “low sperm count” or “semen.” Details of search strategies used for each database are provided in the Supplementary Material.

## Inclusion and Exclusion Criteria

The inclusion criteria were set according to the PICOT framework (population, intervention, comparison, outcomes, and time), as follows (Table 1). Studies which were included comprise of the following:

1) Randomized control trials or cluster-randomized controlled trials.
2) Different interventions evaluating the efficacy of men infertility issues.
3) Those directed at male patients (≥18 years) with infertility only.
4) Reporting sperm concentration, sperm motility, and sperm morphology as primary clinical outcome (alone or in combination with any of the other clinical outcomes, such as serum total testosterone and serum FSH).
5) Those performed in inpatient, outpatient primary care or hospital settings.
6) Original study published in a peer-reviewed journal.
7) Article in English language.

**TABLE 1:** PICOT table of included studies.

Category	Description
Population	Infertile males
Intervention	Any pharmaceutical interventions approved according to country guidelines and mentioned in the articles eligible for inclusion in the study
Control	Any comparator/placebo in the articles eligible for inclusion
Outcome	Sperm concentration, sperm motility, sperm morphology, total serum FSH, and total serum testosterone
Time	Inception to April 2020

Additional details are mentioned in the Supplementary Material (Annexure 1).

Studies conducted using the observational study designs mentioned were excluded:

1) Observational studies.
2) Cohort studies.
3) Cross-sectional studies.
4) Nonrandomized control trials.
5) Expert opinions.
6) Case reports/series.
7) Editorials
8) Abstracts from conferences.
9) Review articles.
10) Studies involving animals.

## Study Selection

Two reviewers MNS and TMK screened titles and abstracts extracted from various databases using the well-defined selection criteria. Appropriate articles were then screened individually by the reviewers to access their inclusion eligibility. Resolution of disagreement was primarily through discussion.

## Data Extraction and Synthesis

MNS extracted the data from the studies included using standardized procedure. TMK independently reviewed the data for proper extraction. Details about title, publication year, authors, design of the study, country and study settings, sample size, age of the patients, gender of the patients, follow-up duration, interventions given, criteria for study inclusion and exclusion, and outcome of the study were extracted from each included study. In this review, changes from baseline to end of the intervention were summarized for both intervention and control groups as a result for the outcome measures.

## Risk of Bias Assessment

MNS and TMK evaluated the risk of bias (ROB) of the included studies using the Cochrane ROB tool. For RCTs, each ROB item was ranked as “low risk” if it was suspected that a bias would seriously alter the result; it would be “unclear” if it was expected that a bias would raise some uncertainty about the results; or it would be “high risk” if it was prospective that a bias would completely alter the result. Discussion was used to resolve disagreements among reviewers.

## Data Analysis

Meta-analysis (MA) and NMA were executed by using Review Manager 5.3 and STATA 14. Comparative efficacies for all interventions were estimated through calculation of mean difference using the random effect model. The assessed significance of results *p*-values were set to be < 0.05 with 95% confidence interval (CI).

Subgroup analyses were executed for primary and secondary clinical outcomes for different interventions to clarify the heterogeneity among the studies. Robustness of the study results was analyzed through subgroup analysis of the baseline values for sperm concentration, motility and morphology levels, intervention duration, influence of studies on primary clinical outcomes, and geographical areas of the performed studies. Moreover, forest plot was generated to study pairwise comparison for the study of treatment effect in NMA. In addition, treatment effect and mean difference were used to generate league tables to evaluate all the effects (direct and indirect) among interventions.

## Study Protocol Registration

The protocol was registered at PROSPERO (CRD42020152891).

## Results

**FIGURE 1:**
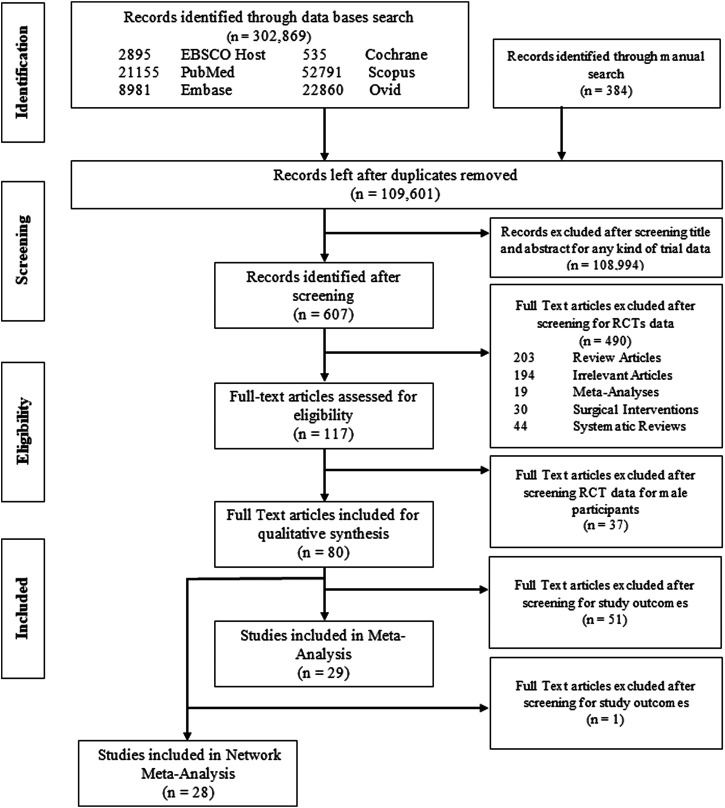
Prisma flow chart.

In total, 302,869 articles were extracted from the electronic database searches; after confiscating duplications (n = 193,268), the final count was reduced to 109,601. Evaluation of title and abstract excluded 108,994 studies not fulfilling inclusion criteria. 607 studies remained and full-text assessment was done from which 80 studies (n = 9529 participants) were finally included for qualitative synthesis, whereas quantitative analysis (MA) was conducted for 29 studies, details of which are presented in PRISMA flow diagram (Figure 1). Reasons for omission after full-text assessment are presented in Supplementary Table 1.

Among all 80 selective articles, 57 RCTs were blinded while the remaining 23 were not blinded. Among the studies included in this review, 13 were conducted in Iran (Safarinejad, 2009; Safarinejad and Safarinejad, 2009; Moradi et al., 2010; Safarinejad, 2011a; Safarinejad, 2011b; Nadjarzadeh et al., 2011; Safarinejad et al., 2011; Safarinejad et al., 2012; Mehni et al., 2014; Nadjarzadeh et al., 2014; Haghighian et al., 2015; Hosseini et al., 2016; Modarresi et al., 2019), 13 in Italy (Izzo et al., 1984; Foresta et al., 2002; Caroppo et al., 2003; Lenzi et al., 2003; Lenzi et al., 2004; Balercia et al., 2005; Foresta et al., 2005; Paradisi et al., 2006; Selice et al., 2011; Colacurci et al., 2012; Paradisi et al., 2014; Farrag et al., 2015; Maretti and Cavallini, 2017), 6 in the United States of America (Haas and Manganiello, 1987; Clark and Sherins, 1989; Gerris et al., 1991; Sigman et al., 2006; Wiehle et al., 2014; Helo et al., 2015), 6 in England (Willis et al., 1977; Pryor et al., 1978; Inton et al., 1979; Badenoch et al., 1988; Scott et al., 1998; Williams et al., 2020), and 4 from Austria (Pusch, 1988; Maier and Hienert, 1990; Imhof et al., 2012; Lipovac et al., 2016) and Germany (Knuth et al., 1987; Krause et al., 1992; Keck et al., 1994; Kamischke et al., 1998) while 3 were from India (Kumar et al., 2011; Ambiye et al., 2013; Gopinath et al., 2013), Iraq (Hadwan et al., 2012; Haje and Naoom, 2015; Alsalman et al., 2018), Japan (Yamamoto et al., 1995a; Yamamoto et al., 1995b; Matsumiya et al., 1998), and Greece (Adamopoulos et al., 1997; Adamopoulos et al., 2003; Farmakiotis et al., 2007). Two were from Egypt (Ghanem et al., 2010; ElSheikh et al., 2015), Denmark (Fedder et al., 2014; Jensen et al., 2020), China (Ding et al., 2015; Guo et al., 2015), and Switzerland (Comhaire et al., 1986; Crottaz et al., 1992) and n = 1 from Turkey (Çakan et al., 2009), Australia (Baker et al., 1984), Malaysia (Ismail et al., 2014), Nigeria (Mbah et al., 2012), France (Almeida et al., 1985), Saudi Arabia (Suleiman et al., 1996), South Africa (Wong et al., 2002), Finland (Park et al., 2016), Yugoslavia (Mićić and Dotlić, 1985), Canada (AinMelk et al., 1987), Israel (Glezerman et al., 1993), Mexico (Merino et al., 1997), and Scotland (Hargreave et al., 1984). Moreover, one multiple center study was conducted in Australia, Germany, Italy, Poland, Spain, and United Kingdom (Nieschlag et al., 2017).

## Characteristics of Participants

Eighty studies (n = 9529 patients) fulfilled the selection criteria of this review. The participants claimed infertility issue (WHO criteria) for at least 12 months or tested to have sperm count less than 20 million per ml. Sample size of the included studies ranged from n = 9 to n = 1,679; details are presented in Table 2

**TABLE 2:** Characteristics of studies treating male patients with infertility (n = 80).

No.	Author year	Study design	Country of study	Mean age of patients (year-old)	Type of male infertility	Patients (n)								Daily dosage regimen	Duration of intervention	Effects on infertility
						Total males in the trial	Intervention				Placebo	Control	Drop out			
							1	2	3	4						
1	Jensen et al. (2020)	Open label RCT	Denmark	19	Idiopathic infertility	1,679	98	210	25	75	—	1,125	146	Intervention 1: fish oil supplement	3 months	Improved
														Intervention 2: multivitamins		
														Intervention 3: vitamin C		
														Intervention 4: vitamin D		
2	Williams et al. (2020)	Double-blind RCT	England	23.3 ± 2.89	Idiopathic infertility	56	28	—	—	—	28	—	4	7 mg lycopene BD	12 weeks	Improved
3	Alsalman et al. (2018)	Open label RCT	Iraq	NA	Asthenospermia	120	60	—	—	—	—	60	-	Zinc sulfate(Cap) 220 mg OD	3 months	Improved
4	Modarresi et al. (2019)	Double-blind RCT	Iran	30	Idiopathic infertility	40	20	—	—	—	—	20	—	Spirulina 2 g OD + conventional regimen (220 mg/day zinc sulfate, 500 mg/day L-Carnitine, and 50 mg/day clomiphene)	12 weeks	Improved
5	Maretti and Cavallini, (2017)	Open label RCT	Italy	37	Idiopathic oligoasthenoteratozoospermia	46	20	—	—	—	—	21	5	Probiotic sachet OD	6 months	Improved
6	Nieschlag et al. (2017)	Open label RCT	Australia, Germany, Italy, Poland, Spain, and England	34	Hypogonadotropic hypogonadism	23	18	—	—	—	—	—	5	Corifollitropin alfa (every 2 weeks) + 1500 UI hCG (every week)	52 weeks	Improved
7	Jalal Husseini et al., 2016	Double-blind RCT	Iran	33.27 ± 5.38	Idiopathic infertility	106	50	—	—	—	50	—	6	Ginger powder 250 mg BD	3 months	Improved
8	Lipovac et al. (2016)	Open label RCT	Austria	40	Idiopathic infertility	299	156	143	—	—	—	—	—	Intervention 1:L-Carnitine 500 mg BD intervention 2: Profertil OD	3 months	Improved
9	Park et al. (2016)	Double-blind RCT	Finland	34	Oligospermia	80	19	20	18	—	20	—	3	Cap. Korean Red Ginseng 500 mg TID	12 weeks	Improved
10	Farrag et al. (2015)	Open label RCT	Italy	36.9 ± 5.1	Idiopathic infertility	82	36	—	—	—	—	46	—	150 IU rh-FSH/3 times a weeks	3 months	Improved
11	Helo et al. (2015)	Double-blind RCT	The United States	34	Hypogonadism	26	13	13	—	—	—	—	—	Intervention 1: 25 mg Clomiphene citrate Intervention 2: 1 mg Anastrozole	12 weeks	No significant benefit
12	Krause et al. (1992)	Open label RCT	Germany	30	Idiopathic oligozoospermia	76	39	—	—	—	37	—	—	Tamoxifen 30 mg OD	3 months	No significant benefit
13	Ding et al. (2015)	Double-blind RCT	China	35.5 ± 4.1	Idiopathic oligozoospermia	354	36	38	41	40	30	—	11	Intervention 1: 50 IU rhFSH	3 months	Improved
														Intervention 2: 100 IU rhFSH		
														Intervention 3: 200 IU rhFSH		
														Intervention 4: 300 IU rhFSH		
14	ElSheikh et al. (2015)	Single-blind RCT	Egypt	27.27 ± 4.83	Idiopathic oligoasthenozoospermia	9	30	30	30	—	—	—	—	Intervention 1: Vitamin E 400 mg OD Intervention 2: Clomiphene citrate 25 mg OD Intervention 3: Vitamin E 400 mg OD + Clomiphene citrate 25 mg OD	6 months	Improved
15	Guo et al. (2015)	Open label RCT	China	—	Idiopathic oligoasthenospermia	120	41	55	—	—	—	—	24	Intervention 1: Indomethacin 25 mg OD Intervention2: Tamoxifen 10 mg BD	3 months	Improved
16	Haghighain et al., 2015	Triple-blind RCT	Iran	32.98 ± 5.35	Infertile males	48	23	—	—	—	21	—	4	Alpha-lipoic acid 600 mg OD	12 weeks	Improved
17	Haje and Naoom, (2015)	Single-blindRCT	Iraq	37.54 ± 2.46	Idiopathic infertility	128	45	20	34	—	29	—	—	Intervention 1: Tamoxifen 20 mg OD	6 months	Improved
														Intervention 2: L-Carnitine 1,000 mg OD		
														Intervention 3: Tamoxifen 20 mg OD + L-Carnitine 1,000 mg OD		
														Intervention 4: Placebo		
18	Fedder et al. (2014)	Double-blind RCT	Denmark	29	Oligospermia	70	32	—	—	—	34		4	1000 mg *P. granatum* + 764 mg *A. galanga*	90 days	Improved

19	Mehni et al. (2014)	Double-blind RCT	Iran	30 ± 4.6	Unexplained oligoasthenoteratozoospermia	235	53	49	51	—	59	—	23	Intervention 1: PX 400 mg OD + L-C 500 mg BD Intervention 2: PX 400 mg OD + placebo BD Intervention 3: L-C 500 mg BD + placebo BD Intervention 4: Placebo + placebo BD	3 months	Improved
20	Ismail et al. (2014)	Double-blind RCT	Malaysia	34 ± 4.87	Oligospermia	66	34	32	—	—	—	—	—	Intervention 1: Tribestan 750 mg OD Intervention 2: Tualang Honey 20 mg OD	12 weeks	Improved
21	Wiehle et al. (2014)	Double-blind RCT	The United States	50	Hypogonadism	124	20	23	23	—	14	—	51	Intervention1: 12.5 mg Enclomiphene citrate	3 months	Improved
														Intervention 2: 2.5 mg Enclomiphene citrate		
														Intervention 3: 1% Topical T		
22	Paradisi et al. (2006)	Open label RCT	Italy	-	Idiopathic infertility	60	45	—	—	—	15	—	—	rh-FSH 300 IU	4 months	Improved
23	Gopinath et al. (2013)	Double-blind RCT	India	32 ± 4	Idiopathic oligoasthenoteratozoospermia	138	46	43	—	—	36	—	13	Intervention 1: 2 tab FDC BD Intervention 2: 1 tab FDC +1 tab placebo	6 months	Improved
24	Ambiye et al. (2013)	Double-blind RCT	India	35.28 ± 5.49	Oligospermia	46	21	—	—	—	25	—	—	Ashwagandha (extract) 225 mg TID	90 days	Improved
25	Hadwan et al. (2012)	Open label RCT	Iraq	32	Asthenozoospermia	74	37	—	—	—	—	37	—	(Cap) Zinc sulphate 220 mg BD	3 months	Improved
26	Colacurci et al. (2012)	Open label RCT	Italy	31.6 ± 3.1	Idiopathic oligoasthenoteratozoospermia	65	—	—	—	—	—	64	—	Intervention: rFSH 150 IU OD	90 days	Improved
														Control: nonantioxidant vitamin supplements		

27	Imhof et al. (2012)	Open label RCT	Austria	34	Subfertile males	214	132	—	—	—	—	73	9	PROFERTIL 1tab OD	3 months	Improved
28	Safarinejad et al. (2012)	Double-blind RCT	Iran	31	Idiopathic infertility	228	101	—	—	—	102	—	25	Ubiquinol 200 mg OD	26 weeks	Improved
29	Mbah et al. (2012)	Open label RCT	Nigeria	26.93 ± 7.3	Idiopathic oligospermia	33	33	—	—	—	33	—	—	Lisinopril 2.5 mg OD	282 weeks	Improved
30	Nadjarzadeh et al. (2014)	Double-blind RCT	Iran	34.67 ± 6.67	Idiopathic oligoasthenoteratozoospermia	60	23	—	—	—	24	—	13	Coenzyme Q10 200 mg OD	3 months	No significant benefit
31	Kumar et al. (2011)	Double-blind RCT	India	32 ± 5.09	Idiopathic oligoasthenoteratozoospermia	25	—	—	—	—	25	—	6	.Addyzoa^®^ two capsules twice a day	3 months	Improved
32	Safarinejad, (2011a)	Double-blind RCT	Iran	32 ± 9	Idiopathic oligoasthenoteratozoospermia	265	119	—	—	—	119	—	27	Omega-3 1.84 g OD	32 weeks	Improved
33	Selice et al. (2011)	Single-blind RCT	Italy	—	Idiopathic infertility	105	70	—	—	—	35	—	—	150 IU rh-FSH/3 times a weeks	3 months	Improved
34	Mohammad Reza Safarinjad et al., 2010	Double-blind RCT	Iran	32.1 ± 4.3	Idiopathic infertility	254	114	—	—	—	115	—	25	PX 400 mg BD	24 weeks	Improved
35	Mohammad Reza Safarinejad et al. 2010	Double-blind RCT	Iran	28.6 ± 5.4	Idiopathic oligoasthenoteratozoospermia	260	114	—	—	—	116	—	30	Saffron 60 mg OD	26 weeks	No significant benefit
36	Moradi et al. (2010)	Double-blind RCT	Iran	28.5 ± 3.21	Idiopathic infertility	52	20	32	—	—	—	—	—	Intervention 1: clomiphene citrate 25 mg OD Intervention 2: L-Carnitine 2 g OD	3 months	Improved
37	Ghanem et al. (2010)	Double-blind RCT	Egypt	31.8 ± 8.1	Idiopathic infertility	60	30	—	—	—	30	—	—	Clomiphene citrate 25 mg OD + Vitamin E 400 mg OD	6 months	Improved
38	Safarinejad, (2009)	Open label RCT	Iran	28 ± 9	Idiopathic oligoasthenoteratozoospermia	212	98	—	—	—	96	—	18	Ubiquinone 300 mg OD	26 weeks	Improved
39	Safarinejad and Safarinejad, (2009)	Double-blind RCT	Iran	31	Idiopathic oligoasthenoteratozoospermia	468	105	105	104	—	106	—	48	Intervention 1: Se 200 ug OD	26 weeks	Improved
														Intervention 2: NAC 600 mg OD		
														Intervention 3: Se 200 ug OD + NAC 600 mg OD		
40	Çakan et al. (2009)	Single-blind RCT	Turkey	27.3 ± 4.9	Idiopathic oligoasthenoteratozoospermia	128	42	30	31	—	—	25	—	Intervention 1: Tamoxifen 10 mg BD (25 days a month)	6 months	Improved
														Intervention 2: Tamoxifen 10 mg BD (25 days a month for 6 months) + Anastrozole 1 mg OD (3 months starting from 3 to 6 months)		
														Intervention 3: Tamoxifen 10 mg BD (25 days a month)		
41	Farmakiotis et al. (2007)	Open label RCT	Greece	34	Oligozoospermia	100	—	—	—	—	—	—	—	Toremifene 60 mg OD	3 months	Improved
42	Nadjarzadeh et al. (2014)	Double-blind RCT	Iran	—	Idiopathic oligoasthenoteratozoospermia	60	23	—	—	—	24	—	13	CoQ10 100 mg BD	90 days	Improved
43	Sigman et al. (2006)	Double-blind RCT	The United States	36.2 ± 1.7	Idiopathic asthenospermia	26	12	—	—	—	9	—	5	L-Carnitine 2 g OD + acetyl L-Carnitine 1 g OD	24 weeks.	No significant benefit
44	Paradisi et al. (2014)	Double-blind RCT	Italy	—	Idiopathic oligoasthenoteratozoospermia	30	15	—	—	—	15	—	—	rhFSH 300IU OD	4 months	Improved
45	Foresta et al. (2005)	Double-blind RCT	Italy	35	Idiopathic infertility	128	62	—	—	—	50	—	16	rhFSH 100 IU on alternate days	3 months	No significant benefit
46	Balercia et al. (2005)	Double-blind RCT	Italy	30	Idiopathic asthenozoospermia	61	15	15	15	—	15	—	1	Intervention 1: L-Carnitine 3 g OD	6 months	Improved
														Intervention 2: L-Acetyl Carnitine 3 g OD Intervention 3: L-Carnitine 1 g OD + L Acetyl Carnitine 2 g OD		
47	Lenzi et al. (2004)	Double-blind RCT	Italy	30	Asthenozoospermia	60	30	—	—	—	26	—	4	Carnitine 2 g OD + L acetyl Carnitine 1 g OD	6 months	Improved
48	Caroppo et al. (2003)	Open label RCT	Italy	35.3 ± 4.9	Idiopathic oligoasthenoteratozoospermia	33	23	—	—	—	—	10	—	r-hFSH 150 IU IM 3 times/week	3 months	Improved
49	Adamopoulos et al. (2003)	Open label RCT	Greece	36	Idiopathic oligozoospermia	294	106	—	—	—	106	82	—	Tamoxifen citrate 20 mg OD + Testosterone undecanoate 120 mg OD	6 months	Improved
50	Lenzi et al. (2003)	Double-blind crossover study	Italy	30	Oligospermia	100	86	—	—	—	—	—	14	L-Carnitine 2 g OD	4 months	Improved
51	Wong et al. (2002)	Double-blind RCT	South Africa	34.3 ± 3.9	Subfertile males	193	46	49	49	—	49	—	19	Intervention 1: Folic acid 5 mg OD + placebo, Intervention 2: zinc 66 mg OD + placebo, Intervention 3: zinc 66 mg OD + folic acid 5 mg	26 weeks	Improved
52	Foresta et al. (2002)	Single-blind RCT	Italy	32.6 ± 4.5	Idiopathic oligozoospermia	45	15	15	—	—	—	15	—	Intervention 1: rhFSH 50IU OD Intervention 2: rhFSH 100IU OD	3 months	Improved
53	Matsumiya et al. (1998)	Open label RCT	Japan	30	Idiopathic oligoasthenozoospermia	44	23	21	—	—	—	—	—	Intervention 1: Buserelin acetate 15 ug OD, Intervention2: Clomiphene citrate 50 mg OD	3 months	Improved
54	Badenoch et al. (1988)	Double-blind RCT	England	32.6 ± 1.1	Subfertile males	69	16	30	—	—	18	—	5	Intervention 1: Selenomethionine 100 mg OD	3 months	Improved
														Intervention 2: Selenium + vitamins		
55	Kamischke et al. (1998)	Double-blind RCT	Germany	32.89 ± 0.56	Idiopathic infertility	67	34	—	—	—	31	—	2	Intervention 1: 150 IU rhFSH	12 weeks	Improved
56	Merino et al. (1997)	Open label RCT	Mexico	30.8 ± 6	Asthenospermia	47	25	—	—	—	22	—	—	PX 400 mg TID	6 months	Improved
57	Adamopoulos et al. (1997)	Double-blind RCT	Greece	37	Idiopathic oligozoospermia	80	18	20	20	—	18	—	4	Intervention 1: Tamoxifen citrate 10 mg BD Intervention 2: Testosterone undecanoate 40 mg TID	6 months	Improved
														Intervention 3: Both interventions 1 and 2 combined		
58	Suleiman et al. (1996)	Double-blind RCT	Saudi Arabia	36	Asthenospermia	110	52	—	—	—	35	—	23	vitamin E 10 mg TID	6 months	No significant benefit
59	Yamamoto et al. (1995b)	Single-blind RCT	Japan	—	Idiopathic oligozoospermia	50	21	—	—	—	25	—	4	Tranilast 300 mg OD	3 months	Improved
60	Yamamoto et al. (1995a)	Double-blindRCT	Japan	33	Idiopathic oligozoospermia	31	16	—	—	—	15	—	—	Bunazosin 4 mg OD	6 months	Improved
61	Keck et al. (1994)	Double-blind RCT	Germany	32 ± 4.9	Idiopathic male infertility	91	44	—	—	—	47	—	4	Kallikrein 600 IU OD (porcine origin)	12 weeks	No significant benefit
62	Glezerman et al. (1993)	Double-blind RCT	Israel	33	Oligozoospermia & asthenozoospermia	140	52	—	—	—	57	—	31	Kallikrein 600IU OD	3 months	No significant benefit
63	Crottaz et al. (1992)	Double-blind RCT	Switzerland	32.9 ± 1.1	Idiopathic asthenozoospermia	39	14	—	—	—	14	—	11	GnRH 0.2 mg/ml IN every 2 h from 8am to 8 pm	3 months	No significant benefit
64	Gerris et al. (1991)	Double-blindRCT	The United States	—	Idiopathic male infertility	60	27	—	—	—	25	—	8	Mesterolone 150 mg OD	12 months	Improved
65	Maier and Hienert, (1990)	Open label RCT	Austria	24	Oligoasthenozoospermia	67	33	34	—	—	—	—	—	Intervention 1: Tamoxifen 30 mg ODIntervention 2: Tamoxifen 30 mg OD + Kallikrein 600IU/d	3 months	Improved

66	Clark and Sherins, (1989)	Double-blind crossover study	The United States	NA	Oligozoospermia	33	25	—	—	—	—	—	8	4 × 500 mg Testolactone	16 months	No significant benefit
67	Badenoch et al. (1988)	Open label RCT	England	NA	Oligozoospermia	19	7	8	—	—	—	4	—	Intervention1: 2 X 1ug Buserelin (weekly) Intervention2:2 × 10 ug Buserelin (weekly)	12 weeks	No significant benefit
68	Pusch, (1988)	Double-blind RCT	Austria	NA	Oligozoospermia	60	29	—	—	—	28	—	3	Testosterone undecanoate 40 mg TID	12 weeks	Improved
69	Knuth et al. (1987)	Double-blind RCT	Germany	32	Oligospermia	39	17	—	—	—	20	—	2	2500 IU hCG (twice weekly) + 150IU hMG (trice weekly)	13 weeks	No significant benefit
70	AinMelk et al. (1987)	Double-blind crossover study	Canada	29	Oligozoospermia	19	16	—	—	—	—	—	3	20 mg Tamoxifen	12 months	Improved
71	Haas and Manganiello, (1987)	Double-blindRCT	The United States	NA	Antibody-mediated infertility	43	20	—	—	—	15	—	8	Methylprednisolone 32 mg TID (7days)	3 menstrual cycles	Improved
72	Ding et al. (2015)	Double-blind RCT	Switzerland	31	Accessory gland infection	33	20	—	—	—	13	—	—	Doxycycline 100 mg OD	1 month	No significant benefit
73	Mićić and Dotlić, (1985)	Open label RCT	Yugoslavia	NA	Oligospermia	101	56	—	—	—	—	45	—	50 mg Clomiphene citrate	6–9 months	Improved
74	Almeida et al. (1985)	Double-blind RCT	France	30	Immune-related infertility	10	5	—	—	—	5	—	—	1 mg/kg Corticosteroid (initial dose 60–80 mg)	20 days for 3 menstrual cycles	Improved
75	Hargreave et al. (1984)	Open label RCT	Scotland	28	Oligospermia	368	152	176	—	—	—	—	40	Intervention1: 200 mg Vit.C Intervention2: 2 × 50 mg Mesterolone	9 months	Improved
76	Baker et al. (1984)	Double-blind c rossover study	Australia	NA	Asthenospermia	100	40	—	—	—	38		22	Erythromycin 250 mg OD	4 months	No significant benefit
77	Izzo et al. (1984)	Double-blind RCT	Italy	NA	Oligozoospermia & asthenozoospermia	30	15	—	—	—	14		1	Kallikrein 600 IU OD	3 months	Improved
78	Inton et al. (1979)	Double-blind crossover study	England	NA	Infection induced infertility	42	21	—	—	—	21	—	—	Doxycycline 100 mg OD and Doxycycline 200 mg OD	10 days course each in 3 menstrual cycles	No significant benefit
79	Pryor et al. (1978)	Double-blind crossover study	England	NA	Oligozoospermia	64	54	—	—	—	—	—	10	Arginine 4 g OD	24 weeks	No significant benefit
80	Willis et al. (1977)	Single-blind RCT	England	NA	Oligospermia	9	9	—	—	—	—	—	—	Tamoxifen 120 mg OD	6 months	No significant benefit

Se, Selenium; NAC, n-acetyl cysteine; L-C, L-Carnitine, PX, pentoxifylline; RCT, randomized controlled trial; OD, once daily; BD, twice daily.

## Risk of Bias

**FIGURE 2:**
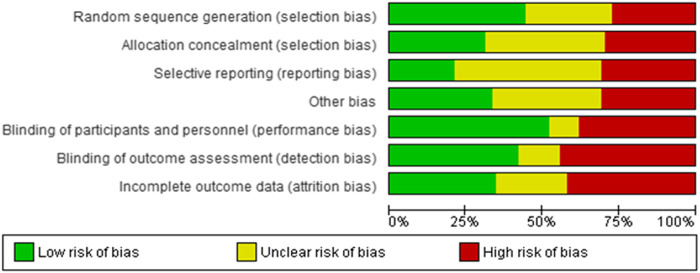
Overall risk of bias.

**FIGURE 3:**
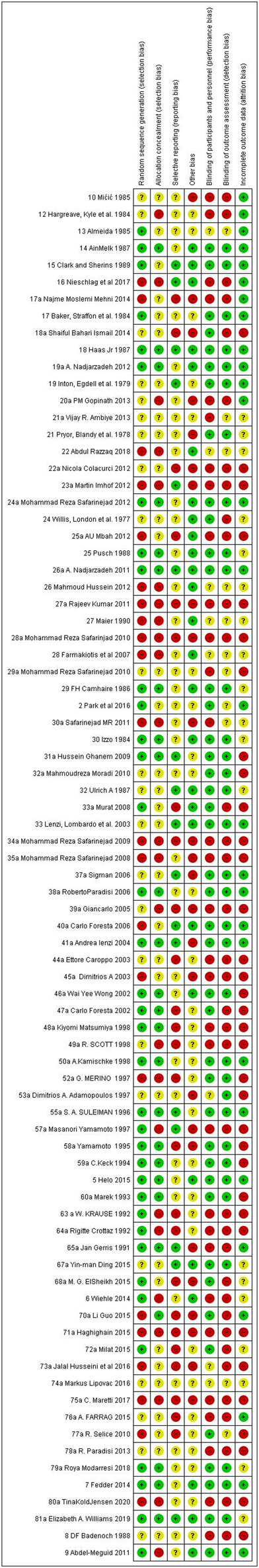
Summary of risk of bias for individual studies.

Figures 2 and 3 present ROB for all the included RCTs. Cochrane ROB tool was used to generate the graphs. More than 20% of the studies were categorized as free of attrition bias, reporting bias and other sources of bias. Performance bias and selection bias were granted in only 40 and 35% of the studies, respectively.

## Primary Outcomes

Sperm concentration (10^6^/ml), sperm motility (%), and sperm morphology (%) were the three primary clinical outcomes discussed in this study. A total of n = 29 studies were included for quantitative synthesis (Mićić and Dotlić, 1985; AinMelk et al., 1987; Pusch, 1988; Krause et al., 1992; Keck et al., 1994; Kamischke et al., 1998; Foresta et al., 2002; Wong et al., 2002; Adamopoulos et al., 2003; Caroppo et al., 2003; Lenzi et al., 2004; Paradisi et al., 2006; Safarinejad, 2009; Çakan et al., 2009; Ghanem et al., 2010; Safarinejad, 2011b; Selice et al., 2011; Colacurci et al., 2012; Hadwan et al., 2012; Imhof et al., 2012; Safarinejad et al., 2012; Nadjarzadeh et al., 2014; Paradisi et al., 2014; Ding et al., 2015; Farrag et al., 2015; Haje and Naoom, 2015; Lipovac et al., 2016; Alsalman et al., 2018; Jensen et al., 2020). All the studies were randomized control trials and encompass data on the stated outcomes involving interventions grouped under categories like hormones, selective estrogen receptor modulators, supplements, vitamins, and enzymes (Supplementary Figures 1–3).

## Sperm Concentration

Pairwise MA results were in the favor of the SERMs (Mićić and Dotlić, 1985; AinMelk et al., 1987; Krause et al., 1992; Çakan et al., 2009; Ghanem et al., 2010; Haje and Naoom, 2015) where the levels of sperm concentration increased to 6.00 million per mL [95% CI 5.27, 6.72; *p* = 0.43] followed by supplements [5.99; 95% CI 2.83, 9.15; *p* < 0.00001; I^2^ = 91%] (Wong et al., 2002; Lenzi et al., 2004; Safarinejad, 2009; Nadjarzadeh et al., 2011; Hadwan et al., 2012; Imhof et al., 2012; Safarinejad et al., 2012; Nadjarzadeh et al., 2014; Haje and Naoom, 2015; Lipovac et al., 2016; Alsalman et al., 2018; Jensen et al., 2020) (Supplementary Figure 4).

Subgroup analysis of studies examining the effect of SERMs revealed (Mićić and Dotlić, 1985; AinMelk et al., 1987; Krause et al., 1992; Çakan et al., 2009; Ghanem et al., 2010; Haje and Naoom, 2015) maximum effect with clomiphene citrate [6.91; 95% CI 5.62, 8.20; *p* = 0.95; I^2^ = 0%] (Mićić and Dotlić, 1985; Ghanem et al., 2010), followed by tamoxifen citrate [5.61; 95% CI 4.75, 6.46; *p* = 0.50; I^2^ = 0%] (AinMelk et al., 1987; Krause et al., 1992; Çakan et al., 2009; Haje and Naoom, 2015) (Supplementary Figure 5).

Moreover, the subgroup analysis for supplements (Wong et al., 2002; Lenzi et al., 2004; Safarinejad, 2009; Nadjarzadeh et al., 2011; Hadwan et al., 2012; Imhof et al., 2012; Safarinejad et al., 2012; Nadjarzadeh et al., 2014; Haje and Naoom, 2015; Lipovac et al., 2016; Alsalman et al., 2018; Jensen et al., 2020) revealed that studies involving zinc sulfate (Wong et al., 2002; Hadwan et al., 2012; Alsalman et al., 2018) had large effect [11.49; 95% CI -6.42, 29.39; *p* = 0.001; I^2^ = 81%] followed by Profertil [10.90; 95% CI 7.98, 13.82] (Imhof et al., 2012) and CoQ10 [6.53; 95% CI 1.88, 11.17; *p* < 0.00001; I^2^ = 94%] (Safarinejad, 2009; Nadjarzadeh et al., 2011; Safarinejad et al., 2012; Nadjarzadeh et al., 2014), respectively. Statistically significant results were seen in CoQ10 (Supplementary Figure 6).

## Sperm Motility

Pairwise MA results were in the favor of the SERMs (Mićić and Dotlić, 1985; AinMelk et al., 1987; Krause et al., 1992; Çakan et al., 2009; Ghanem et al., 2010; Haje and Naoom, 2015) where the percentage of sperm motility was increased [6.62; 95% CI 3.69, 9.54; *p* = 0.005; I^2^ = 63%] followed by supplements (Wong et al., 2002; Lenzi et al., 2004; Safarinejad, 2009; Nadjarzadeh et al., 2011; Hadwan et al., 2012; Imhof et al., 2012; Safarinejad et al., 2012; Nadjarzadeh et al., 2014; Haje and Naoom, 2015; Lipovac et al., 2016; Alsalman et al., 2018; Jensen et al., 2020) with a standard mean difference of 6.51 [95% CI 3.15, 9.86; *p* < 0.00001; I^2^ = 96%] (Supplementary Figure 7).

Subgroup analysis revealed that among SERMs (Mićić and Dotlić, 1985; AinMelk et al., 1987; Krause et al., 1992; Çakan et al., 2009; Ghanem et al., 2010; Haje and Naoom, 2015) large effects were made by studies which involved clomiphene citrate [8.17; 95% CI 425, 12.10; *p* = 0.79; I^2^ = 0%] (Mićić and Dotlić, 1985; Ghanem et al., 2010) followed by tamoxifen citrate [6.26; 95% CI 2.62, 9.90; *p* = 0.002; I^2^ = 71%] (AinMelk et al., 1987; Krause et al., 1992; Çakan et al., 2009; Haje and Naoom, 2015) (Supplementary Figure 8).

Moreover, the subgroup analysis for supplements (Wong et al., 2002; Lenzi et al., 2004; Safarinejad, 2009; Nadjarzadeh et al., 2011; Hadwan et al., 2012; Imhof et al., 2012; Safarinejad et al., 2012; Nadjarzadeh et al., 2014; Haje and Naoom, 2015; Lipovac et al., 2016; Alsalman et al., 2018; Jensen et al., 2020) revealed that studies involving zinc sulfate (Wong et al., 2002; Hadwan et al., 2012; Alsalman et al., 2018) had large effect [16.78; 95% CI 14.27, 19.29; *p* = 0.76; I^2^ = 0%] followed by Profertil [7.00; 95% CI -1.50, 15.50] (Imhof et al., 2012) and CoQ10 [6.97; 95% CI 1.94, 12.01; *p* < 0.00001; I^2^ = 98%] (Safarinejad, 2009; Nadjarzadeh et al., 2011; Safarinejad et al., 2012; Nadjarzadeh et al., 2014), respectively (Supplementary Figure 9).

## Sperm Morphology

Pairwise MA results were in the favor of the hormones (Pusch, 1988; Kamischke et al., 1998; Foresta et al., 2002; Adamopoulos et al., 2003; Caroppo et al., 2003; Paradisi et al., 2006; Selice et al., 2011; Colacurci et al., 2012; Paradisi et al., 2014; Ding et al., 2015; Farrag et al., 2015) where the percentage increase in sperm morphology was maximum [3.68; 95% CI 0.97, 6.39; *p* < 0.00001; I^2^ = 83%], followed by supplements [1.93; 95% CI 0.43, 3.43; *p* < 0.00001; I^2^ = 89%] (Wong et al., 2002; Lenzi et al., 2004; Safarinejad, 2009; Nadjarzadeh et al., 2011; Hadwan et al., 2012; Imhof et al., 2012; Safarinejad et al., 2012; Nadjarzadeh et al., 2014; Haje and Naoom, 2015; Lipovac et al., 2016; Alsalman et al., 2018; Jensen et al., 2020) (Supplementary Figure 10).

Subgroup analysis suggested that among hormones favourable effect was made by testosterone [12.24; 95% CI 1.00, 23.49; *p* = 0.01; I^2^ = 83%] (Pusch, 1988; Adamopoulos et al., 2003), followed by FSH at a dose range of ≥200–300 IU [6.53; 95% CI -0.54, 5.59; *p* = 0.99; I^2^ = 0%] (Paradisi et al., 2006; Paradisi et al., 2014; Ding et al., 2015) (Supplementary Figure 11).

Moreover, the subgroup analysis for supplements revealed that Profertil has large effect [14.50; 95% CI 7.31, 21.69] (Imhof et al., 2012) followed by zinc sulfate [4.23; 95% CI -2.39, 10.84; *p* < 0.0001; I^2^ = 86% ] (Wong et al., 2002; Hadwan et al., 2012; Alsalman et al., 2018) (Supplementary Figure 12).

## Secondary Outcomes

Serum total testosterone (ng/ml) and serum FSH (mIU/ml) were two secondary clinical outcomes discussed in this study. Total n = 12 studies were included (AinMelk et al., 1987; Pusch, 1988; Krause et al., 1992; Kamischke et al., 1998; Paradisi et al., 2006; Safarinejad, 2009; Çakan et al., 2009; Selice et al., 2011; Safarinejad et al., 2012; Paradisi et al., 2014; Helo et al., 2015; Jensen et al., 2020). All the studies were randomized control trials involving interventions grouped under categories like hormones, selective estrogen receptor modulators, supplements, vitamins, and enzymes (Supplementary Figures 13 and 14).

## Total Serum Testosterone

Pairwise MA results were in the favor of the supplements (Safarinejad and Safarinejad, 2009; Safarinejad, 2011a; Jensen et al., 2020) where the total serum testosterone concentration was increased [2.74; 95% CI 1.81, 3.68; *p* = 0.78; I^2^ = 0%] followed by SERMs [1.50; 95% CI 1.20, 1.79; *p* < 0.00001; I^2^ = 92%] (AinMelk et al., 1987; Krause et al., 1992; Çakan et al., 2009) (Supplementary Figure 15).

Subgroup analysis revealed that among supplements (Safarinejad and Safarinejad, 2009; Safarinejad, 2011a; Jensen et al., 2020) coenzyme Q10 ^34,39^ showed better results in increasing total serum testosterone concentration [2.77; 95% CI 1.83, 3.71; *p* = 0.76; I^2^ = 0%]. None of remaining supplements showed substantial effects (Supplementary Figure 16).

Moreover, the subgroup analysis for SERMs (AinMelk et al., 1987; Krause et al., 1992; Çakan et al., 2009) showed that all the total serum testosterone concentration effect was due to tamoxifen citrate [1.50; 95% CI 1.20, 1.79; *p* < 0.00001; I^2^ = 92%] (AinMelk et al., 1987; Krause et al., 1992; Çakan et al., 2009); none of the studies reported clomiphene citrate (Supplementary Figure 17).

## Total Serum Follicle Stimulating Hormone

Pairwise MA results were in the favor of the SERMs (AinMelk et al., 1987; Krause et al., 1992; Çakan et al., 2009) where the total serum FSH concentration was increased [3.66; 95% CI 1.27, 6.05; *p* < 0.00001; I^2^ = 90%] followed by hormones [1.24; 95% CI -0.29, 2.77; *p* < 0.00001; I^2^ = 89%] (Supplementary Figure 18).

Subgroup analysis revealed that among SERMS studies only tamoxifen citrate 1.05 [95% CI 0.40, 1.71; *p* < 0.0001; I^2^ = 84%] (AinMelk et al., 1987; Krause et al., 1992; Çakan et al., 2009) effect was reported. None of the studies reported clomiphene citrate (Supplementary Figure 19).

Moreover, the subgroup analysis for hormones (Pusch, 1988; Kamischke et al., 1998; Paradisi et al., 2006; Selice et al., 2011; Paradisi et al., 2014; Helo et al., 2015) revealed that studies involving FSH in dose <200–50 IU) (Kamischke et al., 1998; Selice et al., 2011) showed better results in increasing total serum FSH concentration [2.74; 95% CI -0.00, 5.48; *p* = 0.0009; I^2^ = 91%] followed by studies involving FSH in dose ≥200–300IU [0.94; 95% CI -0.82, 2.70; *p* = 0.03; I^2^ = 80%] (Paradisi et al., 2006; Paradisi et al., 2014) (Supplementary Figure 20).

## Network Meta-Analysis

**FIGURE 4:**
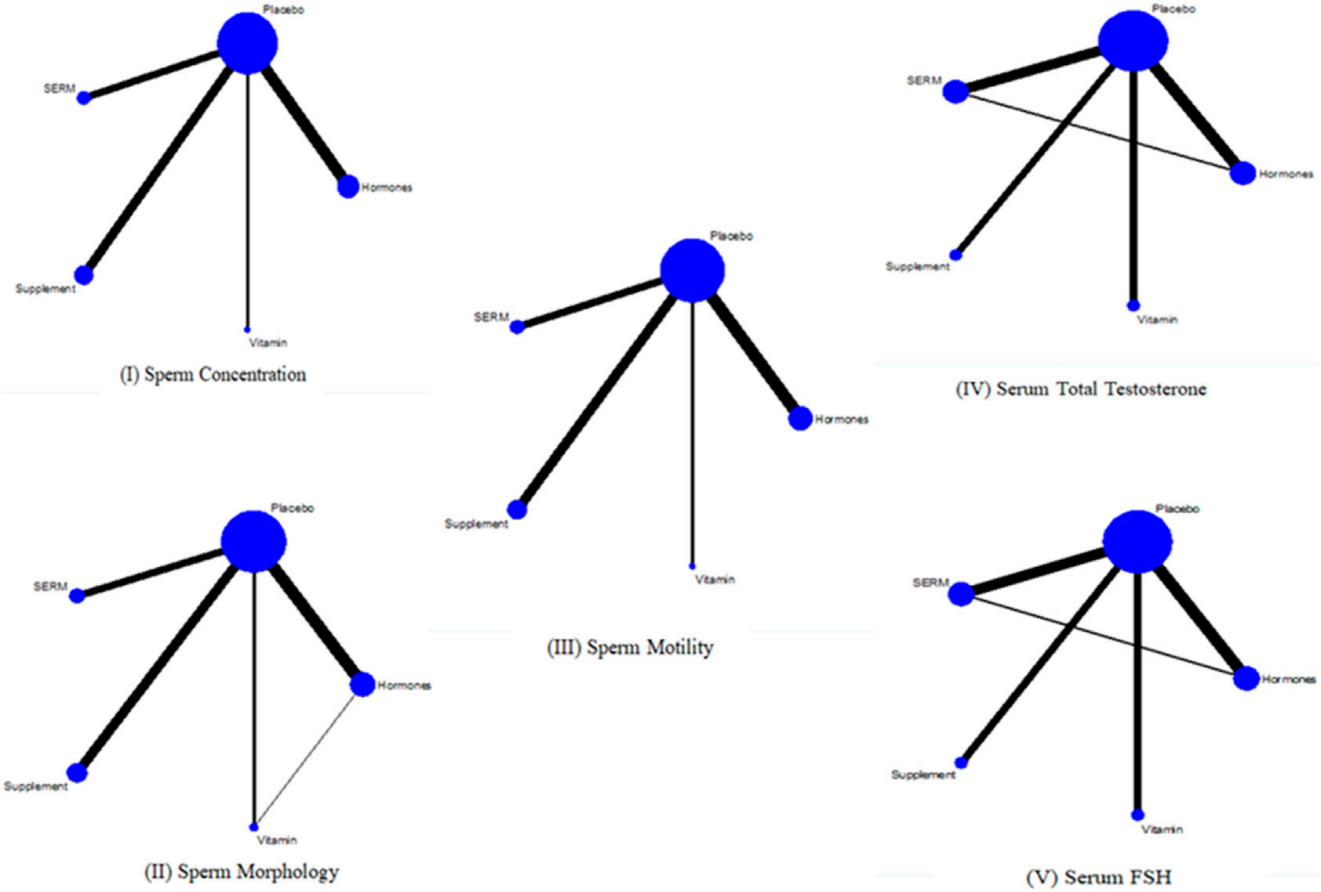
Network plots I, II, and III are for primary outcomes whereas IV and V are network plots for secondary outcomes.

To assess the effect of intervention on primary and secondary parameters, twenty-eight studies (Mićić and Dotlić, 1985; AinMelk et al., 1987; Pusch, 1988; Krause et al., 1992; Kamischke et al., 1998; Foresta et al., 2002; Wong et al., 2002; Adamopoulos et al., 2003; Caroppo et al., 2003; Lenzi et al., 2003; Paradisi et al., 2006; Safarinejad, 2009; Çakan et al., 2009; Ghanem et al., 2010; Nadjarzadeh et al., 2011; Selice et al., 2011; Colacurci et al., 2012; Hadwan et al., 2012; Imhof et al., 2012; Safarinejad et al., 2012; Nadjarzadeh et al., 2014; Paradisi et al., 2014; Ding et al., 2015; Farrag et al., 2015; Haje and Naoom, 2015; Lipovac et al., 2016; Alsalman et al., 2018; Jensen et al., 2020) were included for primary outcomes and seventeen studies (AinMelk et al., 1987; Pusch, 1988; Krause et al., 1992; Kamischke et al., 1998; Paradisi et al., 2006; Safarinejad, 2009; Çakan et al., 2009; Selice et al., 2011; Safarinejad et al., 2012; Paradisi et al., 2014; Helo et al., 2015; Jensen et al., 2020) were included for secondary outcomes. NMA was executed to compare the effects of intervention on primary and secondary parameters. The network plots exhibit the association of all available evidences. The thickness of the lines denotes the number of trials and the size of node represents the sample size (Figure 4).

With respect to primary outcomes (sperm concentration, sperm morphology, and sperm motility) when the reference arm was set as a placebo in the analysis, the sperm concentration was found to be considerably higher for supplement 6.26 [CI 95% 3.32, 9.21; *p* = 0.00] followed by SERMs 4.97 [CI 95% 1.61, 8.32; *p* = 0.004] and hormone 4.14 [CI 95% 1.83, 6.45; *p* = 0.00] groups versus the placebo group. On the other hand, the sperm morphology was significantly increased in hormone 3.71 [CI 95% 1.34, 6.07; *p* = 0.002] followed by supplement 2.22 [CI 95% −0.12, 4.55; *p* = 0.63] and SERMs 2.21 [CI 95% -0.78, 5.20; *p* = 0.15] groups versus the placebo group. Moreover, increasing trends in sperm motility were observed in SERMs 6.69 [CI 95% 2.38, 10.99; *p* = 0.002] trailed by supplement 6.46 [CI 95% 2.86, 10.06; *p* = 0.00] and hormone 3.47 [CI 95% 0.40, 6.54; *p* = 0.027] groups versus the placebo group (Table 3).

**TABLE 3:** Network meta-analysis for impact various pharmacological group interventions on primary and secondary clinical outcomes of male infertility.

Outcome parameter	Intervention vs. placebo	WMD [95% CI]	SE	z	*p*-value
Sperm concentration	Hormones	4.14 [1.83, 6.45]	1.18	3.51	0.000
	Selective estrogen receptor modulator (SERM)	4.97 [1.61, 8.32]	1.71	2.90	0.004
	Supplement	6.26 [3.32, 9.21]	1.50	4.16	0.000
	Vitamins	0.15 [−20.86, 21.15]	10.72	0.01	0.989
Sperm morphology	Hormones	3.71 [1.34, 6.07]	1.20	3.07	0.002
	Selective estrogen receptor modulator (SERM)	2.21 [−0.78, 5.20]	1.53	1.45	0.15
	Supplement	2.22 [−0.12, 4.55]	1.19	1.86	0.063
	Vitamins	0.51 [−3.60, 4.62]	2.09	0.24	0.808
Sperm motility	Hormones	3.47 [0.40, 6.54]	1.56	2.22	0.027
	Selective estrogen receptor modulator (SERM)	6.69 [2.38, 10.99]	2.19	3.04	0.002
	Supplement	6.46 [2.87, 10.06]	1.83	3.52	0.000
	Vitamins	−1.21 [−11.84, 9.42]	5.42	−0.22	0.824
Serum total testosterone	Hormones	0.40 [−0.48, 1.28]	0.45	0.88	0.377
	Selective estrogen receptor modulator (SERM)	1.83 [1.16, 2.50]	0.34	5.25	0.000
	Supplement	2.70 [1.34, 4.07]	0.69	3.87	0.000
	Vitamins	−0.70 [−6.71, 5.31]	3.06	−0.23	0.819
Serum follicle stimulating hormone (FSH)	Hormones	1.29 [−0.79, 3.36]	1.05	1.22	0.223
	Selective estrogen receptor modulator (SERM)	3.63 [1.480, 5.785]	1.09	3.31	0.001
	Supplement	−4.45 [−7.149, −1.758]	1.37	−3.24	0.001
	Vitamins	0.033 [−2.692, 2.760]	1.39	0.02	0.981

For secondary outcomes (serum total testosterone and serum FSH) by setting the reference arm as placebo in the analysis serum total testosterone was significantly increased in supplement 2.70 [CI 95% 1.33, 4.07; *p* = 0.00] followed by SERMS 1.82 [CI 95% 1.15, 2.49; *p* = 0.00] and hormone 0.40 [CI 95% -0.48, 1.28; *p* = 0.377] whereas serum FSH was significantly increased in SERMs 3.63 [CI 95% 1.48, 5.78; *p* = 0.001] followed by hormones 1.28 [CI 95% −0.78, 3.36; *p* = 0.223] and vitamins 0.033 [CI 95% −2.69, 2.76; *p* = −2.692] groups versus the placebo group (Table 3).

League table was created by using NMA to elaborate all probable pairwise comparisons between any two of the four interventions and customary pairwise meta-analysis (Table 3). It was obvious from the NMA that all of four interventions show analogous efficacy in increasing primary outcomes (sperm concentration, sperm morphology, and sperm motility) and secondary outcomes (serum total testosterone and serum FSH) (Table 4).

**TABLE 4:** League table.

**Intervention effect on sperm concentration (10^6^/ml)**				
Supplement				5.99 (2.83, 9.15)
2.12 (−1.63,5.87)	Hormone			4.14 (2.30, 5.99)
1.30 (−3.16, 5.76)	−0.82 (−4.90, 3.25)	SERM		6.00 (5.27, 6.72)
6.12 (−15.10, 27.33)	3.99 (−17.14, 25.13)	4.82 (−16.45, 26.09)	Vitamin	0.14 (−20.41, 20.69)
6.26 (3.32, 9.21)	4.14 (1.83, 6.46)	4.97 (1.61, 8.32)	0.15 (−20.86, 21.15)	Placebo

Intervention effect on sperm motility (%)				
Supplement				6.51 (3.15, 9.86)
2.99 (−1.73, 7.71)	Hormone			3.55 (1.14, 5.96)
−0.23 (−5.83, 5.38)	−3.22 (−8.50, 2.07)	SERM		6.62 (3.69, 9.54)
7.67 (−3.56, 18.90)	4.68 (−6.39, 15.75)	7.9 (−3.58, 13.37)	Vitamin	−1.36 (−10.28, 7.54)
6.46 (2.57, 10.06)	3.47 (0.40, 6.54)	6.69 (2.38, 10.99)	−1.24 (−11.84, 9.43)	Placebo

Intervention effect on sperm morphology (%)				
Supplement				1.93 (0.43, 3.43)
−1.49 (−4.82, 1.83)	Hormone			3.68 (0.97, 6.39)
0.01 (−3.77, 3.79)	1.50 (−2.31, 5.31)	SERM		0.88 (0.07, 1.69)
1.71 (−3.03, 6.44)	3.20 (−1.23, 7.63)	1.70 (−3.39, 6.78)	Vitamin	−0.11 (−2.47, 2.24)
2.22 (0.12, 4.55)	3.71 (1.34, 6.07)	2.21 (−0.78, 5.20)	0.51 (−3.60, 4.62)	Placebo

Intervention effect on total serum testosterone (ng/ml)				
Supplement				2.74 (1.81, 3.68)
2.30 (0.67, 3.94)	Hormone			0.04 (−0.86, 0.94)
0.88 (−0.65, 2.40)	−1.43 (−2.44, −0.41)	SERM		1.50 (1.20, 1.79)
3.40 (−2.76, 9.57)	1.10 (−4.98, 7.18)	2.53 (−3.52, 8.58)	Vitamin	−0.70 (−6.66, 5.26)
2.70 (1.34, 4.07)	0.40 (−0.49, 1.29)	1.83 (1.16, 2.50)	−0.70 (−6.71, 5.31)	Placebo

Intervention effect on serum FSH (mIU/ml)				
Supplement				−4.44 (−8.36, −0.52)
−5.74 (−9.14, −2.34)	Hormone			1.24 (−0.29, 2.77)
−8.09 (−11.54, −4.64)	−2.34 (−5.32, 0.63)	SERM		3.66 (1.27, 6.05)
−4.49 (−8.32, −0.65)	1.25 (−2.17, 4.68)	3.60 (0.13, 7.07)	Vitamin	0.04 (−0.74, 0.82)
−4.45 (−7.15, −1.76)	1.29 (−0.79, 3.36)	3.63 (1.48, 5.79)	0.03 (−2.69, 2.76)	Placebo

**FIGURE 5:**
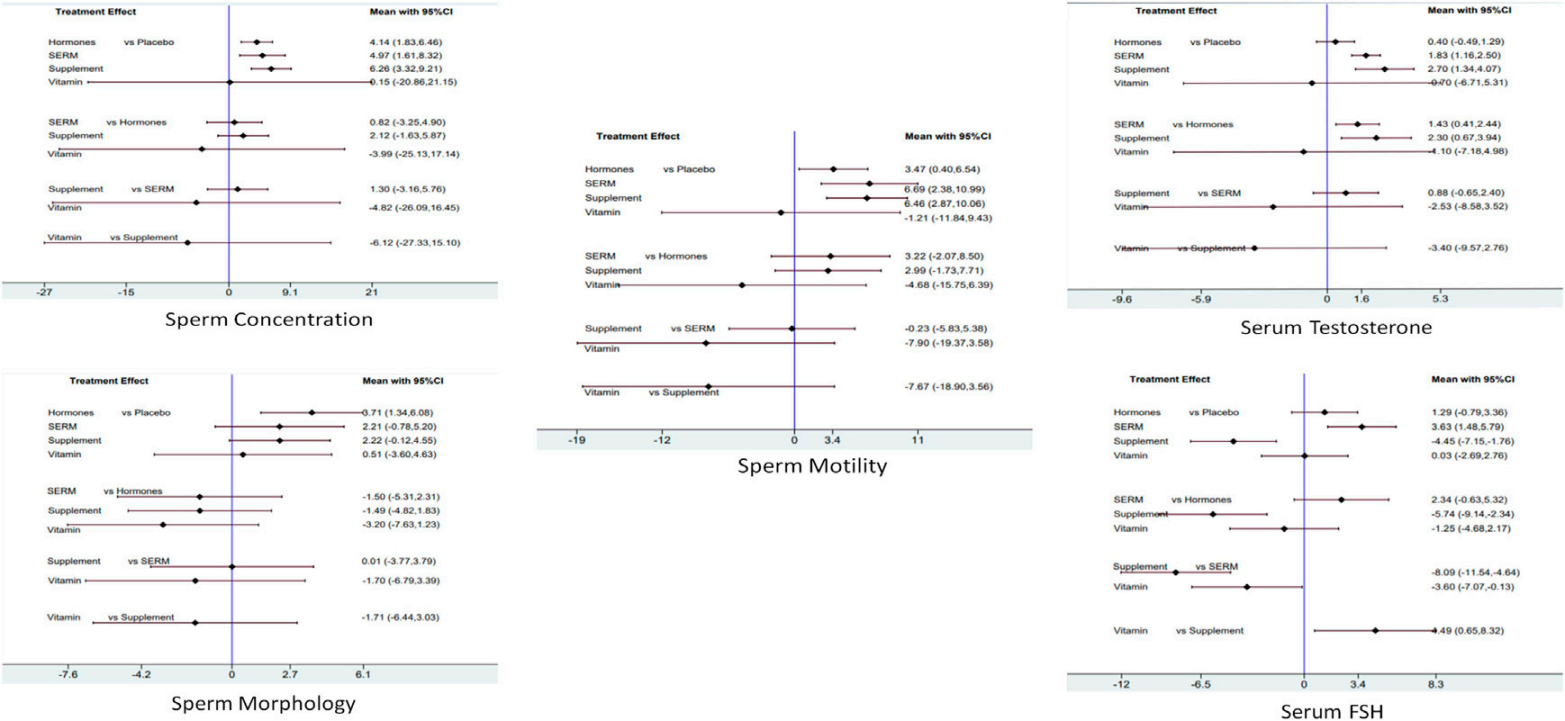
Network meta-analysis estimates of change in primary and secondary clinical outcomes of infertile male patients. Sperm concentration, sperm morphology, sperm motility, serum total testosterone, and serum FSH.

For primary outcomes, supplements were found statistically significant in increasing the sperm concentration 6.26 [CI 95%, 3.32, 9.21; *p* = 0.00] followed by SERM 4.97 [CI 95%, 1.61, 8.32; *p* = 0.004] and hormones 4.14 [CI 95%, 1.83, 6.46; *p* = 0.00]. Sperm motility was significantly increased by SERM 6.69 [CI 95%, 2.38, 10.99; *p* = 0.002] followed by supplements 6.46 [CI 95%, 2.87, 10.06; *p* = 0.00] and hormones 3.47 [CI 95%, 0.40, 6.54; *p* = 0.027]. Moreover, hormones proved to be statistically significant in improving the sperm morphology 3.71 [CI 95%, 1.34, 6.07; *p* = 0.002], followed by supplements 2.22 [CI 95%, 0.12, 4.55; *p* = 0.063] and SERMS 2.21 [CI 95%, −0.78, 5.20; *p* = 0.15] (Figure 5).

For secondary outcomes, supplements were found statistically significant in increasing serum total testosterone concentration 2.70 [CI 95%, 1.34, 4.07; *p* = 0.00] followed by SERMS 1.83 [CI 95%, 1.16, 2.50; *p* = 0.00] and hormones 0.40 [CI 5%, −0.49, 1.29; *p* = 0.37]. Moreover, SERMS proved to be statistically significant in increasing the serum FSH concentration 3.63 [CI 95%, 1.48, 5.79; *p* = 0.001], followed by hormones 1.29 [CI 95%, −0.79, 3.36; *p* = 0.223] and vitamins 0.03 [CI 95%, −2.69, 2.76; *p* = −2.692] (Figure 5).

## Strengths and Limitations

Numerous curbs are associated with this study. Due to lack of resources, non-English studies were not reviewed as it was difficult to translate them to other languages. Combination of data from non-English literature might alter the significance of the current analysis of various male infertility interventions. Secondly, there were few studies with different subgroups making it difficult to get a perfect picture of the overall comparison. In addition, heterogeneity among the one‐on‐one and pairwise comparison was in excess of 70%. However, subgroup analysis and removal of poor quality studies from NMA resolved this issue to some extent but it is still a limitation in this study. Also, population of interest, intervention, comparators, and outcomes were the same across all the studies included in the NMA. Therefore, the chance of clinical heterogeneity is at very minimal to negligible level ruling out statistical heterogeneity. Lastly, due to diversified types of the male infertility along with interventions, all such interventions were classified into five categories, i.e., supplements, hormones, SERMs, vitamins, and enzymes. Along with all the limitations, our NMA is the first study estimating and establishing comparison among all available interventions regarding male infertility and this comprises a very significant aspect of this work.

## Discussion

This review is the first of its kind to present NMA on the comparative effect of numerous interventions from different pharmacological groups in managing males with infertility, conducted worldwide, in diverse health care settings under different experimental practices.

The studies included in this review encompass different RCTs conducted using moieties from different pharmacological groups including hormones (FSH, testosterone, and anastrozole), selective estrogen receptor modulators (SERMs) (clomiphene citrate and tamoxifen citrate), supplements (zinc sulfate, CoQ10, carnitine, L-Carnitine, fish oil, and Profertil), vitamins (folic acid, vitamin C, vitamin D, and vitamin E), and enzymes (kallikrein). It is evident from the studies that concomitant administration of supplements, hormones, and SERMS in a patient with male infertility can enhance the production of healthy motile sperms through maintaining adequate serum FSH and testosterone levels. The average duration of clinical outcome was reported to be six months. However, it should be noted that no major side effects were reported from any of the included studies. Common side effects were mild GIT disorders, occasional rashes, nervousness, and drowsiness and in few cases there were hot flashes with increased appetite.

The overall analysis on primary outcomes revealed that supplements, SERMs, and hormones increased sperm concentration 6.26 [95% CI 3.32, 9.21], sperm motility 6.69 [95% CI 2.38, 10.99], and sperm morphology, respectively, superior to other included interventions and placebo. Similar findings were reported by Manish Kuchakulla et al. and Rossella Cannarella et al. that supplements improve fertility, sperm concentration and sperm motility but do not effects sperm morphology, which was also proved through multiple RCTs (Fallah et al., 2018; Cannarella et al., 2020; Kuchakulla et al., 2020). Specifically, zinc sulfate proved to be the best in increasing sperm concentration 11.49 [95% CI −6.42, 29.39] and sperm motility 16.78 [95% CI 14.27, 19.29] among all supplement interventions. This effect of zinc sulfate was due to its ability of increasing low and high molecular weight ligands in the semen (Ahmadi et al., 2016). Among all SERMS interventions, clomiphene citrate was the best in increasing sperm concentration 6.91 [95% CI 5.62, 8.20] and sperm motility 8.17 [95% CI 4.25, 12.10]. Clomiphene citrate exerts its effect through raising the endogenous serum FSH, LH and testosterone levels and initiating gametogenesis (Patankar et al., 2007). Meanwhile, testosterone was the best in increasing sperm morphology among all hormones 12.24 [95% CI 1.00, 23.49]. Studies suggested that increased serum testosterone levels lead to better morphology due to its role in pathogenesis of teratozoospermia (Tang et al., 2012).

In addition to primary outcomes, the results of NMA showed statistically noteworthy effect of supplements, hormones, SERMs, and vitamins on secondary outcomes as well. Total serum testosterone levels were significantly enhanced by supplements 2.70 [95% CI 1.34, 4.07] followed by SERMs 1.83 [95% CI 1.16, 2.50], hormones 0.40 [95% CI −0.49, 1.29], and vitamins −0.70 [95% CI -6.71, 5.31] in comparison to placebo. These are in agreement with previous reports (Thakur et al., 2015; Lo et al., 2018). CoQ10 2.77 [95% CI 1.83, 3.71] and tamoxifen citrate 1.50 [95% CI 1.20, 1.79] showed the best results in improving serum total testosterone levels among all supplements and SERMs, respectively (AinMelk et al., 1987; Krause et al., 1992; Safarinejad, 2009; Çakan et al., 2009; Safarinejad et al., 2012). CoQ10 supplementation was found to ameliorate the reduction in testosterone induced by chemicals mainly by neutralizing the generated free radicals (Banihani, 2018). However, tamoxifen citrate stimulated release of LH and FSH through negative feedback of estrogen at the hypothalamus and pituitary which in turn increases the testosterone biosynthesis and stimulates spermatogenesis (Rambhatla et al., 2016).

Serum FSH concentration was majorly increased by SERMs 3.63 [95% CI 1.48, 5.79] followed by hormones, vitamins, placebo, and supplements. Hormones also increase the serum FSH levels 1.29 [95% CI −0.79, 3.36] followed by a minor increase by vitamins 0.03 [95% CI −2.69, 2.76]. Supplements failed to increase serum FSH levels but rather decreased the serum FSH level significantly −4.45 [95% CI −7.15, −1.76] as compared to placebo. The reason involves understanding of the role of serum FSH in sperm production. FSH, along with testosterone, is necessary for maintaining normal sperm count and function in males. Normal spermatogenesis yields low levels of FSH whereas compromised spermatogenesis can yield high serum FSH levels. As discussed above, supplements are the best in improving sperm concentration through normal spermatogenesis resulting in decreased levels of serum FSH (Orlowski and Sarao, 2018). Tamoxifen citrate 3.66 [95% CI 1.27, 6.05] and FSH 2.74 [95% CI −0.00, 5.48] were the best in increasing the serum FSH levels among all SERMs and hormones, respectively (AinMelk et al., 1987; Krause et al., 1992; Kamischke et al., 1998; Çakan et al., 2009; Selice et al., 2011).

It is evident from the NMA of this review that concomitant use of supplements, SERMs, and hormones was associated with additional clinical benefits beyond sperm concentration, sperm motility, and sperm morphology and these include improvement in sex life and conception (Ring et al., 2016). Strict adherence to therapy along with healthy diet could be clinically significant in reducing male infertility and increasing the chances of conception among couples. Finding of our NMA shows that concomitant use of supplements, hormones, and SERMs irrespective of their types has shown significant increase in sperm concentration, sperm motility, sperm morphology, serum total testosterone, and serum FSH in males’ infertility. Furthermore, considering the type of treatment, combination of zinc sulfate, clomiphene citrate, and testosterone undecanoate can be used to increase the sperm parameters (sperm concentration, sperm motility, and sperm morphology) and combination of CoQ10, tamoxifen citrate, and FSH can be used to improve the hormonal profile in infertile males. The verdict of this NMA will enable policy makers to formulate or choose the different accessible interventions, keeping in view the anticipated advantageous outcomes and existing healthcare assets.

Among all RCTs included, the selection, detection, and performance bias were observed to be <35%. This could perhaps influence the NMA results; therefore, it is recommended to infer our NMA results with caution. Due to lack of methodical content elaboration and varied nature of interventions, it is very difficult to conclude which type of intervention will be the most effective. This is a common issue exclusive in complex interventions in which the description of methods is insufficient to extract data which contribute to success of the regimen. Publishing a separate protocol of study is recommended to empower the readers and investigators to better recognize and comprehend study element, enabling them to be replicated in future.

For better outcome reporting, additional investigation is desirable to evaluate the male infertility interventions with respect to time, frequency, and contents. In addition, this reading endows imperative insights for future research focusing on a couture intervention and economic cost investigation in delivering such interventions, and, hence, designing cost effective interventions. The outcomes of this read will assist policy makers in assortment of suitable interventions keeping in view the paramount utility of the available resources.

## Conclusion

It is observed that supplements appeared to be the best in increasing sperm concentration [6.26, 95% CI 3.32, 9.21; *p* = 0.00] and serum total testosterone levels [2.70, 95% CI 1.34, 4.07; *p* = 0.00]. On the other hand, hormones and SERMs intervention groups showed better sperm morphology [3.71, 95% CI 1.34, 6.07; *p* = 0.002), sperm motility [6.69, 95% CI 2.38, 10.99; *p* = 0.002], and serum FSH level [3.63, 95% CI 1.48, 5.79; *p* = 0.001], respectively.

## Clinical Implications

This is perhaps the first paper to compare the one-on-one comparison among the interventions and controls used to improve the sperm morphology and count. In addition, this paper has also compared the groupwise and overall effect of the intervention using network meta-analysis which will serve as an ideal approach to optimize the therapy based on the effect size and might be useful in optimizing the cost of therapy as well. This study is of significant value for healthcare providers and policy makers in selecting perfect blend of interventions for male infertility patients keeping in view of the existing health resources. This review establishes that all interventions had a significantly positive effect on male infertility (sperm count, sperm motility, sperm morphology, serum testosterone, and FSH). Statistically significant increased sperm parameters (sperm concentration, sperm motility, and sperm morphology) were noted in combinations of zinc sulfate (220 mg BID), clomiphene citrate (50 mg BID), and testosterone undecanoate and CoQ10; tamoxifen citrate and FSH were shown to improve the hormonal profile in infertile males. There is a need for the future experimental studies on these interventions with significant effect size so that a better pharmacotherapy can be planned to improve the outcome of therapy.

## Data Availability

The original contributions presented in the study are included in the article/Supplementary Material; further inquiries can be directed to the corresponding authors.
