# Supplementary material for: Effectiveness of Pharmacological Intervention Among Men with Infertility: A Systematic Review and Network Meta-Analysis
Source: Front Pharmacol. 2021 Aug 16;12:638628. doi: 10.3389/fphar.2021.638628 (PMC8415454; doi:10.3389/fphar.2021.638628)
Supplement: Supplementary file 1 [file DataSheet1.pdf]

Effectiveness of Pharmacological Intervention among Men with Infertility: A systematic review and network meta-analysis

Mr. Muhammad Nabeel Shahid* (Corresponding author and first author)

Affiliations:

1. Department of Pharmacy Practice, Faculty of Pharmacy, Universiti Teknologi MARA Cawangan Selangor, Puncak Alam Campus, Selangor Malaysia
2. Department of Pharmacy Practice, Institute of Pharmaceutical Sciences, University of Veterinary and Animal Sciences, 54000, Lahore, Punjab, Pakistan.

Email address:

nabeelshahidk@hotmail.com

Dr. Tahir Mehmood Khan* (Co-corresponding author and Author)

Affiliations:

1. Department of Pharmacy Practice, Institute of Pharmaceutical Sciences, University of Veterinary and Animal Sciences, 54000, Lahore, Punjab, Pakistan.

2. School of Pharmacy, Monash University, Jalan Lagoon Selatan 45700, Selangor, Malaysia.

Email address:

tahir.khan@uvas.edu.pk

Dr. Chin Fen Neoh (Author)

Affiliations:

1. Department of Pharmacy Practice, Faculty of Pharmacy, Universiti Teknologi MARA Cawangan Selangor, Puncak Alam Campus, Selangor Malaysia.

Email address:

chinfenneoh@gmail.com

Dr. Qi Ying Lean (Author)

Affiliations:

1. Faculty of Pharmacy, Universiti Teknologi MARA, Cawangan Pulau Pinang, Kampus Bertam, Pulau Pinang, Malaysia.
2. Vector-Borne Diseases Research Group (VERDI), Pharmaceutical abd Life Sciences CoRe, Universiti Teknologi MARA, Shah Alam, Selangor, Malaysia.

Email address:

leanqiying@yahoo.com

Dr. Allah Bukhsh (Author)

Affiliations:

1. Department of Pharmacy Practice, Institute of Pharmaceutical Sciences, University of Veterinary and Animal Sciences, 54000, Lahore, Pakistan.

2. School of Pharmacy, Monash University, Jalan Lagoon Selatan 45700, Selangor, Malaysia.

Email address:

abukhsh@uvas.edu.pk

Dr. Mahmathi Karuppannan (Author)

Affiliations:

1. Department of Pharmacy Practice, Faculty of Pharmacy, Universiti Teknologi MARA Cawangan Selangor, Puncak Alam Campus, Selangor Malaysia.

Email address:

mahmathi@uitm.edu.my

Supplementary material: Contents

Appendix 1 Search strategy

APPENDIX I: SEARCH STRATEGY

Search Term

Search Items		Search Engines: PubMed	Scopus	Cochrane Library	Embase	EBSCOhost	Ovid Medline	Google Scholar
("infertility"[MeSH Terms] OR "infertility"[All Fields]) AND ("oligospermia"[MeSH Terms] OR "oligospermia"[All Fields]) AND "humans"[MeSH Terms]	Result	5535	8200	207	4733	3049	4777	15
	Chosen	5499	4786	69	1810	403	4301	11
("infertility"[MeSH Terms] OR "infertility"[All Fields]) AND ("azoospermia"[MeSH Terms] OR "azoospermia"[All Fields] OR "azospermia"[All Fields]) AND "humans"[MeSH Terms]	Result	4709	352	18	6523	3705	8351	80
	Chosen	2352	173	14	2212	641	4375	25
("infertility"[MeSH Terms] OR "infertility"[All Fields]) AND ("oligospermia"[MeSH Terms] OR "oligospermia"[All Fields] OR "oligozoospermia"[All Fields]) AND "humans"[MeSH Terms]	Result	5992	5288	61	1494	1137	2672	15
	Chosen	236	2316	10	115	51	424	10
("infertility"[MeSH Terms] OR "infertility"[All Fields]) AND ("oligospermia"[MeSH Terms] OR "oligospermia"[All Fields] OR "oligoasthenoteratozoospermia"[All Fields]) AND "humans"[MeSH Terms]	Result	5677	1241	38	434	271	707	6
	Chosen	99	108	14	82	20	206	4
("infertility"[MeSH Terms] OR "infertility"[All Fields]) AND (("genitalia"[MeSH Terms] OR "genitalia"[All Fields] OR "genital"[All Fields]) AND ("disease"[MeSH Terms] OR "disease"[All Fields])) AND "humans"[MeSH Terms]	Result	3312	20352	108	22	1631	74	2
	Chosen	2859	16408	59	7	383	44	1
("infertility"[MeSH Terms] OR "infertility"[All Fields]) AND ("semen"[MeSH Terms] OR "semen"[All Fields]) AND "humans"[MeSH Terms]	Result	10225	41244	876	13428	11509	21294	373
	Chosen	7123	22701	322	4288	1307	12591	257
("infertility"[MeSH Terms] OR "infertility"[All Fields]) AND ("oligospermia"[MeSH Terms] OR "oligospermia"[All Fields] OR ("low"[All Fields] AND "sperm"[All Fields] AND "count"[All Fields]) OR "low sperm count"[All Fields]) AND "humans"[MeSH Terms]	Result	6036	7319	79	143	239	483	3
	Chosen	161	4161	20	33	1	181	2
("infertility"[MeSH Terms] OR "infertility"[All Fields] OR "subfertility"[All Fields]) AND ("oligospermia"[MeSH Terms] OR "oligospermia"[All Fields]) AND "humans"[MeSH Terms]	Result	5521	1224	29	318	135	402	0
	Chosen	0	26	2	28	1	23	0
("infertility"[MeSH Terms] OR "infertility"[All Fields] OR "subfertility"[All Fields]) AND ("azoospermia"[MeSH Terms] OR "azoospermia"[All Fields]) AND "humans"[MeSH Terms]	Result	4692	9	0	290	86	694	1
	Chosen	23	0	0	29	0	16	1
("infertility"[MeSH Terms] OR "infertility"[All Fields] OR "subfertility"[All Fields]) AND ("oligospermia"[MeSH Terms] OR "oligospermia"[All Fields] OR "oligozoospermia"[All Fields]) AND "humans"[MeSH Terms]	Result	5993	1119	29	93	52	333	3
	Chosen	1	7	0	4	3	2	2
("infertility"[MeSH Terms] OR "infertility"[All Fields] OR "subfertility"[All Fields]) AND ("oligospermia"[MeSH Terms] OR "oligospermia"[All Fields] OR "oligoasthenoteratozoospermia"[All Fields]) AND "humans"[MeSH Terms]	Result	5677	317	1	30	17	95	0
	Chosen	0	2	0	4	0	0	0

**("infertility"[MeSH Terms] OR "infertility"[All Fields] OR "subfertility"[All Fields]) AND (("genitalia"[MeSH Terms] OR "genitalia"[All Fields] OR "genital"[All Fields]) AND ("disease"[MeSH Terms] OR "disease"[All Fields]))**	Result	4162	1942	15	2	38	10	0
	Chosen	780	273	2	1	3	1	0
**("infertility"[MeSH Terms] OR "infertility"[All Fields] OR "subfertility"[All Fields]) AND ("semen"[MeSH Terms] OR "semen"[All Fields])**	Result	11781	6031	119	1110	508	2163	12
	Chosen	1552	463	19	199	17	218	9
**("infertility"[MeSH Terms] OR "infertility"[All Fields] OR "subfertility"[All Fields]) AND ("oligospermia"[MeSH Terms] OR "oligospermia"[All Fields] OR ("low"[All Fields] AND "sperm"[All Fields] AND "count"[All Fields]) OR "low sperm count"[All Fields])**	Result	6508	1465	16	9	5	76	0
	Chosen	321	76	3	3	0	0	0
**Subfertile[All Fields] AND ("oligospermia"[MeSH Terms] OR "oligospermia"[All Fields])**	Result	167	1449	29	242	169	377	0
	Chosen		49	0	20	4	59	0
**Subfertile[All Fields] AND ("azoospermia"[MeSH Terms] OR "azoospermia"[All Fields] OR "azospermia"[All Fields])**	Result	107	90	0	197	112	653	0
	Chosen	10	0	0	19	1	57	0
**Subfertile[All Fields] AND ("oligospermia"[MeSH Terms] OR "oligospermia"[All Fields] OR "oligozoospermia"[All Fields])**	Result	189	1364	11	85	67	345	0
	Chosen	1	23	0	4	0	10	0
**Subfertile[All Fields] AND ("oligospermia"[MeSH Terms] OR "oligospermia"[All Fields] OR "oligoasthenoteratozoospermia"[All Fields])**	Result	177	302	1	21	16	106	3
	Chosen	0	2	0	3	0	3	2
**Subfertile[All Fields] AND (("genitalia"[MeSH Terms] OR "genitalia"[All Fields] OR "genital"[All Fields]) AND ("disease"[MeSH Terms] OR "disease"[All Fields]))**	Result	65	1690	15	0	27	6	0
	Chosen	7	268	0	0	0	1	0
**Subfertile[All Fields] AND ("semen"[MeSH Terms] OR "semen"[All Fields])**	Result	747	6861	119	5078	781	2229	82
	Chosen	131	872	1	120	28	346	60
**Subfertile[All Fields] AND ("oligospermia"[MeSH Terms] OR "oligospermia"[All Fields] OR ("low"[All Fields] AND "sperm"[All Fields] AND "count"[All Fields]) OR "low sperm count"[All Fields])**	Result	224	1339	17	11	16	76	0
	Chosen	0	78	0	0	1	2	0

Supplementary Table 1: Reasons for study exclusion after full-text assessment (n=607)

**Database**	**Number of records**
Review articles	**203**
Irrelevent articles (conference papers/proceedings, letter to the editor and study protocols)	**194**
Meta-Analysis	**19**
Surgical intervention articles	**30**
Systematic reviews	**44**
Does not meet selection criteria (details are as under)-	**75**
Not measured male patient data	*37*
Not measured the selected outcomes	*51*

Supplementary Figure 1: Overall meta-analysis for sperm concentration of included RCTs (n=29)

Study or Subgroup	Intervention Mean	Intervention SD	Intervention Total	Placebo Mean	Placebo SD	Placebo Total	Weight	Mean Difference IV, Random, 95% CI	Risk of Bias A	B	C	D	E	F	G
10 Mićić 1985	7.62	3.62	56	0.72	3.18	45	4.1%	6.90 [5.57, 8.23]	?	?	?	-	-	-	+
14 AinMelk 1987	0.68	24.33	16	1.4	24.24	16	0.6%	-0.72 [-17.55, 16.11]	+	+	?	+	+	+	+
19a A. Nadjarzadeh 2012	17.47	13.66	23	16.21	14.96	24	1.7%	1.26 [-6.92, 9.44]	+	+	?	+	+	+	+
22 Abdul Razzaq 2018	23	18.73	60	0	21	60	2.0%	23.00 [15.88, 30.12]	-	-	?	+	?	?	?
22a Nicola Colacurci 2012	3.8	3.84	65	-0.2	3.47	64	4.1%	4.00 [2.74, 5.26]	?	?	-	-	-	-	-
23a Martin Imhof 2012	13.5	15.21	132	2.6	5.85	73	3.6%	10.90 [7.98, 13.82]	-	-	+	-	-	-	-
24a Mohammad Reza Safarinejad 2012	28.7	4.6	101	17	4	102	4.1%	11.70 [10.51, 12.89]	+	+	?	+	+	+	+
25 Pusch 1988	6.75	14.56	29	6.3	19.91	28	1.5%	0.45 [-8.63, 9.53]	+	+	?	+	+	+	?
26 Mahmoud Hussein 2012	2	17	37	0	17	37	1.8%	2.00 [-5.75, 9.75]	-	-	?	+	?	?	?
26a A. Nadjarzadeh 2011	-0.04	11.95	23	-3.57	11.98	24	2.1%	3.53 [-3.31, 10.37]	+	+	+	+	+	+	+
31a Hussein Ghanem 2009	7.8	13.42	30	0.7	7.97	30	2.5%	7.10 [1.51, 12.69]	+	+	+	?	+	+	-
33a Murat 2008	5.1	28.1	42	0.1	13.42	25	1.3%	5.00 [-4.99, 14.99]	+	?	-	+	+	-	-
33a Murat 2008	7.6	27.65	31	0.1	13.42	25	1.1%	7.50 [-3.56, 18.56]	+	?	-	+	+	-	-
33a Murat 2008	-0.4	18.34	30	0.1	13.42	25	1.6%	-0.50 [-8.91, 7.91]	+	?	-	+	+	-	-
34a Mohammad Reza Safarinejad 2009	6.2	4.5	98	0.4	4.35	96	4.1%	5.80 [4.55, 7.05]	-	-	-	-	-	-	-
38a RobertoParadisi 2006	8.5	9.81	15	0.1	4.37	15	2.5%	8.40 [2.97, 13.83]	+	+	?	?	+	+	+
41a Andrea Ienzi 2004	4.02	7.92	30	6.1	14.74	26	2.2%	-2.08 [-8.42, 4.26]	+	+	+	-	+	+	+
44a Ettore Caroppo 2003	2.5	5.73	23	0.11	2.15	10	3.7%	2.39 [-0.30, 5.08]	?	?	-	?	-	-	-
45a Dimitrios A 2003	34.4	205.95	106	6.3	179.31	106	0.1%	28.10 [-23.88, 80.08]	-	?	?	-	-	-	-
46a Wai Yee Wong 2002	4	140.73	22	1.5	122.11	25	0.0%	2.50 [-73.32, 78.32]	+	+	?	+	+	+	-
46a Wai Yee Wong 2002	4.5	190.81	24	1.5	122.11	25	0.0%	3.00 [-87.10, 93.10]	+	+	?	+	+	+	-
46a Wai Yee Wong 2002	4.5	85.59	23	1.5	122.11	25	0.1%	3.00 [-56.28, 62.28]	+	+	?	+	+	+	-
47a Carlo Foresta 2002	4.5	3.14	15	1.3	2.46	15	3.9%	3.20 [1.18, 5.22]	+	+	-	?	+	-	-
47a Carlo Foresta 2002	2.1	2.31	15	1.3	2.46	15	4.0%	0.80 [-0.91, 2.51]	+	+	-	?	+	-	-
50a A.Kamischke 1998	1.2	10.92	34	2.5	10.58	31	2.6%	-1.30 [-6.53, 3.93]	+	+	?	?	+	+	+
59a C.Keck 1994	24	94.4	44	22	39.37	47	0.2%	2.00 [-28.08, 32.08]	+	+	?	?	+	+	-
63 a W. KRAUSE 1992	2.1	12.82	39	0.2	8.09	37	2.8%	1.90 [-2.89, 6.69]	+	-	-	?	-	-	-
67a Yin-man Ding 2015	1.7	6.85	38	0.9	2.93	30	3.8%	0.80 [-1.62, 3.22]	?	?	+	+	+	+	?
67a Yin-man Ding 2015	1.6	3.38	36	0.9	2.93	30	4.1%	0.70 [-0.82, 2.22]	?	?	+	+	+	+	?
67a Yin-man Ding 2015	14	9.01	40	0.9	2.93	30	3.5%	13.10 [10.12, 16.08]	?	?	+	+	+	+	?
67a Yin-man Ding 2015	10.1	6.44	41	0.9	2.93	30	3.8%	9.20 [6.97, 11.43]	?	?	+	+	+	+	?
72a Milat 2015	3.58	2.21	34	-2.4	2.72	29	4.1%	5.98 [4.74, 7.22]	+	?	-	?	+	-	?
72a Milat 2015	1.75	2.04	20	-2.4	2.72	29	4.1%	4.15 [2.82, 5.48]	+	?	-	?	+	-	?
72a Milat 2015	3.23	2.57	45	-2.4	2.72	29	4.1%	5.63 [4.39, 6.87]	+	?	-	?	+	-	?
74a Markus Lipovac 2016	5.7	20.48	156	7.9	18.4	143	2.9%	-2.20 [-6.61, 2.21]	?	?	?	?	?	?	?
76a A. FARRAG 2015	3.74	6.89	36	0	3.1	46	3.8%	3.74 [1.32, 6.16]	?	?	-	?	-	-	+
77a R. Selice 2010	8.6	11.3	70	4.1	4.2	35	3.5%	4.50 [1.51, 7.49]	-	?	-	?	+	?	-
78a R. Paradisi 2013	13.4	7.5	45	7.2	4.2	15	3.5%	6.20 [3.15, 9.25]	?	?	?	?	-	-	-
80a TinaKoldJensen 2020	45	62.42	25	42	427.12	1125	0.1%	3.00 [-31.95, 37.95]	-	-	?	?	-	-	-
80a TinaKoldJensen 2020	38	112.44	75	42	427.12	1125	0.1%	-4.00 [-39.64, 31.64]	-	-	?	?	-	-	-
80a TinaKoldJensen 2020	46	160.66	98	42	427.12	1125	0.1%	4.00 [-36.43, 44.43]	-	-	?	?	-	-	-
80a TinaKoldJensen 2020	43	242.43	210	42	427.12	1125	0.1%	1.00 [-40.21, 42.21]	-	-	?	?	-	-	-
**Total (95% CI)**			**2132**			**5997**	**100.0%**	**4.90 [3.54, 6.26]**							

Heterogeneity: Tau² = 11.31; Chi² = 320.09, df = 41 (P < 0.00001); I² = 87%

Test for overall effect: Z = 7.05 (P < 0.00001)

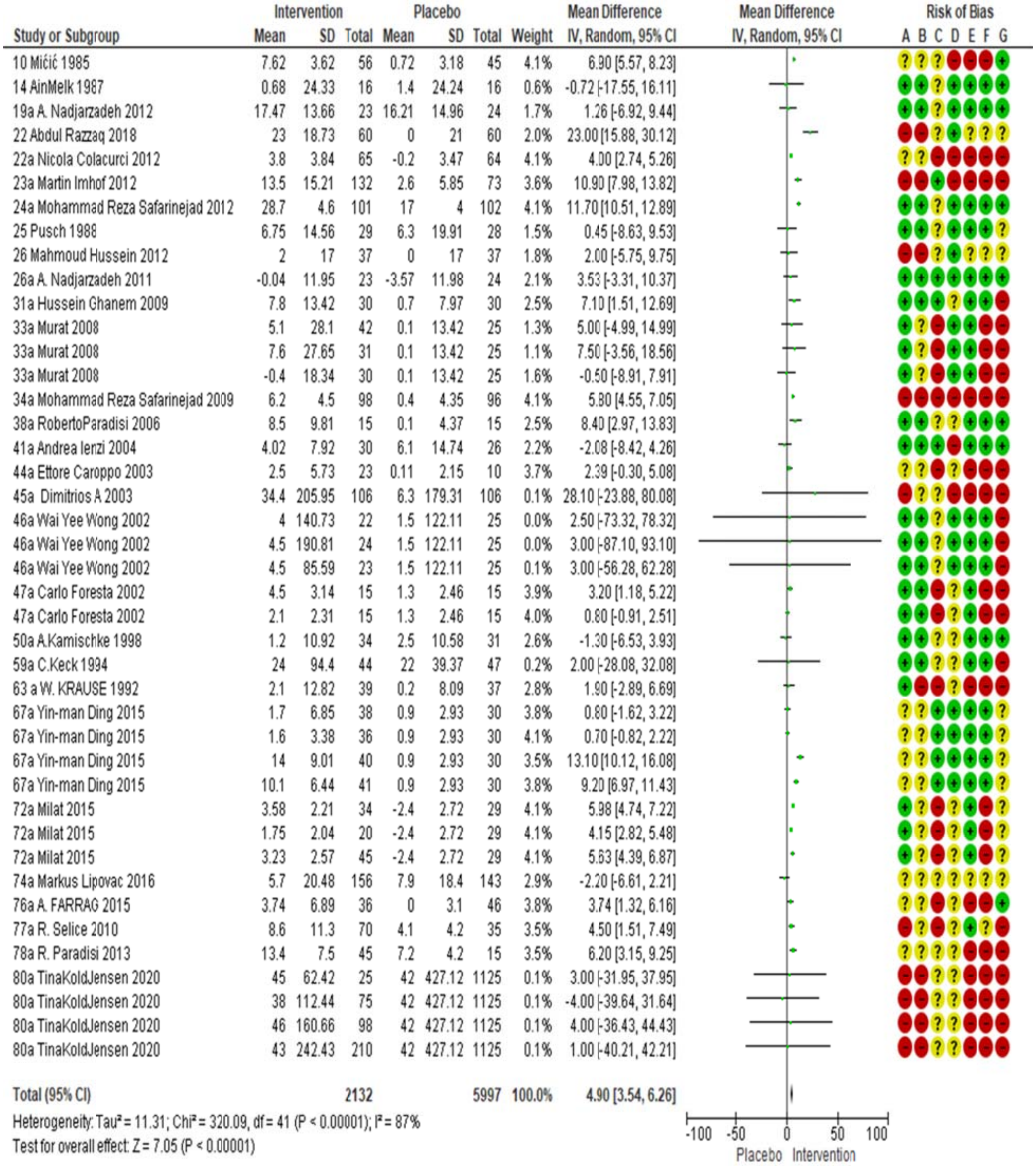

Risk of bias legend

(A) Random sequence generation (selection bias)

(B) Allocation concealment (selection bias)

(C) Selective reporting (reporting bias)

(D) Other bias

(E) Blinding of participants and personnel (performance bias)

(F) Blinding of outcome assessment (detection bias)

(G) Incomplete outcome data (attrition bias)

Supplementary Figure 2: Overall meta-analysis for sperm motility of included RCTs (n=29)

Study or Subgroup	Intervention Mean	Intervention SD	Intervention Total	Placebo Mean	Placebo SD	Placebo Total	Weight	Mean Difference IV, Random, 95% CI
10 Mićić 1985	6.24	11.85	56	-2.18	10.39	46	3.2%	8.42 [4.10, 12.74]
14 AinMelk 1987	-0.84	36.29	16	2.08	39.92	16	0.4%	-2.92 [-29.35, 23.51]
19a A. Nadjarzadeh 2012	5.78	16.88	23	0.54	18.46	24	1.7%	5.24 [-4.87, 15.35]
22 Abdul Razzaq 2018	18	12.29	60	0	9	60	3.3%	18.00 [14.15, 21.85]
22a Nicola Colacurci 2012	2.5	5.1	65	0.6	4.91	64	3.8%	1.90 [0.17, 3.63]
23a Martin Imhof 2012	16.5	18.89	132	9.5	34.3	73	2.0%	7.00 [-1.50, 15.50]
24a Mohammad Reza Safarinejad 2012	35.8	2.7	101	25	2	102	3.9%	10.80 [10.15, 11.45]
25 Pusch 1988	2.8	10.04	29	1.4	12.54	28	2.7%	1.40 [-4.51, 7.31]
26 Mahmoud Hussein 2012	16	7.55	37	0	7	37	3.4%	16.00 [12.68, 19.32]
26a A. Nadjarzadeh 2011	5.78	16.87	23	0.51	17.29	24	1.8%	5.27 [-4.50, 15.04]
31a Hussein Ghanem 2009	1	20.07	30	-6	17.09	30	1.8%	7.00 [-2.43, 16.43]
33a Murat 2008	13.12	17.1	31	-3.02	14.4	25	2.1%	16.14 [7.89, 24.39]
33a Murat 2008	10.52	18.12	42	-3.02	14.4	25	2.2%	13.54 [5.67, 21.41]
33a Murat 2008	-5.08	13.02	30	-3.02	14.4	25	2.3%	-2.06 [-9.38, 5.26]
34a Mohammad Reza Safarinejad 2009	5.4	2.31	98	0.8	2.39	96	3.9%	4.60 [3.94, 5.26]
38a RobertoParadisi 2006	1.9	5.45	15	0.6	5.55	15	3.3%	1.30 [-2.64, 5.24]
41a Andrea Ierzi 2004	7.94	11.66	30	6.47	8.41	26	2.9%	1.47 [-3.81, 6.75]
44a Ettore Caroppo 2003	-3.82	42.7	23	-8.99	39.33	10	0.3%	5.17 [-24.81, 35.15]
45a Dimitrios A 2003	11.9	12.59	106	1.1	15.5	106	3.3%	10.80 [7.00, 14.60]
46a Wai Yee Wong 2002	5	69.12	22	0	89.59	25	0.1%	5.00 [-40.47, 50.47]
46a Wai Yee Wong 2002	5	80.55	23	0	89.59	25	0.1%	5.00 [-43.14, 53.14]
46a Wai Yee Wong 2002	2	86.28	24	0	89.59	25	0.1%	2.00 [-47.24, 51.24]
47a Carlo Foresta 2002	1.8	8.74	15	-1	7.76	15	2.7%	2.80 [-3.11, 8.71]
47a Carlo Foresta 2002	1.7	10.48	15	-1	7.76	15	2.5%	2.70 [-3.90, 9.30]
50a A.Kamischke 1998	-2.9	16.94	34	5.8	16.7	31	2.1%	-8.70 [-16.88, -0.52]
59a C.Keck 1994	3.4	22.47	44	1.7	20.97	47	1.9%	1.70 [-7.25, 10.65]
63 a W. KRAUSE 1992	-1.3	25.44	39	-3.1	19.25	37	1.7%	1.80 [-8.31, 11.91]
67a Yin-man Ding 2015	2.3	4.91	36	1.3	8	30	3.4%	1.00 [-2.28, 4.28]
67a Yin-man Ding 2015	6	11.14	40	1.3	8	30	3.1%	4.70 [0.22, 9.18]
67a Yin-man Ding 2015	6.2	8.98	41	1.3	8	30	3.3%	4.90 [0.93, 8.87]
67a Yin-man Ding 2015	3.9	11.25	38	1.3	8	30	3.1%	2.60 [-1.98, 7.18]
72a Milat 2015	5.76	3.4	34	-1.5	4.91	29	3.7%	7.26 [5.14, 9.38]
72a Milat 2015	2.33	3.69	45	-1.5	4.91	29	3.7%	3.83 [1.74, 5.92]
72a Milat 2015	1.63	3.16	20	-1.5	4.91	29	3.7%	3.13 [0.87, 5.39]
74a Markus Lipovac 2016	9.2	18.2	156	15.2	16.31	143	3.3%	-6.00 [-9.91, -2.09]
76a A. FARRAG 2015	16	12.19	36	0	7.39	46	3.1%	16.00 [11.48, 20.52]
77a R. Selice 2010	4.7	16.67	70	1.5	16.61	35	2.5%	3.20 [-3.55, 9.95]
78a R. Paradisi 2013	0.4	5.76	45	-0.1	5.31	15	3.5%	0.50 [-2.67, 3.67]
80a TinaKoldJensen 2020	60	77.07	210	62.7	175.87	1125	1.1%	-2.70 [-17.34, 11.94]
80a TinaKoldJensen 2020	63.2	53.39	98	62.7	175.87	1125	1.0%	0.50 [-14.24, 15.24]
80a TinaKoldJensen 2020	63.6	35.58	25	62.7	175.87	1125	0.8%	0.90 [-16.42, 18.22]
80a TinaKoldJensen 2020	60.3	51.46	75	62.7	175.87	1125	1.0%	-2.40 [-17.93, 13.13]
**Total (95% CI)**			**2132**			**5998**	**100.0%**	**4.95 [3.19, 6.72]**

Heterogeneity: $Tau^2 = 20.71$; $Chi^2 = 444.22$, df = 41 ($P < 0.00001$); $I^2 = 91\%$

Test for overall effect: Z = 5.51 ($P < 0.00001$)

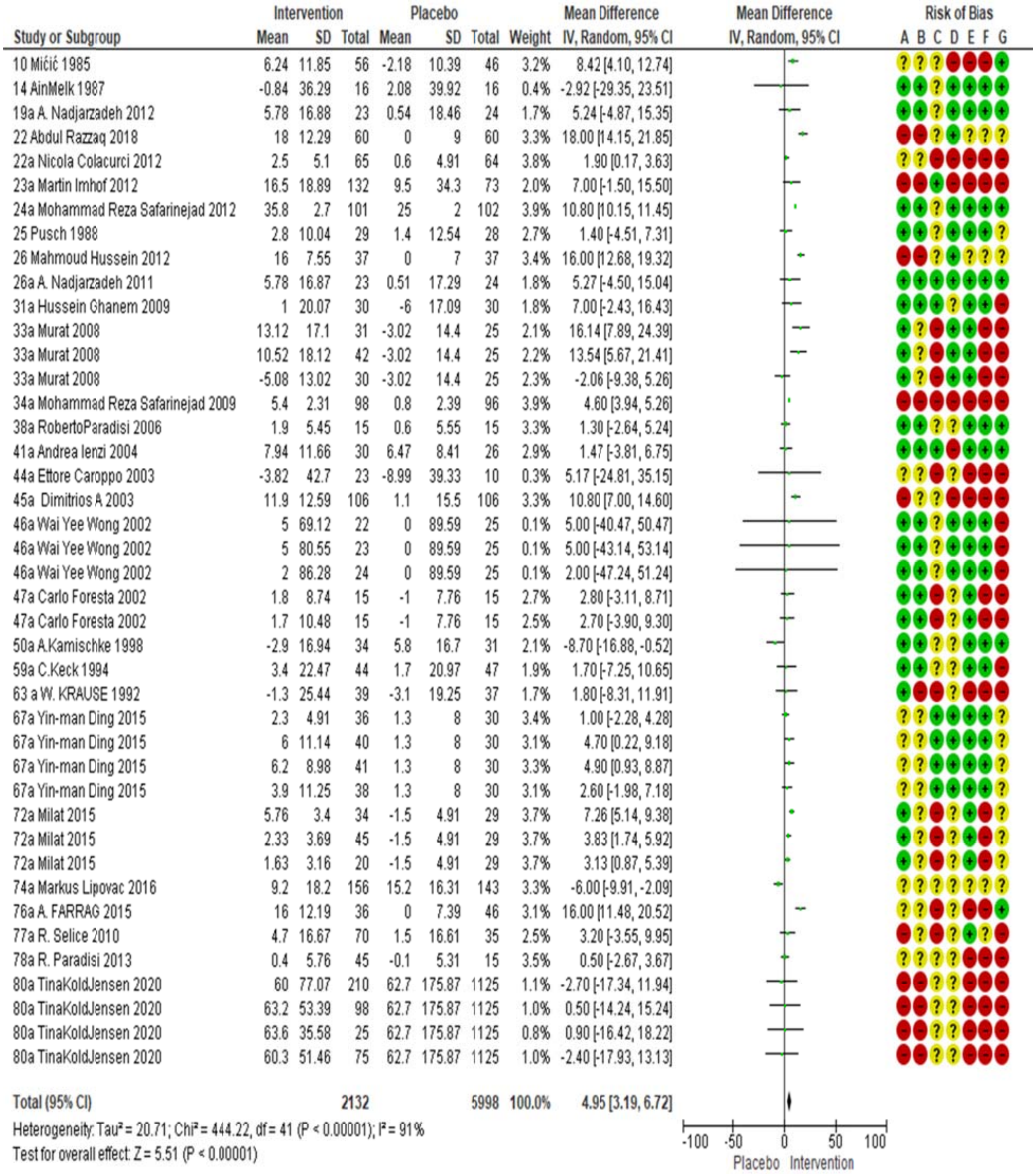

Risk of bias legend

(A) Random sequence generation (selection bias)

(B) Allocation concealment (selection bias)

(C) Selective reporting (reporting bias)

(D) Other bias

(E) Blinding of participants and personnel (performance bias)

(F) Blinding of outcome assessment (detection bias)

(G) Incomplete outcome data (attrition bias)

Supplementary Figure 3: Overall meta-analysis for sperm morphology of included RCTs (n=29)

Study or Subgroup	Intervention Mean	Intervention SD	Intervention Total	Placebo Mean	Placebo SD	Placebo Total	Weight	Mean Difference IV, Random, 95% CI	Risk of Bias A	B	C	D	E	F	G
10 Mićić 1985	5.6	15.68	56	0.98	14.27	45	1.2%	4.62 [-1.23, 10.47]	?	?	?	-	-	-	+
14 AinMelk 1987	-3.75	68.51	16	-2.92	70.05	16	0.0%	-0.83 [-48.84, 47.18]	+	+	?	+	+	+	+
19a A. Nadjarzadeh 2012	-0.91	4.81	23	0.21	4.92	24	3.0%	-1.12 [-3.90, 1.66]	+	+	?	+	+	+	+
22 Abdul Razzaq 2018	12	9.64	60	0	11	60	2.2%	12.00 [8.30, 15.70]	-	-	?	+	?	?	?
22a Nicola Colacurci 2012	1.3	7.11	65	-0.1	7.52	64	3.3%	1.40 [-1.13, 3.93]	?	?	-	-	-	-	-
23a Martin Imhof 2012	11	12.16	132	-3.5	30.02	73	0.8%	14.50 [7.31, 21.69]	-	-	+	-	-	-	-
24a Mohammad Reza Safarinejad 2012	17.6	4.4	101	14.8	4.1	102	4.8%	2.80 [1.63, 3.97]	+	+	?	+	+	+	+
25 Pusch 1988	6.86	15.49	29	1	16.61	28	0.7%	5.86 [-2.48, 14.20]	+	+	?	+	+	+	?
26 Mahmoud Hussein 2012	5	10.58	37	0	12	37	1.4%	5.00 [-0.15, 10.15]	-	-	?	+	?	?	?
26a A. Nadjarzadeh 2011	-0.91	5.1	23	0.21	5.08	24	2.9%	-1.12 [-4.03, 1.79]	+	+	+	+	+	+	+
31a Hussein Ghanem 2009	3	17.09	30	-10	14.53	30	0.7%	13.00 [4.97, 21.03]	+	+	+	?	+	+	-
33a Murat 2008	-0.08	0.6	42	0.02	0.56	25	5.5%	-0.10 [-0.38, 0.18]	+	?	-	+	+	-	-
33a Murat 2008	-0.08	0.6	31	0.02	0.56	25	5.5%	-0.10 [-0.40, 0.20]	+	?	-	+	+	-	-
33a Murat 2008	-0.08	0.6	30	0.02	0.56	25	5.5%	-0.10 [-0.41, 0.21]	+	?	-	+	+	-	-
34a Mohammad Reza Safarinejad 2009	2.4	2.51	98	0.5	2.46	96	5.2%	1.90 [1.20, 2.60]	-	-	-	-	-	-	-
38a RobertoParadisi 2006	0.9	8.7	15	-1.7	8.78	15	1.1%	2.60 [-3.66, 8.86]	+	+	?	?	+	+	+
41a Andrea lenzi 2004	1.28	2.41	30	4.3	3.2	26	4.4%	-3.02 [-4.52, -1.52]	+	+	+	-	+	+	+
44a Ettore Caroppo 2003	5.2	8.83	23	-2.7	12.24	10	0.6%	7.90 [-0.50, 16.30]	?	?	-	?	-	-	-
45a Dimitrios A 2003	15.4	12.93	106	-2	15.89	106	2.1%	17.40 [13.50, 21.30]	-	?	?	-	-	-	-
46a Wai Yee Wong 2002	-1	7.28	23	0	9.42	25	1.6%	-1.00 [-5.74, 3.74]	+	+	?	+	+	+	-
46a Wai Yee Wong 2002	0	11.29	22	0	9.42	25	1.1%	0.00 [-5.99, 5.99]	+	+	?	+	+	+	-
46a Wai Yee Wong 2002	0	14.1	24	0	9.42	25	0.9%	0.00 [-6.74, 6.74]	+	+	?	+	+	+	-
47a Carlo Foresta 2002	-0.7	9.72	15	1.2	9.64	15	0.9%	-1.90 [-8.83, 5.03]	+	+	-	?	+	-	-
47a Carlo Foresta 2002	5.4	8.58	15	1.2	9.64	15	1.0%	4.20 [-2.33, 10.73]	+	+	-	?	+	-	-
50a A.Kamischke 1998	0.2	7.06	34	-0.2	6.98	31	2.5%	0.40 [-3.02, 3.82]	+	+	?	?	+	+	+
59a C.Keck 1994	16	24.84	44	13	32.52	47	0.3%	3.00 [-8.85, 14.85]	+	+	?	?	+	+	-
63 a W. KRAUSE 1992	-0.3	21.38	39	-5.2	18.03	37	0.6%	4.90 [-3.98, 13.78]	+	-	-	?	-	-	-
67a Yin-man Ding 2015	6.4	8.51	38	2.2	9.85	30	1.8%	4.20 [-0.24, 8.64]	?	?	+	+	+	+	?
67a Yin-man Ding 2015	-2.5	3.48	36	2.2	9.85	30	2.2%	-4.70 [-8.40, -1.00]	?	?	+	+	+	+	?
67a Yin-man Ding 2015	4.4	11.43	40	2.2	9.85	30	1.5%	2.20 [-2.80, 7.20]	?	?	+	+	+	+	?
67a Yin-man Ding 2015	6.1	11.43	41	2.2	9.85	30	1.5%	3.90 [-1.07, 8.87]	?	?	+	+	+	+	?
72a Milat 2015	0.56	0.71	45	-1	1.2	29	5.4%	1.56 [1.08, 2.04]	+	?	-	?	+	-	?
72a Milat 2015	1.12	0.62	34	-1	1.2	29	5.4%	2.12 [1.64, 2.60]	+	?	-	?	+	-	?
72a Milat 2015	2.01	0.23	20	-1	1.2	29	5.4%	3.01 [2.56, 3.46]	+	?	-	?	+	-	?
74a Markus Lipovac 2016	9.6	15.16	156	9.7	15.86	143	2.4%	-0.10 [-3.62, 3.42]	?	?	?	?	?	?	?
76a A. FARRAG 2015	6.36	8.34	36	0	7	46	2.5%	6.36 [2.97, 9.75]	?	?	-	?	-	-	+
77a R. Selice 2010	2.2	7.76	70	-1.4	6.21	35	3.1%	3.60 [0.85, 6.35]	-	?	-	?	+	?	-
78a R. Paradisi 2013	0.2	7.45	45	-2.6	8.78	15	1.5%	2.80 [-2.15, 7.75]	?	?	?	?	-	-	-
80a TinaKoldJensen 2020	6	11.02	75	6.5	50.24	1125	2.1%	-0.50 [-4.35, 3.35]	-	-	?	?	-	-	-
80a TinaKoldJensen 2020	6.8	27.13	210	6.5	50.24	1125	1.6%	0.30 [-4.40, 5.00]	-	-	?	?	-	-	-
80a TinaKoldJensen 2020	7.5	13.34	98	6.5	50.24	1125	2.1%	1.00 [-2.95, 4.95]	-	-	?	?	-	-	-
80a TinaKoldJensen 2020	6.5	10.36	25	6.5	50.24	1125	1.5%	0.00 [-5.01, 5.01]	-	-	?	?	-	-	-
**Total (95% CI)**			**2132**			**5997**	**100.0%**	**1.96 [1.24, 2.68]**							

Heterogeneity: Tau² = 2.45; Chi² = 451.40, df = 41 (P < 0.00001); I² = 91%

Test for overall effect: Z = 5.33 (P < 0.00001)

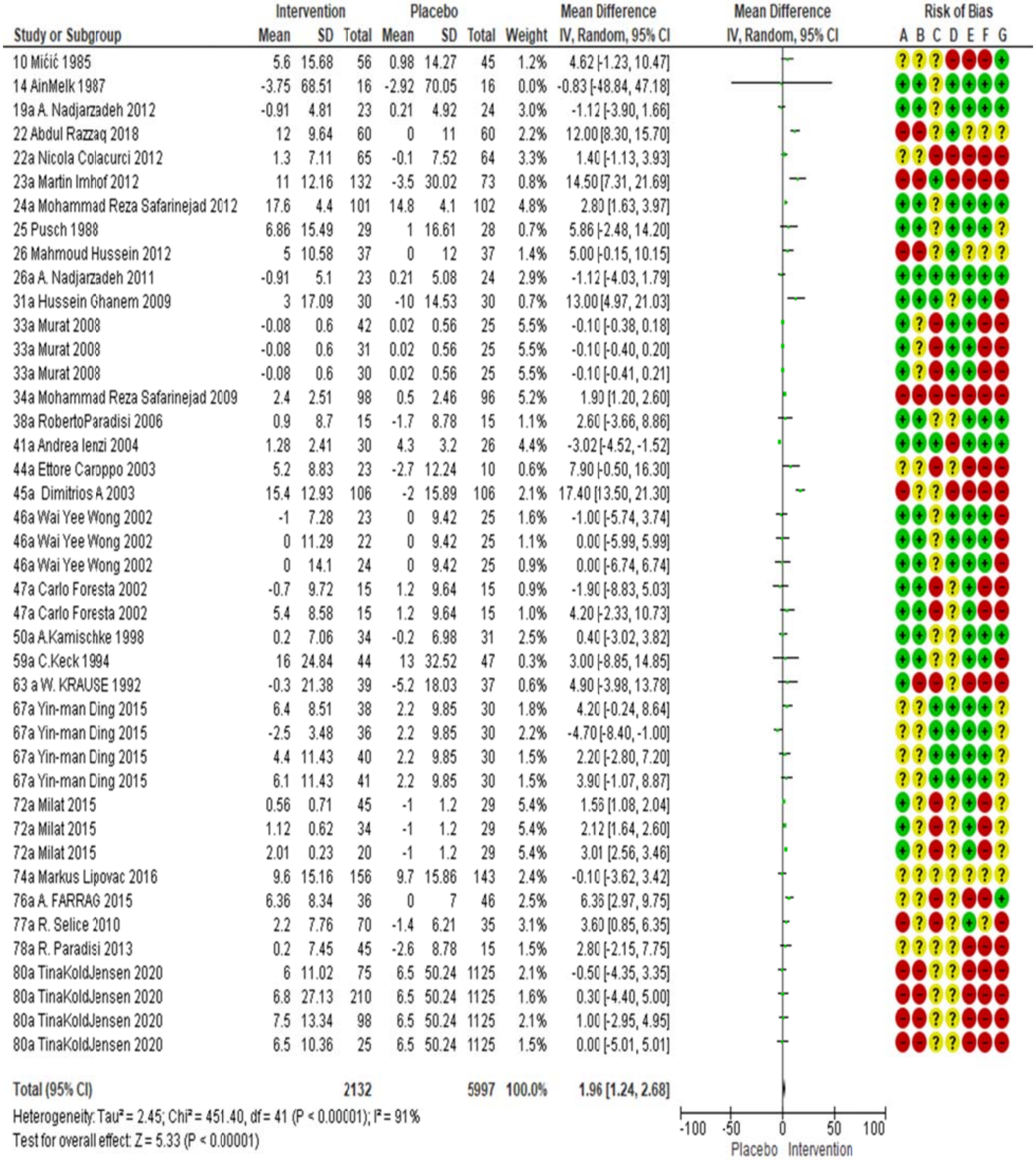

Risk of bias legend

(A) Random sequence generation (selection bias)

(B) Allocation concealment (selection bias)

(C) Selective reporting (reporting bias)

(D) Other bias

(E) Blinding of participants and personnel (performance bias)

(F) Blinding of outcome assessment (detection bias)

(G) Incomplete outcome data (attrition bias)

Supplementary Figure 4: Sub-group meta-analysis (intervention based) for sperm concentration (n=29)

Study or Subgroup	Intervention Mean	Intervention SD	Intervention Total	Placebo Mean	Placebo SD	Placebo Total	Weight	Mean Difference IV, Random, 95% CI	Year
**1.3.2 SERM**									
10 Mićić 1985	7.62	3.62	56	0.72	3.18	45	4.1%	6.90 [5.57, 8.23]	1985
14 AinMelk 1987	0.68	24.33	16	1.4	24.24	16	0.6%	-0.72 [-17.55, 16.11]	1987
63 a W. KRAUSE 1992	2.1	12.82	39	0.2	8.09	37	2.8%	1.90 [-2.89, 6.69]	1992
33a Murat 2008	7.6	27.65	31	0.1	13.42	25	1.1%	7.50 [-3.56, 18.56]	2008
33a Murat 2008	-0.4	18.34	30	0.1	13.42	25	1.6%	-0.50 [-8.91, 7.91]	2008
33a Murat 2008	5.1	28.1	42	0.1	13.42	25	1.3%	5.00 [-4.99, 14.99]	2008
31a Hussein Ghanem 2009	7.8	13.42	30	0.7	7.97	30	2.5%	7.10 [1.51, 12.69]	2009
72a Milat 2015	3.23	2.57	45	-2.4	2.72	29	4.1%	5.63 [4.39, 6.87]	2015
72a Milat 2015	3.58	2.21	34	-2.4	2.72	29	4.1%	5.98 [4.74, 7.22]	2015
**Subtotal (95% CI)**			**323**			**261**	**22.2%**	**6.00 [5.27, 6.72]**	
Heterogeneity: $Tau^2 = 0.02$; $Chi^2 = 8.08$, df = 8 (P = 0.43); $I^2 = 1\%$									
Test for overall effect: Z = 16.26 (P < 0.00001)									
**1.3.3 Hormones**									
25 Pusch 1988	6.75	14.56	29	6.3	19.91	28	1.5%	0.45 [-8.63, 9.53]	1988
50a A.Kamischke 1998	1.2	10.92	34	2.5	10.58	31	2.6%	-1.30 [-6.53, 3.93]	1998
47a Carlo Foresta 2002	4.5	3.14	15	1.3	2.46	15	3.9%	3.20 [1.18, 5.22]	2002
47a Carlo Foresta 2002	2.1	2.31	15	1.3	2.46	15	4.0%	0.80 [-0.91, 2.51]	2002
44a Ettore Caroppo 2003	2.5	5.73	23	0.11	2.15	10	3.7%	2.39 [-0.30, 5.08]	2003
45a Dimitrios A 2003	34.4	205.95	106	6.3	179.31	106	0.1%	28.10 [-23.88, 80.08]	2003
38a RobertoParadisi 2006	8.5	9.81	15	0.1	4.37	15	2.5%	8.40 [2.97, 13.83]	2006
77a R. Selice 2010	8.6	11.3	70	4.1	4.2	35	3.5%	4.50 [1.51, 7.49]	2010
22a Nicola Colacurci 2012	3.8	3.84	65	-0.2	3.47	64	4.1%	4.00 [2.74, 5.26]	2012
78a R. Paradisi 2013	13.4	7.5	45	7.2	4.2	15	3.5%	6.20 [3.15, 9.25]	2013
67a Yin-man Ding 2015	1.7	6.85	38	0.9	2.93	30	3.8%	0.80 [-1.62, 3.22]	2015
67a Yin-man Ding 2015	14	9.01	40	0.9	2.93	30	3.5%	13.10 [10.12, 16.08]	2015
67a Yin-man Ding 2015	10.1	6.44	41	0.9	2.93	30	3.8%	9.20 [6.97, 11.43]	2015
67a Yin-man Ding 2015	1.6	3.38	36	0.9	2.93	30	4.1%	0.70 [-0.82, 2.22]	2015
76a A. FARRAG 2015	3.74	6.89	36	0	3.1	46	3.8%	3.74 [1.32, 6.16]	2015
**Subtotal (95% CI)**			**608**			**500**	**48.4%**	**4.14 [2.30, 5.99]**	
Heterogeneity: $Tau^2 = 9.80$; $Chi^2 = 104.67$, df = 14 (P < 0.00001); $I^2 = 87\%$									
Test for overall effect: Z = 4.40 (P < 0.0001)									
**1.3.6 Vitamins**									
46a Wai Yee Wong 2002	4	140.73	22	1.5	122.11	25	0.0%	2.50 [-73.32, 78.32]	2002
80a TinaKoldJensen 2020	45	62.42	25	42	427.12	1125	0.1%	3.00 [-31.95, 37.95]	2020
80a TinaKoldJensen 2020	43	242.43	210	42	427.12	1125	0.1%	1.00 [-40.21, 42.21]	2020
80a TinaKoldJensen 2020	38	112.44	75	42	427.12	1125	0.1%	-4.00 [-39.64, 31.64]	2020
**Subtotal (95% CI)**			**332**			**3400**	**0.4%**	**0.14 [-20.41, 20.69]**	
Heterogeneity: $Tau^2 = 0.00$; $Chi^2 = 0.08$, df = 3 (P = 0.99); $I^2 = 0\%$									
Test for overall effect: Z = 0.01 (P = 0.99)									
**1.3.7 Enzymes**									
59a C.Keck 1994	24	94.4	44	22	39.37	47	0.2%	2.00 [-28.08, 32.08]	1994
**Subtotal (95% CI)**			**44**			**47**	**0.2%**	**2.00 [-28.08, 32.08]**	
Heterogeneity: Not applicable									
Test for overall effect: Z = 0.13 (P = 0.90)									
**1.3.8 Supplements**									
46a Wai Yee Wong 2002	4.5	190.81	24	1.5	122.11	25	0.0%	3.00 [-87.10, 93.10]	2002
46a Wai Yee Wong 2002	4.5	85.59	23	1.5	122.11	25	0.1%	3.00 [-56.28, 62.28]	2002
41a Andrea lenzi 2004	4.02	7.92	30	6.1	14.74	26	2.2%	-2.08 [-8.42, 4.26]	2004
34a Mohammad Reza Safarinejad 2009	6.2	4.5	98	0.4	4.35	96	4.1%	5.80 [4.55, 7.05]	2009
26a A. Nadjarzadeh 2011	-0.04	11.95	23	-3.57	11.98	24	2.1%	3.53 [-3.31, 10.37]	2011
24a Mohammad Reza Safarinejad 2012	28.7	4.6	101	17	4	102	4.1%	11.70 [10.51, 12.89]	2012
23a Martin Imhof 2012	13.5	15.21	132	2.6	5.85	73	3.6%	10.90 [7.98, 13.82]	2012
19a A. Nadjarzadeh 2012	17.47	13.66	23	16.21	14.96	24	1.7%	1.26 [-6.92, 9.44]	2012
26 Mahmoud Hussein 2012	2	17	37	0	17	37	1.8%	2.00 [-5.75, 9.75]	2012
72a Milat 2015	1.75	2.04	20	-2.4	2.72	29	4.1%	4.15 [2.82, 5.48]	2015
74a Markus Lipovac 2016	5.7	20.48	156	7.9	18.4	143	2.9%	-2.20 [-6.61, 2.21]	2016
22 Abdul Razzaq 2018	23	18.73	60	0	21	60	2.0%	23.00 [15.88, 30.12]	2018
80a TinaKoldJensen 2020	46	160.66	98	42	427.12	1125	0.1%	4.00 [-36.43, 44.43]	2020
**Subtotal (95% CI)**			**825**			**1789**	**28.8%**	**5.99 [2.83, 9.15]**	
Heterogeneity: $Tau^2 = 20.00$; $Chi^2 = 135.70$, df = 12 (P < 0.00001); $I^2 = 91\%$									
Test for overall effect: Z = 3.71 (P = 0.0002)									
**Total (95% CI)**			**2132**			**5997**	**100.0%**	**4.90 [3.54, 6.26]**	
Heterogeneity: $Tau^2 = 11.31$; $Chi^2 = 320.09$, df = 41 (P < 0.00001); $I^2 = 87\%$									
Test for overall effect: Z = 7.05 (P < 0.00001)									
Test for subgroup differences: $Chi^2 = 3.73$, df = 4 (P = 0.44), $I^2 = 0\%$									

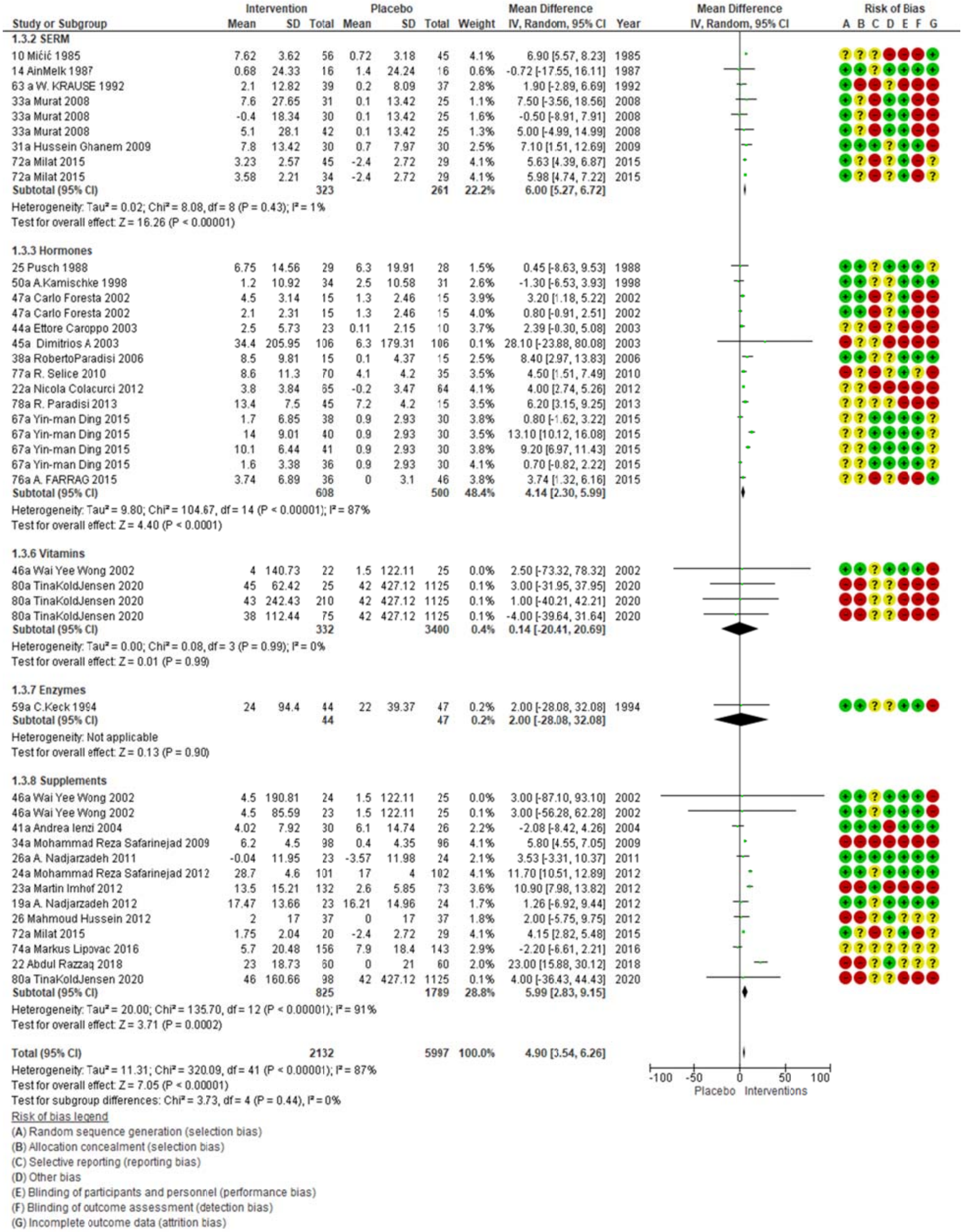

Risk of bias legend
(A) Random sequence generation (selection bias)
(B) Allocation concealment (selection bias)
(C) Selective reporting (reporting bias)
(D) Other bias
(E) Blinding of participants and personnel (performance bias)
(F) Blinding of outcome assessment (detection bias)
(G) Incomplete outcome data (attrition bias)

Supplementary Figure 5: Sub-group meta-analysis (SERM type based) for sperm concentration (n = 6).

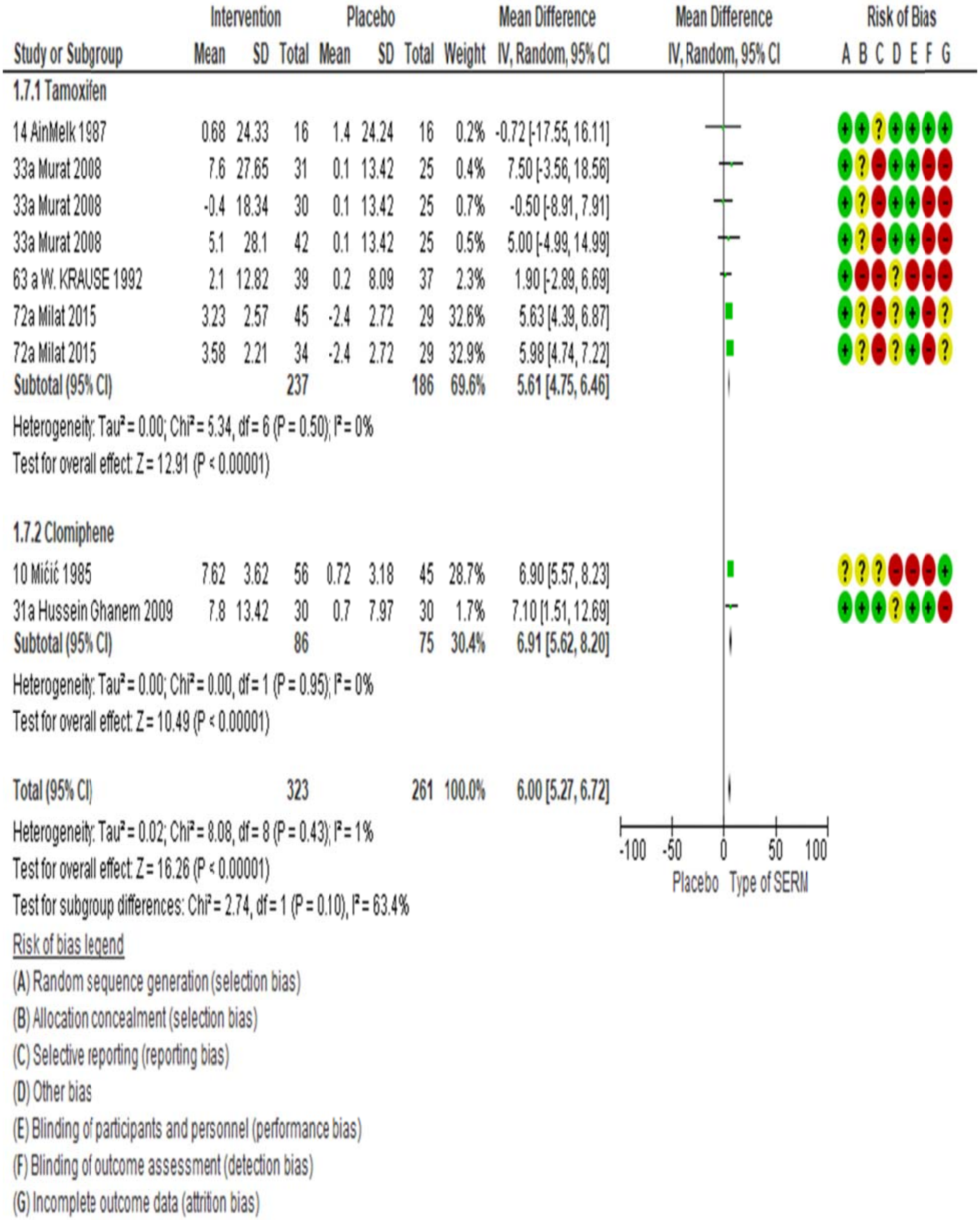

Supplementary Figure 6: Sub-group meta-analysis (Supplement type based) for sperm concentration (n = 12)

Study or Subgroup	Intervention Mean	Intervention SD	Intervention Total	Placebo Mean	Placebo SD	Placebo Total	Weight	Mean Difference IV, Random, 95% CI
**1.8.4 Zinc Sulphate**								
22 Abdul Razzaq 2018	23	18.73	60	0	21	60	7.8%	23.00 [15.88, 30.12]
26 Mahmoud Hussein 2012	2	17	37	0	17	37	7.3%	2.00 [-5.75, 9.75]
46a Wai Yee Wong 2002	4.5	85.59	23	1.5	122.11	25	0.3%	3.00 [-56.28, 62.28]
46a Wai Yee Wong 2002	4.5	190.81	24	1.5	122.11	25	0.1%	3.00 [-87.10, 93.10]
**Subtotal (95% CI)**			**144**			**147**	**15.5%**	**11.49 [-6.42, 29.39]**
Heterogeneity: Tau² = 173.83; Chi² = 15.47, df = 3 (P = 0.001); I² = 81%								
Test for overall effect: Z = 1.26 (P = 0.21)								
**1.8.5 CoQ10**								
19a A. Nadjarzadeh 2012	17.47	13.66	23	16.21	14.96	24	6.9%	1.26 [-6.92, 9.44]
24a Mohammad Reza Safarinejad 2012	28.7	4.6	101	17	4	102	12.8%	11.70 [10.51, 12.89]
26a A. Nadjarzadeh 2011	-0.04	11.95	23	-3.57	11.98	24	8.1%	3.53 [-3.31, 10.37]
34a Mohammad Reza Safarinejad 2009	6.2	4.5	98	0.4	4.35	96	12.7%	5.80 [4.55, 7.05]
**Subtotal (95% CI)**			**245**			**246**	**40.5%**	**6.53 [1.88, 11.17]**
Heterogeneity: Tau² = 17.04; Chi² = 50.76, df = 3 (P < 0.00001); I² = 94%								
Test for overall effect: Z = 2.75 (P = 0.006)								
**1.8.6 Carnitine**								
41a Andrea Ierzi 2004	4.02	7.92	30	6.1	14.74	26	8.5%	-2.08 [-8.42, 4.26]
**Subtotal (95% CI)**			**30**			**26**	**8.5%**	**-2.08 [-8.42, 4.26]**
Heterogeneity: Not applicable								
Test for overall effect: Z = 0.64 (P = 0.52)								
**1.8.7 L-Carnitine**								
72a Milat 2015	1.75	2.04	20	-2.4	2.72	29	12.7%	4.15 [2.82, 5.48]
74a Markus Lipovac 2016	5.7	20.48	156	7.9	18.4	143	10.4%	-2.20 [-6.61, 2.21]
**Subtotal (95% CI)**			**176**			**172**	**23.1%**	**1.34 [-4.85, 7.52]**
Heterogeneity: Tau² = 17.40; Chi² = 7.31, df = 1 (P = 0.007); I² = 86%								
Test for overall effect: Z = 0.42 (P = 0.67)								
**1.8.8 Fish Oil**								
80a TinaKoldJensen 2020	46	160.66	98	42	427.12	1125	0.6%	4.00 [-36.43, 44.43]
**Subtotal (95% CI)**			**98**			**1125**	**0.6%**	**4.00 [-36.43, 44.43]**
Heterogeneity: Not applicable								
Test for overall effect: Z = 0.19 (P = 0.85)								
**1.8.9 Profertil**								
23a Martin Imhof 2012	13.5	15.21	132	2.6	5.85	73	11.7%	10.90 [7.98, 13.82]
**Subtotal (95% CI)**			**132**			**73**	**11.7%**	**10.90 [7.98, 13.82]**
Heterogeneity: Not applicable								
Test for overall effect: Z = 7.31 (P < 0.00001)								
**Total (95% CI)**			**825**			**1789**	**100.0%**	**5.99 [2.83, 9.15]**

Heterogeneity: Tau² = 20.00; Chi² = 135.70, df = 12 (P < 0.00001); I² = 91%
Test for overall effect: Z = 3.71 (P = 0.0002)
Test for subgroup differences: Chi² = 18.17, df = 5 (P = 0.003), I² = 72.5%

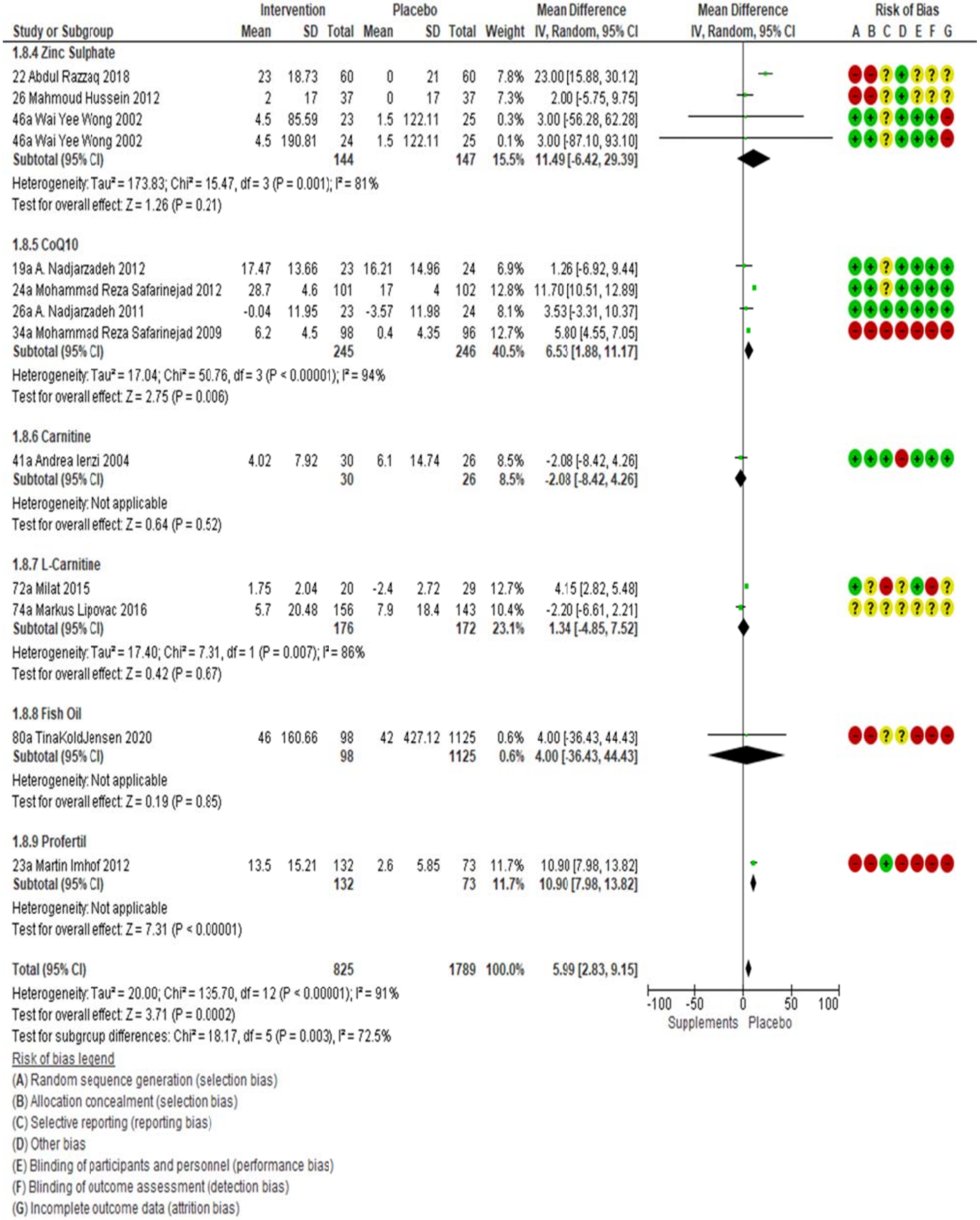

Risk of bias legend
(A) Random sequence generation (selection bias)
(B) Allocation concealment (selection bias)
(C) Selective reporting (reporting bias)
(D) Other bias
(E) Blinding of participants and personnel (performance bias)
(F) Blinding of outcome assessment (detection bias)
(G) Incomplete outcome data (attrition bias)

Supplementary Figure 7: Sub-group meta-analysis (intervention based) for sperm motility (n = 29)

Study or Subgroup	Intervention Mean	Intervention SD	Intervention Total	Placebo Mean	Placebo SD	Placebo Total	Weight	Mean Difference IV, Random, 95% CI
**1.13.2 SERM**								
10 Mićić 1985	6.24	11.85	56	-2.18	10.39	46	3.2%	8.42 [4.10, 12.74]
14 AinMelk 1987	-0.84	36.29	16	2.08	39.92	16	0.4%	-2.92 [-29.35, 23.51]
31a Hussein Ghanem 2009	1	20.07	30	-6	17.09	30	1.8%	7.00 [-2.43, 16.43]
33a Murat 2008	13.12	17.1	31	-3.02	14.4	25	2.1%	16.14 [7.89, 24.39]
33a Murat 2008	10.52	18.12	42	-3.02	14.4	25	2.2%	13.54 [5.67, 21.41]
33a Murat 2008	-5.08	13.02	30	-3.02	14.4	25	2.3%	-2.06 [-9.38, 5.26]
63 a W. KRAUSE 1992	-1.3	25.44	39	-3.1	19.25	37	1.7%	1.80 [-8.31, 11.91]
72a Milat 2015	5.76	3.4	34	-1.5	4.91	29	3.7%	7.26 [5.14, 9.38]
72a Milat 2015	2.33	3.69	45	-1.5	4.91	29	3.7%	3.83 [1.74, 5.92]
**Subtotal (95% CI)**			**323**			**262**	**21.2%**	**6.62 [3.69, 9.54]**
Heterogeneity: Tau² = 9.15; Chi² = 21.84, df = 8 (P = 0.005); I² = 63%								
Test for overall effect: Z = 4.44 (P < 0.00001)								
**1.13.3 Hormones**								
22a Nicola Colacurci 2012	2.5	5.1	65	0.6	4.91	64	3.8%	1.90 [0.17, 3.63]
25 Pusch 1988	2.8	10.04	29	1.4	12.54	28	2.7%	1.40 [-4.51, 7.31]
38a RobertoParadisi 2006	1.9	5.45	15	0.6	5.55	15	3.3%	1.30 [-2.64, 5.24]
44a Ettore Caroppo 2003	-3.82	42.7	23	-8.99	39.33	10	0.3%	5.17 [-24.81, 35.15]
45a Dimitrios A 2003	11.9	12.59	106	1.1	15.5	106	3.3%	10.80 [7.00, 14.60]
47a Carlo Foresta 2002	1.8	8.74	15	-1	7.76	15	2.7%	2.80 [-3.11, 8.71]
47a Carlo Foresta 2002	1.7	10.48	15	-1	7.76	15	2.5%	2.70 [-3.90, 9.30]
50a A.Kamischke 1998	-2.9	16.94	34	5.8	16.7	31	2.1%	-8.70 [-16.88, -0.52]
67a Yin-man Ding 2015	2.3	4.91	36	1.3	8	30	3.4%	1.00 [-2.28, 4.28]
67a Yin-man Ding 2015	6	11.14	40	1.3	8	30	3.1%	4.70 [0.22, 9.18]
67a Yin-man Ding 2015	6.2	8.98	41	1.3	8	30	3.3%	4.90 [0.93, 8.87]
67a Yin-man Ding 2015	3.9	11.25	38	1.3	8	30	3.1%	2.60 [-1.98, 7.18]
76a A. FARRAG 2015	16	12.19	36	0	7.39	46	3.1%	16.00 [11.48, 20.52]
77a R. Selice 2010	4.7	16.67	70	1.5	16.61	35	2.5%	3.20 [-3.55, 9.95]
78a R. Paradisi 2013	0.4	5.76	45	-0.1	5.31	15	3.5%	0.50 [-2.67, 3.67]
**Subtotal (95% CI)**			**608**			**500**	**42.8%**	**3.55 [1.14, 5.96]**
Heterogeneity: Tau² = 15.45; Chi² = 63.50, df = 14 (P < 0.00001); I² = 78%								
Test for overall effect: Z = 2.89 (P = 0.004)								
**1.13.4 Supplements**								
19a A. Nadjarzadeh 2012	5.78	16.88	23	0.54	18.46	24	1.7%	5.24 [-4.87, 15.35]
22 Abdul Razzaq 2018	18	12.29	60	0	9	60	3.3%	18.00 [14.15, 21.85]
23a Martin Imhof 2012	16.5	18.89	132	9.5	34.3	73	2.0%	7.00 [-1.50, 15.50]
24a Mohammad Reza Safarinejad 2012	35.8	2.7	101	25	2	102	3.9%	10.80 [10.15, 11.45]
26 Mahmoud Hussein 2012	16	7.55	37	0	7	37	3.4%	16.00 [12.68, 19.32]
26a A. Nadjarzadeh 2011	5.78	16.87	23	0.51	17.29	24	1.8%	5.27 [-4.50, 15.04]
34a Mohammad Reza Safarinejad 2009	5.4	2.31	98	0.8	2.39	96	3.9%	4.60 [3.94, 5.26]
41a Andrea Ienzi 2004	7.94	11.66	30	6.47	8.41	26	2.9%	1.47 [-3.81, 6.75]
46a Wai Yee Wong 2002	5	80.55	23	0	89.59	25	0.1%	5.00 [-43.14, 53.14]
46a Wai Yee Wong 2002	2	86.28	24	0	89.59	25	0.1%	2.00 [-47.24, 51.24]
72a Milat 2015	1.63	3.16	20	-1.5	4.91	29	3.7%	3.13 [0.87, 5.39]
74a Markus Lipovac 2016	9.2	18.2	156	15.2	16.31	143	3.3%	-6.00 [-9.91, -2.09]
80a TinaKoldJensen 2020	63.2	53.39	98	62.7	175.87	1125	1.0%	0.50 [-14.24, 15.24]
**Subtotal (95% CI)**			**825**			**1789**	**31.2%**	**6.51 [3.15, 9.86]**
Heterogeneity: Tau² = 24.12; Chi² = 291.53, df = 12 (P < 0.00001); I² = 96%								
Test for overall effect: Z = 3.80 (P = 0.0001)								
**1.13.5 Enzymes**								
59a C.Keck 1994	35.2	23.18	44	31.9	21.9	47	1.9%	3.30 [-5.98, 12.58]
**Subtotal (95% CI)**			**44**			**47**	**1.9%**	**3.30 [-5.98, 12.58]**
Heterogeneity: Not applicable								
Test for overall effect: Z = 0.70 (P = 0.49)								
**1.13.6 Vitamins**								
46a Wai Yee Wong 2002	5	69.12	22	0	89.59	25	0.1%	5.00 [-40.47, 50.47]
80a TinaKoldJensen 2020	63.6	35.58	25	62.7	175.87	1125	0.8%	0.90 [-16.42, 18.22]
80a TinaKoldJensen 2020	60.3	51.46	75	62.7	175.87	1125	1.0%	-2.40 [-17.93, 13.13]
80a TinaKoldJensen 2020	60	77.07	210	62.7	175.87	1125	1.1%	-2.70 [-17.34, 11.94]
**Subtotal (95% CI)**			**332**			**3400**	**3.0%**	**-1.36 [-10.26, 7.54]**
Heterogeneity: Tau² = 0.00; Chi² = 0.19, df = 3 (P = 0.98); I² = 0%								
Test for overall effect: Z = 0.30 (P = 0.77)								
**Total (95% CI)**			**2132**			**5998**	**100.0%**	**4.99 [3.22, 6.75]**
Heterogeneity: Tau² = 20.68; Chi² = 443.51, df = 41 (P < 0.00001); I² = 91%								
Test for overall effect: Z = 5.54 (P < 0.00001)								
Test for subgroup differences: Chi² = 5.42, df = 4 (P = 0.25), I² = 26.2%								

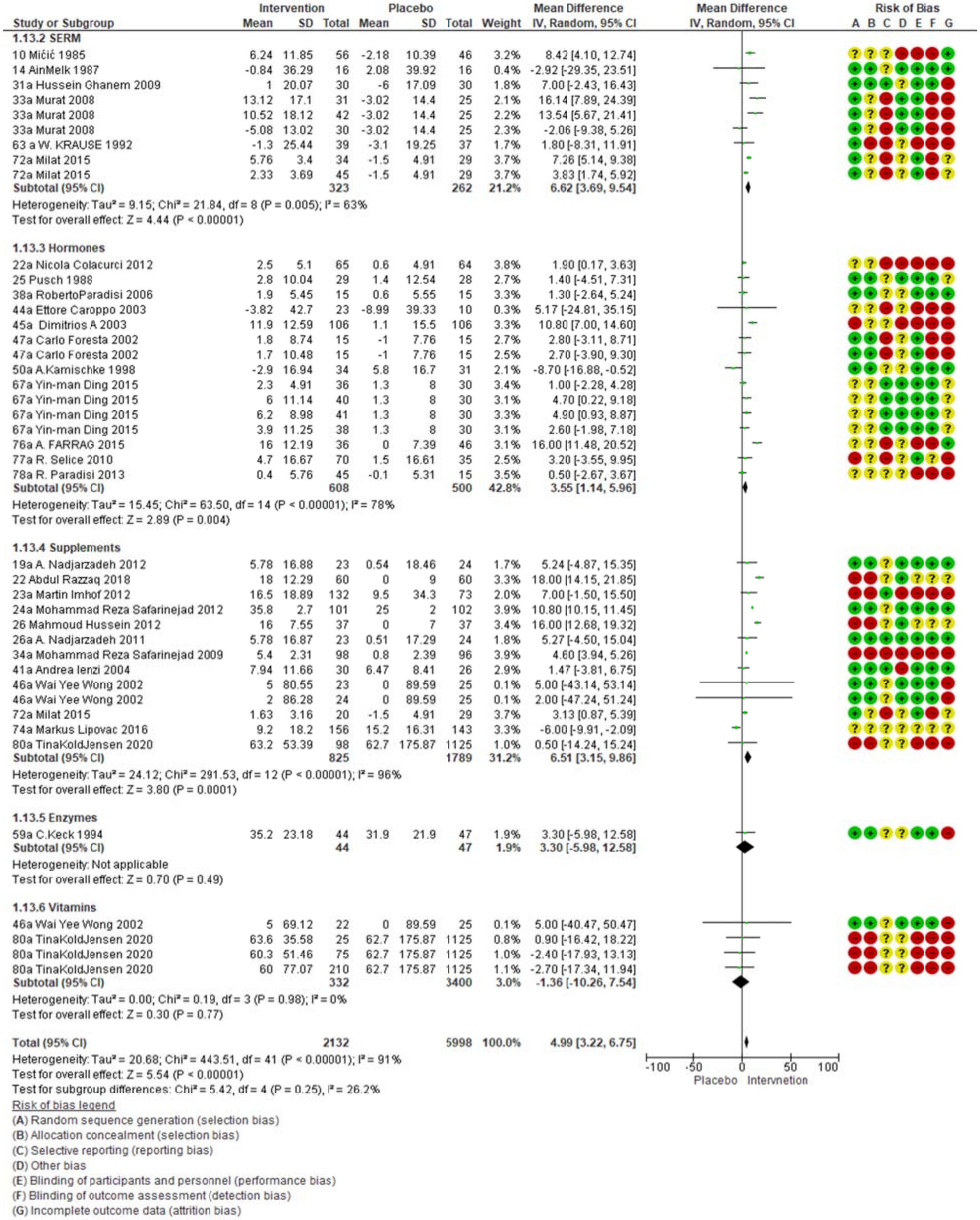

Risk of bias legend
(A) Random sequence generation (selection bias)
(B) Allocation concealment (selection bias)
(C) Selective reporting (reporting bias)
(D) Other bias
(E) Blinding of participants and personnel (performance bias)
(F) Blinding of outcome assessment (detection bias)
(G) Incomplete outcome data (attrition bias)

Supplementary Figure 8: Sub-group meta-analysis (SERM type based) for sperm motility (n = 6).

Study or Subgroup	Intervention Mean	Intervention SD	Intervention Total	Placebo Mean	Placebo SD	Placebo Total	Weight	Mean Difference IV, Random, 95% CI	Risk of Bias A B C D E F G
**1.15.1 Clomiphene**									
10 Mićić 1985	6.24	11.85	56	-2.18	10.39	46	15.9%	8.42 [4.10, 12.74]	? ? ? - - - +
31a Hussein Ghanem 2009	1	20.07	30	-6	17.09	30	6.9%	7.00 [-2.43, 16.43]	+ + + ? + + -
**Subtotal (95% CI)**			**86**			**76**	**22.8%**	**8.17 [4.25, 12.10]**	
Heterogeneity: Tau² = 0.00; Chi² = 0.07, df = 1 (P = 0.79); I² = 0%									
Test for overall effect: Z = 4.08 (P < 0.0001)									
**1.15.2 Tamoxifen**									
14 AinMelk 1987	-0.84	36.29	16	2.08	39.92	16	1.2%	-2.92 [-29.35, 23.51]	+ + ? + + + +
33a Murat 2008	13.12	17.1	31	-3.02	14.4	25	8.3%	16.14 [7.89, 24.39]	+ ? - + + - -
33a Murat 2008	10.52	18.12	42	-3.02	14.4	25	8.8%	13.54 [5.67, 21.41]	+ ? - + + - -
33a Murat 2008	-5.08	13.02	30	-3.02	14.4	25	9.6%	-2.06 [-9.38, 5.26]	+ ? - + + - -
63 a W. KRAUSE 1992	-1.3	25.44	39	-3.1	19.25	37	6.2%	1.80 [-8.31, 11.91]	+ - - ? - - -
72a Milat 2015	5.76	3.4	34	-1.5	4.91	29	21.5%	7.26 [5.14, 9.38]	+ ? - ? + - ?
72a Milat 2015	2.33	3.69	45	-1.5	4.91	29	21.6%	3.83 [1.74, 5.92]	+ ? - ? + - ?
**Subtotal (95% CI)**			**237**			**186**	**77.2%**	**6.26 [2.62, 9.90]**	
Heterogeneity: Tau² = 12.05; Chi² = 20.42, df = 6 (P = 0.002); I² = 71%									
Test for overall effect: Z = 3.37 (P = 0.0008)									
**Total (95% CI)**			**323**			**262**	**100.0%**	**6.62 [3.69, 9.54]**	
Heterogeneity: Tau² = 9.15; Chi² = 21.84, df = 8 (P = 0.005); I² = 63%									
Test for overall effect: Z = 4.44 (P < 0.00001)									
Test for subgroup differences: Chi² = 0.49, df = 1 (P = 0.48), I² = 0%									

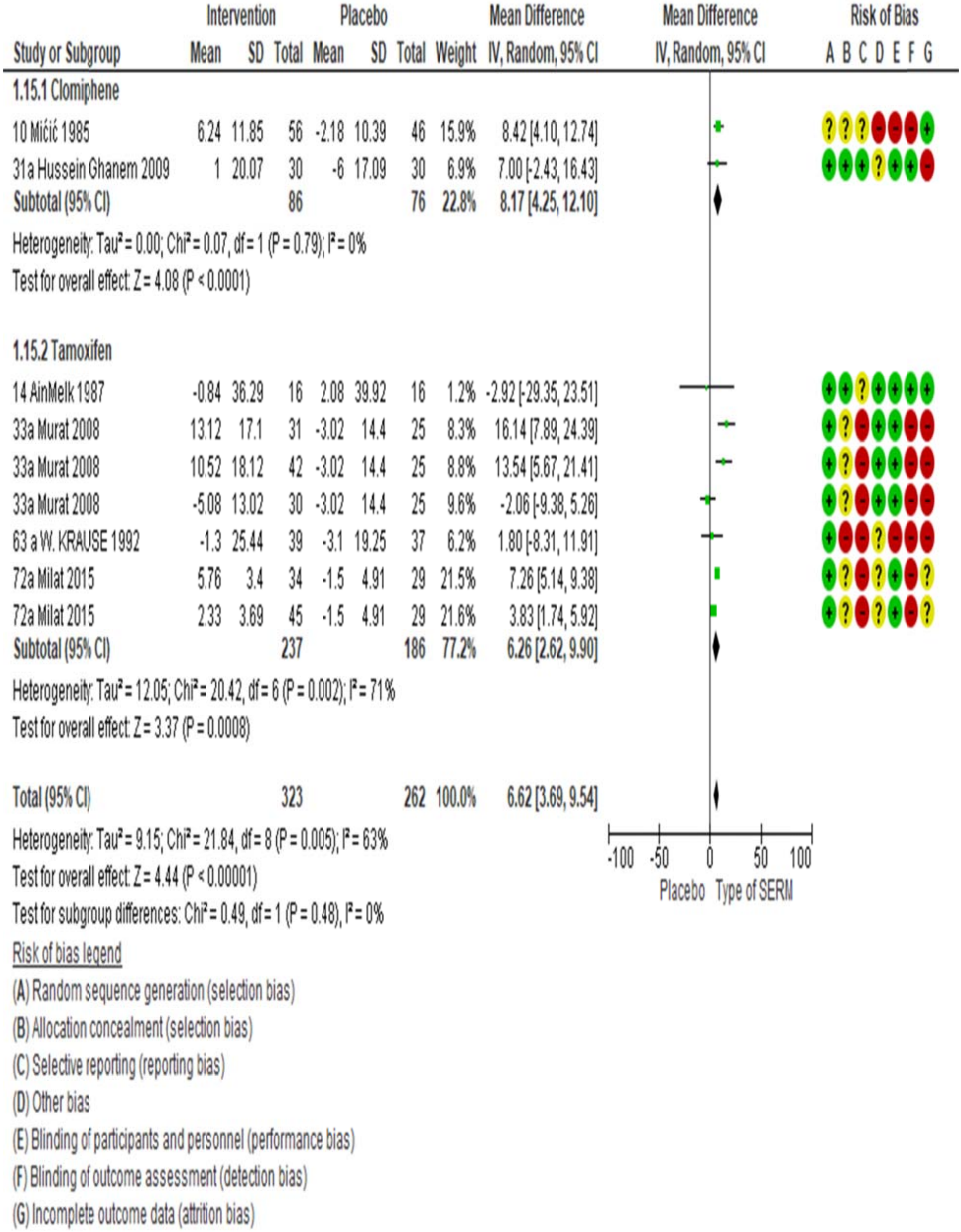

Risk of bias legend

(A) Random sequence generation (selection bias)

(B) Allocation concealment (selection bias)

(C) Selective reporting (reporting bias)

(D) Other bias

(E) Blinding of participants and personnel (performance bias)

(F) Blinding of outcome assessment (detection bias)

(G) Incomplete outcome data (attrition bias)

Supplementary Figure 9: Sub-group meta-analysis (supplement type based) for sperm motility (n = 12).

Study or Subgroup	Intervention Mean	Intervention SD	Intervention Total	Placebo Mean	Placebo SD	Placebo Total	Weight	Mean Difference IV, Random, 95% CI
**1.18.4 Zinc Sulphate**								
22 Abdul Razzaq 2018	18	12.29	60	0	9	60	10.5%	18.00 [14.15, 21.85]
26 Mahmoud Hussein 2012	16	7.55	37	0	7	37	10.9%	16.00 [12.68, 19.32]
46a Wai Yee Wong 2002	5	80.55	23	0	89.59	25	0.5%	5.00 [-43.14, 53.14]
46a Wai Yee Wong 2002	2	86.28	24	0	89.59	25	0.4%	2.00 [-47.24, 51.24]
**Subtotal (95% CI)**			**144**			**147**	**22.3%**	**16.78 [14.27, 19.29]**
Heterogeneity: Tau² = 0.00; Chi² = 1.17, df = 3 (P = 0.76); I² = 0%								
Test for overall effect: Z = 13.12 (P < 0.00001)								
**1.18.5 CoQ190**								
19a A. Nadjarzadeh 2012	5.78	16.88	23	0.54	18.46	24	5.8%	5.24 [-4.87, 15.35]
24a Mohammad Reza Safarinejad 2012	35.8	2.7	101	25	2	102	12.1%	10.80 [10.15, 11.45]
26a A. Nadjarzadeh 2011	5.78	16.87	23	0.51	17.29	24	6.0%	5.27 [-4.50, 15.04]
34a Mohammad Reza Safarinejad 2009	5.4	2.31	98	0.8	2.39	96	12.1%	4.60 [3.94, 5.26]
**Subtotal (95% CI)**			**245**			**246**	**36.0%**	**6.97 [1.94, 12.01]**
Heterogeneity: Tau² = 18.69; Chi² = 171.06, df = 3 (P < 0.00001); I² = 98%								
Test for overall effect: Z = 2.71 (P = 0.007)								
**1.18.6 Carnitine**								
41a Andrea Ienzi 2004	7.94	11.66	30	6.47	8.41	26	9.3%	1.47 [-3.81, 6.75]
**Subtotal (95% CI)**			**30**			**26**	**9.3%**	**1.47 [-3.81, 6.75]**
Heterogeneity: Not applicable								
Test for overall effect: Z = 0.55 (P = 0.59)								
**1.18.7 L-Carnitine**								
72a Milat 2015	1.63	3.16	20	-1.5	4.91	29	11.5%	3.13 [0.87, 5.39]
74a Markus Lipovac 2016	9.2	18.2	156	15.2	16.31	143	10.4%	-6.00 [-9.91, -2.09]
**Subtotal (95% CI)**			**176**			**172**	**22.0%**	**-1.29 [-10.23, 7.65]**
Heterogeneity: Tau² = 39.02; Chi² = 15.69, df = 1 (P < 0.0001); I² = 94%								
Test for overall effect: Z = 0.28 (P = 0.78)								
**1.18.8 Fish Oil**								
80a TinaKoldJensen 2020	63.2	53.39	98	62.7	175.87	1125	3.6%	0.50 [-14.24, 15.24]
**Subtotal (95% CI)**			**98**			**1125**	**3.6%**	**0.50 [-14.24, 15.24]**
Heterogeneity: Not applicable								
Test for overall effect: Z = 0.07 (P = 0.95)								
**1.18.9 Profertil**								
23a Martin Imhof 2012	16.5	18.89	132	9.5	34.3	73	6.8%	7.00 [-1.50, 15.50]
**Subtotal (95% CI)**			**132**			**73**	**6.8%**	**7.00 [-1.50, 15.50]**
Heterogeneity: Not applicable								
Test for overall effect: Z = 1.61 (P = 0.11)								
**Total (95% CI)**			**825**			**1789**	**100.0%**	**6.51 [3.15, 9.86]**

Heterogeneity: Tau² = 24.12; Chi² = 291.53, df = 12 (P < 0.00001); I² = 96%
Test for overall effect: Z = 3.80 (P = 0.0001)
Test for subgroup differences: Chi² = 45.07, df = 5 (P < 0.00001), I² = 88.9%

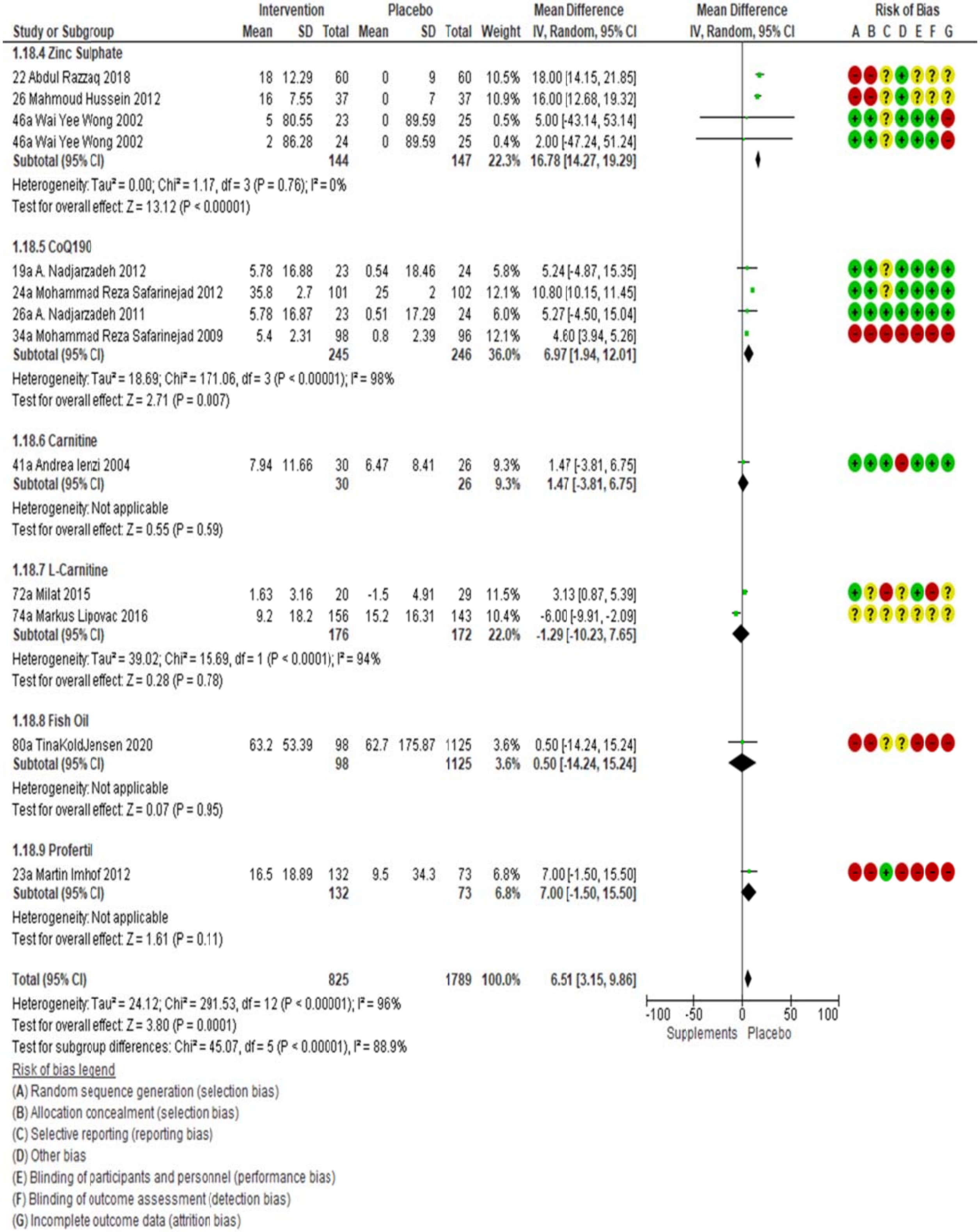

Risk of bias legend
(A) Random sequence generation (selection bias)
(B) Allocation concealment (selection bias)
(C) Selective reporting (reporting bias)
(D) Other bias
(E) Blinding of participants and personnel (performance bias)
(F) Blinding of outcome assessment (detection bias)
(G) Incomplete outcome data (attrition bias)

Supplementary Figure 10: Sub-group meta-analysis (intervention based) for sperm morphology (n=29).

Study or Subgroup	Intervention Mean	Intervention SD	Intervention Total	Placebo Mean	Placebo SD	Placebo Total	Weight	Mean Difference IV, Random, 95% CI	Year
**1.23.3 SERM**									
10 Mićić 1985	5.6	15.68	56	0.98	14.27	45	1.2%	4.62 [-1.23, 10.47]	1985
14 AinMelk 1987	-3.75	68.51	16	-2.92	70.05	16	0.0%	-0.83 [-48.84, 47.18]	1987
63 a W. KRAUSE 1992	-0.3	21.38	39	-5.2	18.03	37	0.6%	4.90 [-3.98, 13.78]	1992
33a Murat 2008	-0.08	0.6	31	0.02	0.56	25	5.5%	-0.10 [-0.40, 0.20]	2008
33a Murat 2008	-0.08	0.6	30	0.02	0.56	25	5.5%	-0.10 [-0.41, 0.21]	2008
33a Murat 2008	-0.08	0.6	42	0.02	0.56	25	5.5%	-0.10 [-0.38, 0.18]	2008
31a Hussein Ghanem 2009	3	17.09	30	-10	14.53	30	0.7%	13.00 [4.97, 21.03]	2009
72a Milat 2015	0.56	0.71	45	-1	1.2	29	5.4%	1.56 [1.08, 2.04]	2015
72a Milat 2015	1.12	0.62	34	-1	1.2	29	5.4%	2.12 [1.64, 2.60]	2015
**Subtotal (95% CI)**			**323**			**261**	**29.6%**	**0.88 [0.07, 1.69]**	
Heterogeneity: $Tau^2 = 0.85$; $Chi^2 = 113.89$, df = 8 ($P < 0.00001$); $I^2 = 93\%$									
Test for overall effect: $Z = 2.13$ ($P = 0.03$)									
**1.23.4 Hormones**									
25 Pusch 1988	6.86	15.49	29	1	16.61	28	0.7%	5.86 [-2.48, 14.20]	1988
50a A.Kamischke 1998	0.2	7.06	34	-0.2	6.98	31	2.5%	0.40 [-3.02, 3.82]	1998
47a Carlo Foresta 2002	-0.7	9.72	15	1.2	9.64	15	0.9%	-1.90 [-8.83, 5.03]	2002
47a Carlo Foresta 2002	5.4	8.58	15	1.2	9.64	15	1.0%	4.20 [-2.33, 10.73]	2002
44a Ettore Caroppo 2003	5.2	8.83	23	-2.7	12.24	10	0.6%	7.90 [-0.50, 16.30]	2003
45a Dimitrios A 2003	15.4	12.93	106	-2	15.89	106	2.1%	17.40 [13.50, 21.30]	2003
38a RobertoParadisi 2006	0.9	8.7	15	-1.7	8.78	15	1.1%	2.60 [-3.66, 8.86]	2006
77a R. Selice 2010	2.2	7.76	70	-1.4	6.21	35	3.1%	3.60 [0.85, 6.35]	2010
22a Nicola Colacurci 2012	1.3	7.11	65	-0.1	7.52	64	3.3%	1.40 [-1.13, 3.93]	2012
78a R. Paradisi 2013	0.2	7.45	45	-2.6	8.78	15	1.5%	2.80 [-2.15, 7.75]	2013
67a Yin-man Ding 2015	6.1	11.43	41	2.2	9.85	30	1.5%	3.90 [-1.07, 8.87]	2015
76a A. FARRAG 2015	6.36	8.34	36	0	7	46	2.5%	6.36 [2.97, 9.75]	2015
67a Yin-man Ding 2015	-2.5	3.48	36	2.2	9.85	30	2.2%	-4.70 [-8.40, -1.00]	2015
67a Yin-man Ding 2015	6.4	8.51	38	2.2	9.85	30	1.8%	4.20 [-0.24, 8.64]	2015
67a Yin-man Ding 2015	4.4	11.43	40	2.2	9.85	30	1.5%	2.20 [-2.80, 7.20]	2015
**Subtotal (95% CI)**			**608**			**500**	**26.2%**	**3.68 [0.97, 6.39]**	
Heterogeneity: $Tau^2 = 22.07$; $Chi^2 = 80.44$, df = 14 ($P < 0.00001$); $I^2 = 83\%$									
Test for overall effect: $Z = 2.66$ ($P = 0.008$)									
**1.23.5 Supplements**									
46a Wai Yee Wong 2002	-1	7.28	23	0	9.42	25	1.6%	-1.00 [-5.74, 3.74]	2002
46a Wai Yee Wong 2002	0	14.1	24	0	9.42	25	0.9%	0.00 [-6.74, 6.74]	2002
41a Andrea Ienzi 2004	1.28	2.41	30	4.3	3.2	26	4.4%	-3.02 [-4.52, -1.52]	2004
34a Mohammad Reza Safarinejad 2009	2.4	2.51	98	0.5	2.46	96	5.2%	1.90 [1.20, 2.60]	2009
26a A. Nadjarzadeh 2011	-0.91	5.1	23	0.21	5.08	24	2.9%	-1.12 [-4.03, 1.79]	2011
26 Mahmoud Hussein 2012	5	10.58	37	0	12	37	1.4%	5.00 [-0.15, 10.15]	2012
23a Martin Imhof 2012	11	12.16	132	-3.5	30.02	73	0.8%	14.50 [7.31, 21.69]	2012
24a Mohammad Reza Safarinejad 2012	17.6	4.4	101	14.8	4.1	102	4.8%	2.80 [1.63, 3.97]	2012
19a A. Nadjarzadeh 2012	-0.91	4.81	23	0.21	4.92	24	3.0%	-1.12 [-3.90, 1.66]	2012
72a Milat 2015	2.01	0.23	20	-1	1.2	29	5.4%	3.01 [2.56, 3.46]	2015
74a Markus Lipovac 2016	9.6	15.16	156	9.7	15.86	143	2.4%	-0.10 [-3.62, 3.42]	2016
22 Abdul Razzaq 2018	12	9.64	60	0	11	60	2.2%	12.00 [8.30, 15.70]	2018
80a TinaKoldJensen 2020	7.5	13.34	98	6.5	50.24	1125	2.1%	1.00 [-2.95, 4.95]	2020
**Subtotal (95% CI)**			**825**			**1789**	**37.4%**	**1.93 [0.43, 3.43]**	
Heterogeneity: $Tau^2 = 4.91$; $Chi^2 = 113.80$, df = 12 ($P < 0.00001$); $I^2 = 89\%$									
Test for overall effect: $Z = 2.52$ ($P = 0.01$)									
**1.23.6 Enzymes**									
59a C.Keck 1994	16	24.84	44	13	32.52	47	0.3%	3.00 [-8.85, 14.85]	1994
**Subtotal (95% CI)**			**44**			**47**	**0.3%**	**3.00 [-8.85, 14.85]**	
Heterogeneity: Not applicable									
Test for overall effect: $Z = 0.50$ ($P = 0.62$)									
**1.23.7 Vitamins**									
46a Wai Yee Wong 2002	0	11.29	22	0	9.42	25	1.1%	0.00 [-5.99, 5.99]	2002
80a TinaKoldJensen 2020	6.5	10.36	25	6.5	50.24	1125	1.5%	0.00 [-5.01, 5.01]	2020
80a TinaKoldJensen 2020	6	11.02	75	6.5	50.24	1125	2.1%	-0.50 [-4.35, 3.35]	2020
80a TinaKoldJensen 2020	6.8	27.13	210	6.5	50.24	1125	1.6%	0.30 [-4.40, 5.00]	2020
**Subtotal (95% CI)**			**332**			**3400**	**6.4%**	**-0.11 [-2.47, 2.24]**	
Heterogeneity: $Tau^2 = 0.00$; $Chi^2 = 0.07$, df = 3 ($P = 0.99$); $I^2 = 0\%$									
Test for overall effect: $Z = 0.09$ ($P = 0.93$)									
**Total (95% CI)**			**2132**			**5997**	**100.0%**	**1.96 [1.24, 2.68]**	
Heterogeneity: $Tau^2 = 2.45$; $Chi^2 = 451.40$, df = 41 ($P < 0.00001$); $I^2 = 91\%$									
Test for overall effect: $Z = 5.33$ ($P < 0.00001$)									
Test for subgroup differences: $Chi^2 = 6.00$, df = 4 ($P = 0.20$), $I^2 = 33.3\%$									

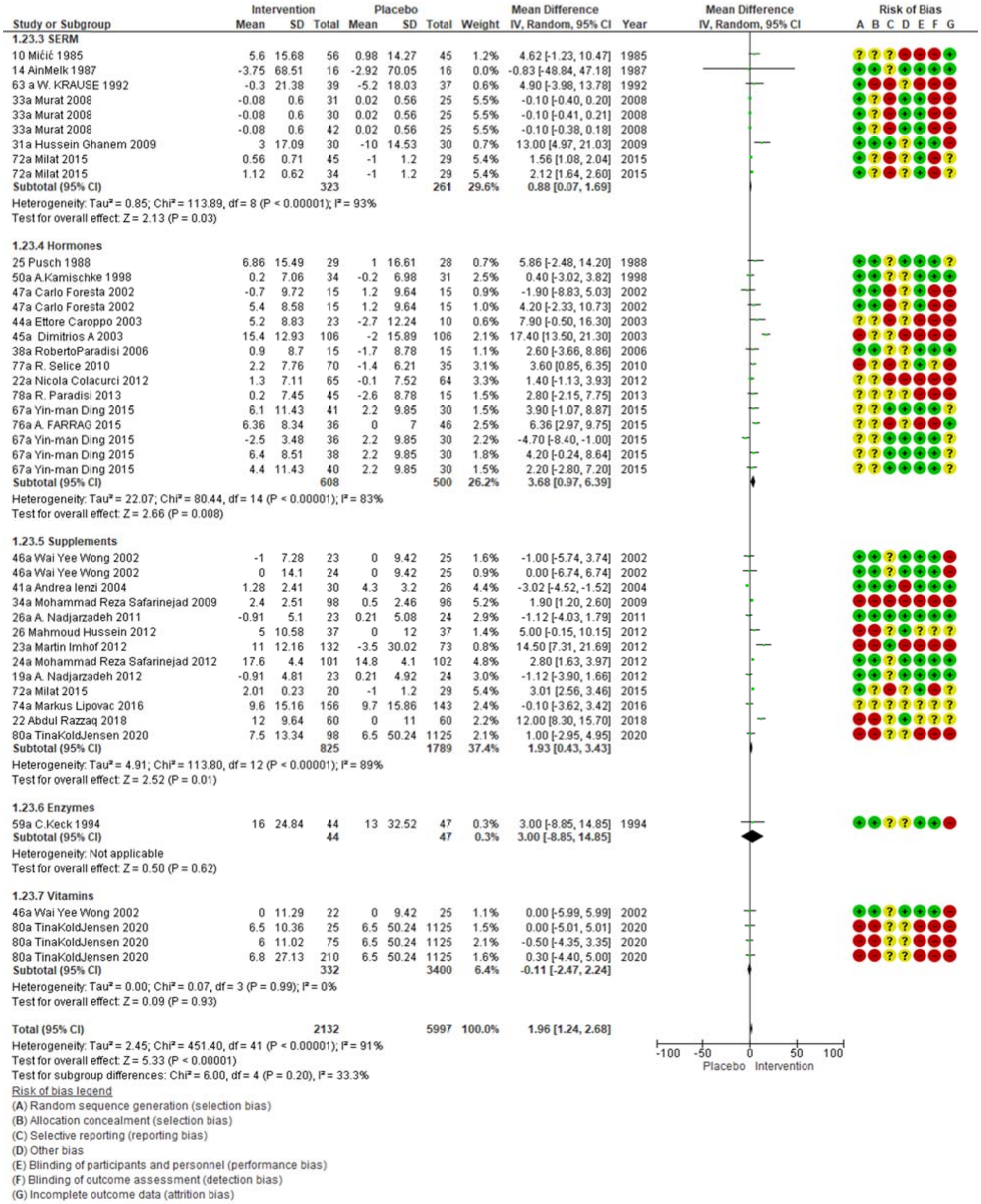

Risk of bias legend
(A) Random sequence generation (selection bias)
(B) Allocation concealment (selection bias)
(C) Selective reporting (reporting bias)
(D) Other bias
(E) Blinding of participants and personnel (performance bias)
(F) Blinding of outcome assessment (detection bias)
(G) Incomplete outcome data (attrition bias)

Supplementary Figure 11: Sub-group meta-analysis (hormone type based) for sperm morphology (n = 12)

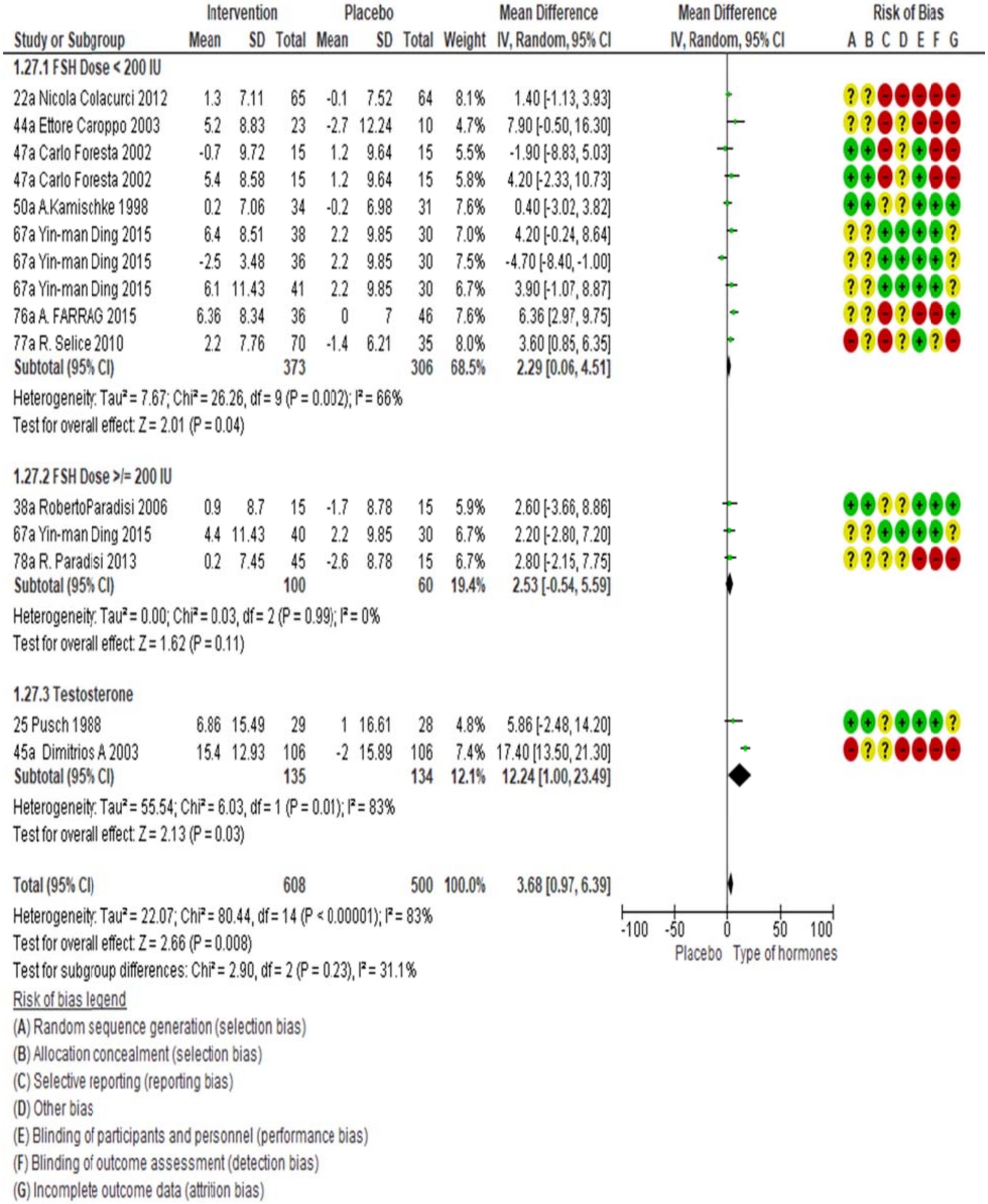

Supplementary Figure 12: Sub-group meta-analysis (supplement type based) for sperm morphology (n=12)

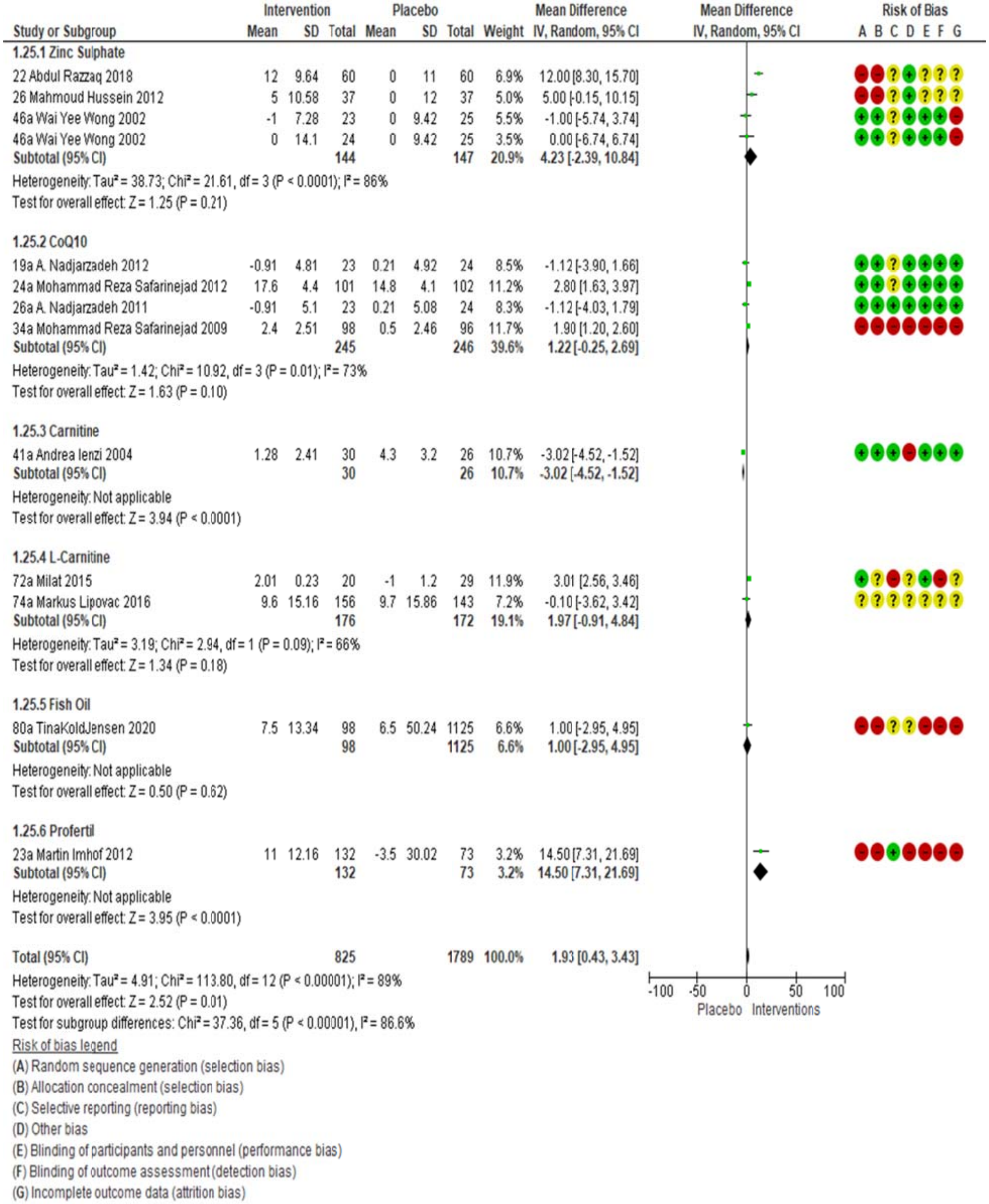

Supplementary Figure 13: Overall meta-analysis for total serum testosterone of included RCTs (n=17).

Study or Subgroup	Intervention Mean	Intervention SD	Intervention Total	Placebo Mean	Placebo SD	Placebo Total	Weight	Mean Difference IV, Random, 95% CI
14 AinMelk 1987	3.42	1.32	16	0	1.05	16	7.9%	3.42 [2.59, 4.25]
24a Mohammad Reza Safarinejad 2012	19.6	4.6	101	16.7	4.4	102	4.5%	2.90 [1.66, 4.14]
25 Pusch 1988	0.45	5.37	29	-2.08	3.99	28	1.4%	2.53 [0.08, 4.98]
33a Murat 2008	1.12	0.24	42	-0.03	0.2	25	19.3%	1.15 [1.04, 1.26]
33a Murat 2008	1.07	0.22	30	-0.03	0.2	25	19.3%	1.10 [0.99, 1.21]
33a Murat 2008	1.4	0.24	31	-0.03	0.2	25	19.3%	1.43 [1.31, 1.55]
34a Mohammad Reza Safarinejad 2009	2.8	5.5	98	0.2	4.7	96	3.6%	2.60 [1.16, 4.04]
38a RobertoParadisi 2006	0.1	1.75	15	0.2	1.61	15	4.7%	-0.10 [-1.30, 1.10]
5 Helo 2015	1.6	1.78	13	3.18	1.61	13	4.2%	-1.58 [-2.88, -0.28]
50a A.Kamischke 1998	1.2	5.18	34	0.5	3.63	31	1.8%	0.70 [-1.46, 2.86]
63 a W. KRAUSE 1992	3	3.12	39	0.3	2	37	4.9%	2.70 [1.53, 3.87]
77a R. Selice 2010	0.3	5.61	70	0.3	5.85	35	1.5%	0.00 [-2.34, 2.34]
78a R. Paradisi 2013	-0.1	1.47	45	-0.3	1.51	15	7.3%	0.20 [-0.68, 1.08]
80a TinaKoldJensen 2020	20.6	17.79	98	21.1	164.14	1125	0.1%	-0.50 [-10.72, 9.72]
80a TinaKoldJensen 2020	20.6	28.58	210	21.1	164.14	1125	0.1%	-0.50 [-10.84, 9.84]
80a TinaKoldJensen 2020	20.6	16.86	75	21.1	164.14	1125	0.1%	-0.50 [-10.82, 9.82]
80a TinaKoldJensen 2020	20	9.48	25	21.1	164.14	1125	0.1%	-1.10 [-11.39, 9.19]
**Total (95% CI)**			**971**			**4963**	**100.0%**	**1.33 [1.03, 1.62]**

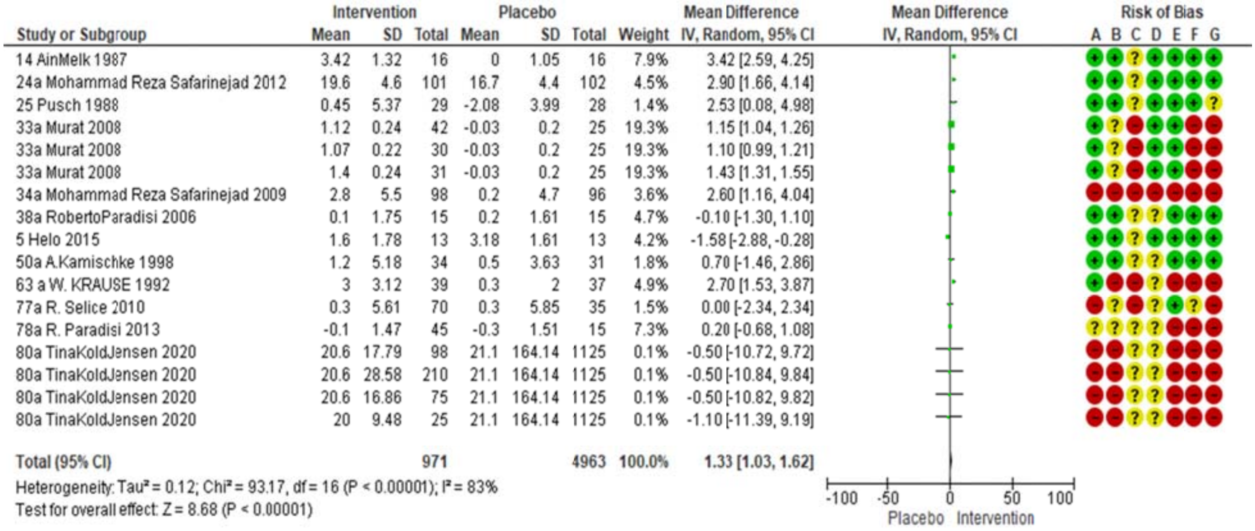

Heterogeneity: Tau² = 0.12; Chi² = 93.17, df = 16 (P < 0.00001); I² = 83%
Test for overall effect: Z = 8.68 (P < 0.00001)

Risk of bias legend
(A) Random sequence generation (selection bias)
(B) Allocation concealment (selection bias)
(C) Selective reporting (reporting bias)
(D) Other bias
(E) Blinding of participants and personnel (performance bias)
(F) Blinding of outcome assessment (detection bias)
(G) Incomplete outcome data (attrition bias)

Supplementary Figure 14: Overall meta-analysis for total serum FSH of included RCTs (n=17).

Study or Subgroup	Intervention Mean	Intervention SD	Intervention Total	Placebo Mean	Placebo SD	Placebo Total	Weight	Mean Difference IV, Random, 95% CI
14 AinMelk 1987	3.54	2.8	16	0	2.28	16	6.1%	3.54 [1.77, 5.31]
24a Mohammad Reza Safarinejad 2012	9.6	3.6	101	16.4	4.2	102	6.3%	-6.80 [-7.88, -5.72]
25 Pusch 1988	-0.68	1.59	29	0.43	2.15	28	6.3%	-1.11 [-2.09, -0.13]
33a Murat 2008	7.1	4.56	30	0.3	2.67	25	6.0%	6.80 [4.86, 8.74]
33a Murat 2008	1.2	2.33	31	0.3	2.67	25	6.2%	0.90 [-0.43, 2.23]
33a Murat 2008	6.3	4.1	42	0.3	2.67	25	6.1%	6.00 [4.38, 7.62]
34a Mohammad Reza Safarinejad 2009	-6	4.1	98	0.2	4.15	96	6.3%	-6.20 [-7.36, -5.04]
38a RobertoParadisi 2006	1.7	1.97	15	-0.2	1.61	15	6.3%	1.90 [0.61, 3.19]
5 Helo 2015	4.1	8.92	13	3.5	42.34	13	0.5%	0.60 [-22.92, 24.12]
50a A.Kamischke 1998	3.7	2.67	34	-0.5	3.1	31	6.2%	4.20 [2.79, 5.61]
63 a W. KRAUSE 1992	0.5	4.49	39	-0.7	3.72	37	6.0%	1.20 [-0.65, 3.05]
77a R. Selice 2010	1.6	1.91	70	0.2	2.17	35	6.4%	1.40 [0.55, 2.25]
78a R. Paradisi 2013	-0.1	1.28	45	-0.2	1.67	15	6.4%	0.10 [-0.82, 1.02]
80a TinaKoldJensen 2020	2.8	1.99	25	2.7	16.74	1125	6.3%	0.10 [-1.15, 1.35]
80a TinaKoldJensen 2020	2.4	3.95	98	2.7	16.74	1125	6.3%	-0.30 [-1.55, 0.95]
80a TinaKoldJensen 2020	2.7	7.96	210	2.7	16.74	1125	6.2%	0.00 [-1.45, 1.45]
80a TinaKoldJensen 2020	2.7	4.32	75	2.7	16.74	1125	6.2%	0.00 [-1.38, 1.38]
**Total (95% CI)**			**971**			**4963**	**100.0%**	**0.69 [-1.00, 2.38]**

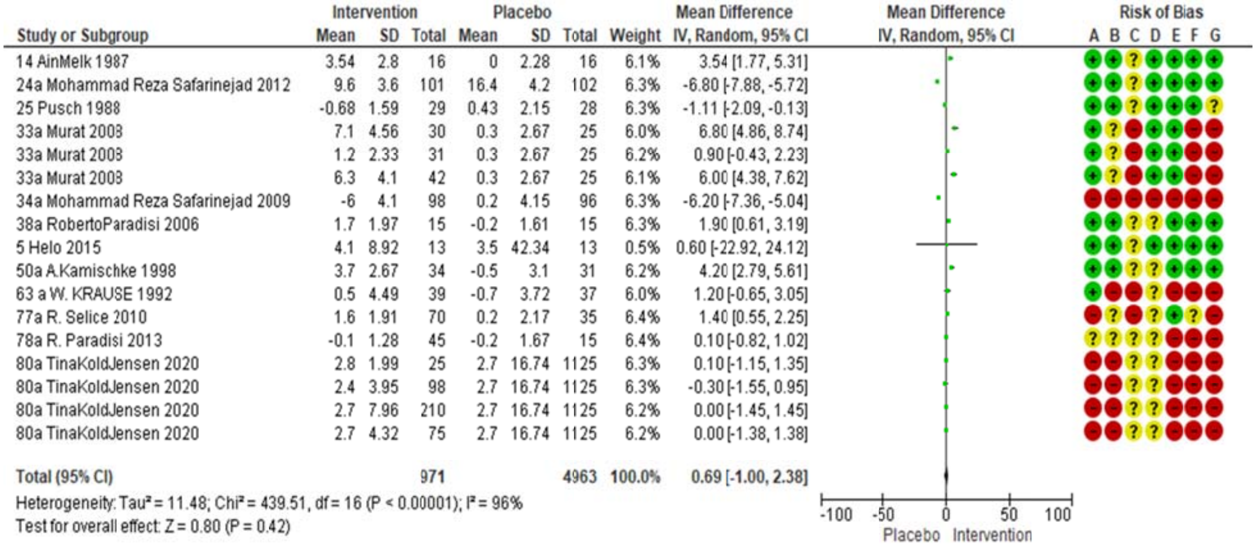

Heterogeneity: Tau² = 11.48; Chi² = 439.51, df = 16 (P < 0.00001); I² = 96%
Test for overall effect: Z = 0.80 (P = 0.42)

Risk of bias legend
(A) Random sequence generation (selection bias)
(B) Allocation concealment (selection bias)
(C) Selective reporting (reporting bias)
(D) Other bias
(E) Blinding of participants and personnel (performance bias)
(F) Blinding of outcome assessment (detection bias)
(G) Incomplete outcome data (attrition bias)

Supplementary Figure 15: Sub-group meta-analysis (intervention based) for total serum testosterone (n = 12).

Study or Subgroup	Intervention Mean	Intervention SD	Intervention Total	Placebo Mean	Placebo SD	Placebo Total	Weight	Mean Difference IV, Random, 95% CI	Risk of Bias A B C D E F G
**2.2.2 Hormones**									
25 Pusch 1988	0.45	5.37	29	-2.08	3.99	28	1.4%	2.53 [0.08, 4.98]	+ + ? + + + ?
38a RobertoParadisi 2006	0.1	1.75	15	0.2	1.61	15	4.7%	-0.10 [-1.30, 1.10]	+ + ? ? + + +
5 Helo 2015	1.6	1.78	13	3.18	1.62	13	4.1%	-1.58 [-2.89, -0.27]	+ + ? + + + +
50a A.Kamischke 1998	1.2	5.18	34	0.5	3.63	31	1.8%	0.70 [-1.46, 2.86]	+ + ? ? + + +
77a R. Selice 2010	0.3	5.61	70	0.3	5.85	35	1.5%	0.00 [-2.34, 2.34]	- ? - ? + ? -
78a R. Paradisi 2013	-0.1	1.47	45	-0.3	1.51	15	7.3%	0.20 [-0.68, 1.08]	? ? ? ? - - -
**Subtotal (95% CI)**			**206**			**137**	**20.8%**	**0.04 [-0.86, 0.94]**	
Heterogeneity: Tau² = 0.60; Chi² = 10.30, df = 5 (P = 0.07); I² = 51%									
Test for overall effect: Z = 0.09 (P = 0.93)									
**2.2.3 Vitamins**									
80a TinaKoldJensen 2020	20	9.48	25	21.1	164.14	1125	0.1%	-1.10 [-11.39, 9.19]	- - ? ? - - -
80a TinaKoldJensen 2020	20.6	16.86	75	21.1	164.14	1125	0.1%	-0.50 [-10.82, 9.82]	- - ? ? - - -
80a TinaKoldJensen 2020	20.6	28.58	210	21.1	164.14	1125	0.1%	-0.50 [-10.84, 9.84]	- - ? ? - - -
**Subtotal (95% CI)**			**310**			**3375**	**0.3%**	**-0.70 [-6.66, 5.26]**	
Heterogeneity: Tau² = 0.00; Chi² = 0.01, df = 2 (P = 1.00); I² = 0%									
Test for overall effect: Z = 0.23 (P = 0.82)									
**2.2.4 Supplements**									
24a Mohammad Reza Safarinejad 2012	19.6	4.6	101	16.7	4.4	102	4.5%	2.90 [1.66, 4.14]	+ + ? + + + +
34a Mohammad Reza Safarinejad 2009	2.8	5.5	98	0.2	4.7	96	3.6%	2.60 [1.16, 4.04]	- - - - - - -
80a TinaKoldJensen 2020	20.6	17.79	98	21.1	164.14	1125	0.1%	-0.50 [-10.72, 9.72]	- - ? ? - - -
**Subtotal (95% CI)**			**297**			**1323**	**8.1%**	**2.74 [1.81, 3.68]**	
Heterogeneity: Tau² = 0.00; Chi² = 0.49, df = 2 (P = 0.78); I² = 0%									
Test for overall effect: Z = 5.76 (P < 0.00001)									
**2.2.5 SERM**									
14 AinMelk 1987	3.42	1.32	16	0	1.05	16	7.9%	3.42 [2.59, 4.25]	+ + ? + + + +
33a Murat 2008	1.12	0.24	42	-0.03	0.2	25	19.4%	1.15 [1.04, 1.26]	+ ? - + + - -
33a Murat 2008	1.07	0.22	30	-0.03	0.2	25	19.3%	1.10 [0.99, 1.21]	+ ? - + + - -
33a Murat 2008	1.4	0.24	31	-0.03	0.2	25	19.3%	1.43 [1.31, 1.55]	+ ? - + + - -
63 a W. KRAUSE 1992	3	3.12	39	0.3	2	37	4.9%	2.70 [1.53, 3.87]	+ - - ? - - -
**Subtotal (95% CI)**			**158**			**128**	**70.8%**	**1.50 [1.20, 1.79]**	
Heterogeneity: Tau² = 0.08; Chi² = 51.94, df = 4 (P < 0.00001); I² = 92%									
Test for overall effect: Z = 9.98 (P < 0.00001)									
**Total (95% CI)**			**971**			**4963**	**100.0%**	**1.33 [1.03, 1.63]**	
Heterogeneity: Tau² = 0.12; Chi² = 93.07, df = 16 (P < 0.00001); I² = 83%									
Test for overall effect: Z = 8.69 (P < 0.00001)									
Test for subgroup differences: Chi² = 17.39, df = 3 (P = 0.0006), I² = 82.8%									

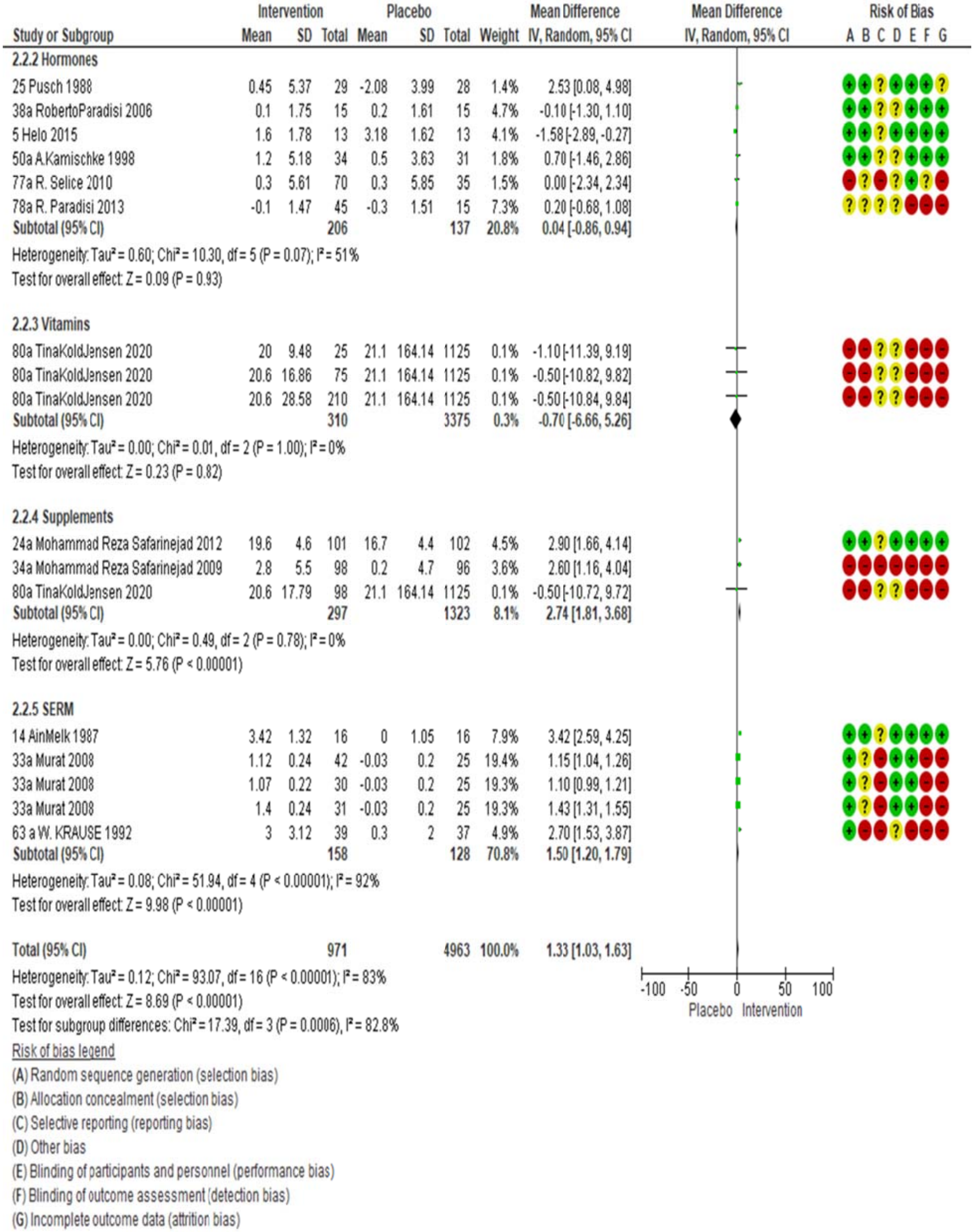

Risk of bias legend
(A) Random sequence generation (selection bias)
(B) Allocation concealment (selection bias)
(C) Selective reporting (reporting bias)
(D) Other bias
(E) Blinding of participants and personnel (performance bias)
(F) Blinding of outcome assessment (detection bias)
(G) Incomplete outcome data (attrition bias)

Supplementary Figure 16: Sub-group meta-analysis (supplement type based) for total serum testosterone (n = 3).

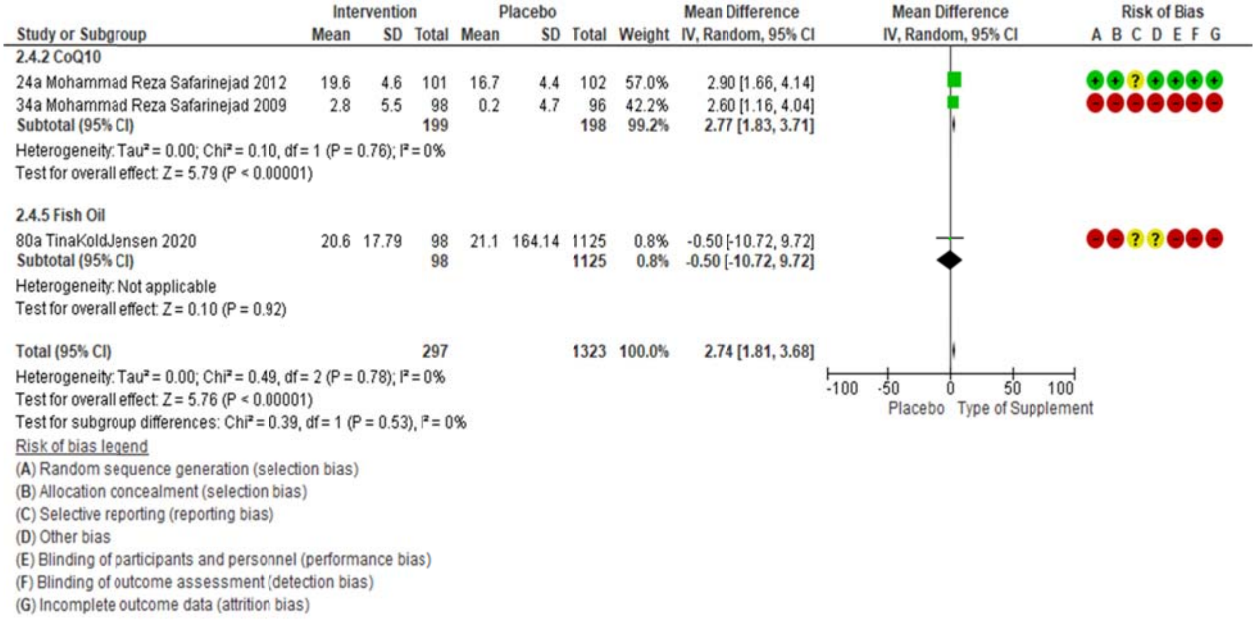

Supplementary Figure 17: Sub-group meta-analysis (SERM type based) for total serum testosterone (n = 3).

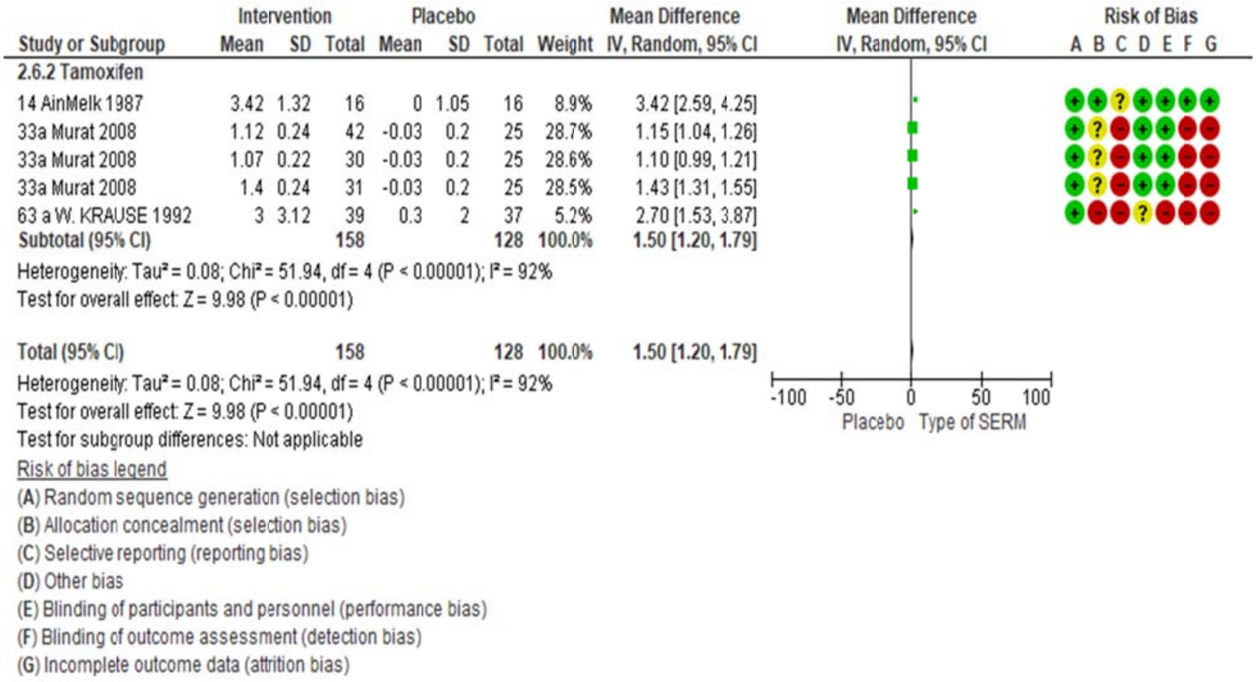

Supplementary Figure 18: Sub-group meta-analysis (intervention based) for total serum FSH (n = 12).

Study or Subgroup	Intervention Mean	Intervention SD	Intervention Total	Placebo Mean	Placebo SD	Placebo Total	Weight	Mean Difference IV, Random, 95% CI	Year	A	B	C	D	E	F	G
**2.10.2 Hormones**																
25 Pusch 1988	-0.68	1.59	29	0.43	2.15	28	6.3%	-1.11 [-2.09, -0.13]	1988	+	+	?	+	+	+	?
50a A.Kamischke 1998	3.7	2.67	34	-0.5	3.1	31	6.2%	4.20 [2.79, 5.61]	1998	+	+	?	?	+	+	+
38a RobertoParadisi 2006	1.7	1.97	15	-0.2	1.61	15	6.3%	1.90 [0.61, 3.19]	2006	+	+	?	?	+	+	+
77a R. Selice 2010	1.6	1.91	70	0.2	2.17	35	6.4%	1.40 [0.55, 2.25]	2010	-	?	-	?	+	?	-
78a R. Paradisi 2013	-0.1	1.28	45	-0.2	1.67	15	6.4%	0.10 [-0.82, 1.02]	2013	?	?	?	?	-	-	-
5 Helo 2015	4.1	8.92	13	3.5	42.34	13	0.5%	0.60 [-22.92, 24.12]	2015	+	+	?	+	+	+	+
**Subtotal (95% CI)**			**206**			**137**	**32.0%**	**1.24 [-0.29, 2.77]**								
Heterogeneity: $Tau^2 = 2.75$; $Chi^2 = 43.50$, df = 5 (P < 0.00001); $I^2 = 89\%$																
Test for overall effect: Z = 1.58 (P = 0.11)																
**2.10.3 Vitamins**																
80a TinaKoldJensen 2020	2.8	1.99	25	2.7	16.74	1125	6.3%	0.10 [-1.15, 1.35]	2020	-	-	?	?	-	-	-
80a TinaKoldJensen 2020	2.7	7.96	210	2.7	16.74	1125	6.2%	0.00 [-1.45, 1.45]	2020	-	-	?	?	-	-	-
80a TinaKoldJensen 2020	2.7	4.32	75	2.7	16.74	1125	6.2%	0.00 [-1.38, 1.38]	2020	-	-	?	?	-	-	-
**Subtotal (95% CI)**			**310**			**3375**	**18.7%**	**0.04 [-0.74, 0.82]**								
Heterogeneity: $Tau^2 = 0.00$; $Chi^2 = 0.01$, df = 2 (P = 0.99); $I^2 = 0\%$																
Test for overall effect: Z = 0.10 (P = 0.92)																
**2.10.4 SERM**																
14 AinMelk 1987	3.54	2.8	16	0	2.28	16	6.1%	3.54 [1.77, 5.31]	1987	+	+	?	+	+	+	+
63 a W. KRAUSE 1992	0.5	4.49	39	-0.7	3.72	37	6.0%	1.20 [-0.65, 3.05]	1992	+	-	-	?	-	-	-
33a Murat 2008	6.3	4.1	42	0.3	2.67	25	6.1%	6.00 [4.38, 7.62]	2008	+	?	-	+	+	-	-
33a Murat 2008	1.2	2.33	31	0.3	2.67	25	6.2%	0.90 [-0.43, 2.23]	2008	+	?	-	+	+	-	-
33a Murat 2008	7.1	4.56	30	0.3	2.67	25	6.0%	6.80 [4.86, 8.74]	2008	+	?	-	+	+	-	-
**Subtotal (95% CI)**			**158**			**128**	**30.4%**	**3.66 [1.27, 6.05]**								
Heterogeneity: $Tau^2 = 6.69$; $Chi^2 = 40.68$, df = 4 (P < 0.00001); $I^2 = 90\%$																
Test for overall effect: Z = 3.00 (P = 0.003)																
**2.10.5 Supplements**																
34a Mohammad Reza Safarinejad 2009	-6	4.1	98	0.2	4.15	96	6.3%	-6.20 [-7.36, -5.04]	2009	-	-	-	-	-	-	-
24a Mohammad Reza Safarinejad 2012	9.6	3.6	101	16.4	4.2	102	6.3%	-6.80 [-7.88, -5.72]	2012	+	+	?	+	+	+	+
80a TinaKoldJensen 2020	2.4	3.95	98	2.7	16.74	1125	6.3%	-0.30 [-1.55, 0.95]	2020	-	-	?	?	-	-	-
**Subtotal (95% CI)**			**297**			**1323**	**18.9%**	**-4.44 [-8.36, -0.52]**								
Heterogeneity: $Tau^2 = 11.65$; $Chi^2 = 68.44$, df = 2 (P < 0.00001); $I^2 = 97\%$																
Test for overall effect: Z = 2.22 (P = 0.03)																
**Total (95% CI)**			**971**			**4963**	**100.0%**	**0.69 [-1.00, 2.38]**								
Heterogeneity: $Tau^2 = 11.48$; $Chi^2 = 439.51$, df = 16 (P < 0.00001); $I^2 = 96\%$																
Test for overall effect: Z = 0.80 (P = 0.42)																
Test for subgroup differences: $Chi^2 = 14.96$, df = 3 (P = 0.002), $I^2 = 79.9\%$																

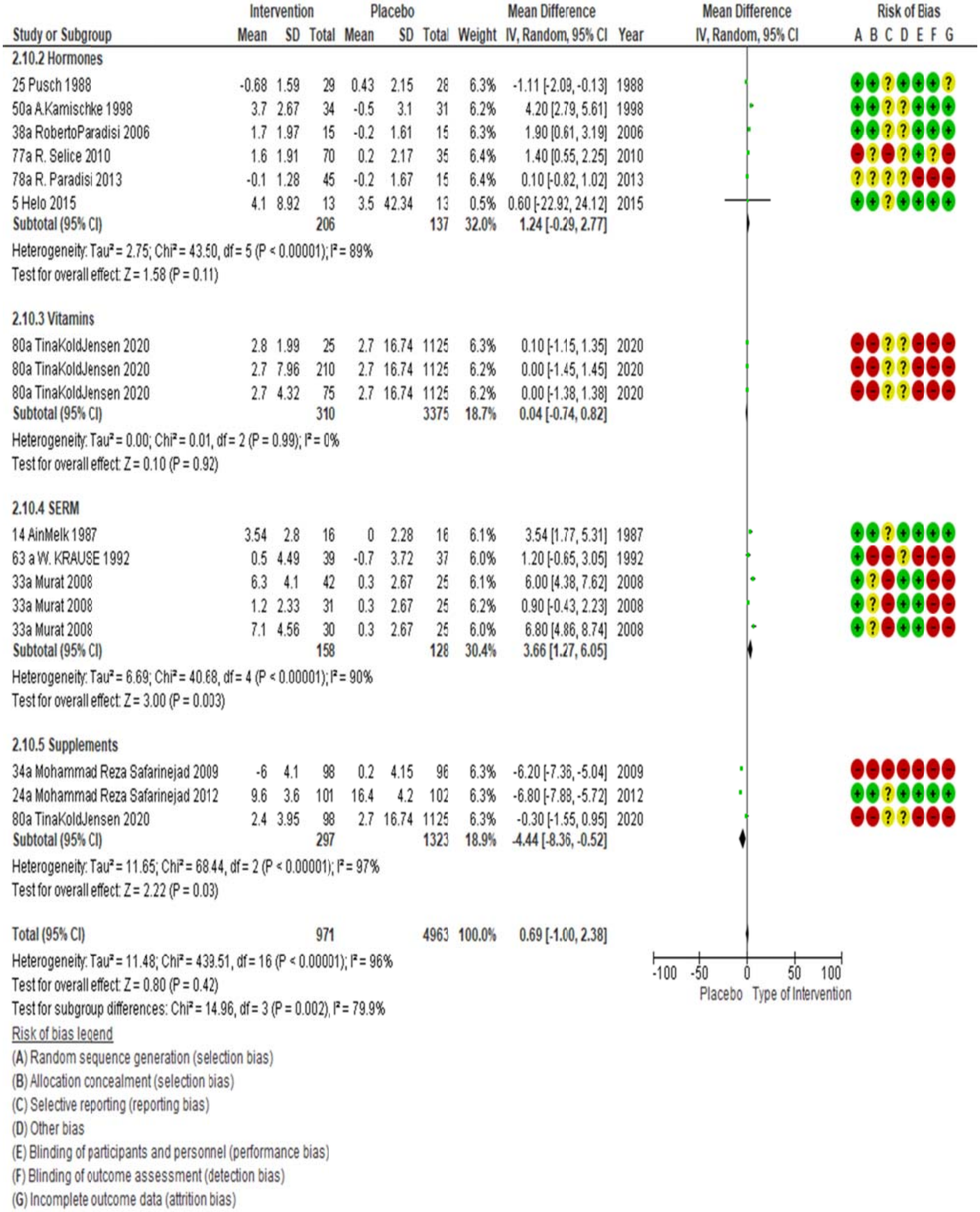

Risk of bias legend

(A) Random sequence generation (selection bias)

(B) Allocation concealment (selection bias)

(C) Selective reporting (reporting bias)

(D) Other bias

(E) Blinding of participants and personnel (performance bias)

(F) Blinding of outcome assessment (detection bias)

(G) Incomplete outcome data (attrition bias)

Supplementary Figure 19: Sub-group meta-analysis (SERM type based) for total serum FSH (n = 3).

Study or Subgroup	Intervention Mean	Intervention SD	Intervention Total	Placebo Mean	Placebo SD	Placebo Total	Weight	Mean Difference IV, Random, 95% CI
**2.12.1 Tamoxifen**								
14 AinMelk 1987	3.54	2.8	16	0	2.28	16	19.9%	3.54 [1.77, 5.31]
33a Murat 2008	7.1	4.56	30	0.3	2.67	25	19.4%	6.80 [4.86, 8.74]
33a Murat 2008	6.3	4.1	42	0.3	2.67	25	20.2%	6.00 [4.38, 7.62]
33a Murat 2008	1.2	2.33	31	0.3	2.67	25	20.8%	0.90 [-0.43, 2.23]
63 a W. KRAUSE 1992	0.5	4.49	39	-0.7	3.72	37	19.7%	1.20 [-0.65, 3.05]
**Subtotal (95% CI)**			**158**			**128**	**100.0%**	**3.66 [1.27, 6.05]**
Heterogeneity: Tau² = 6.69; Chi² = 40.68, df = 4 (P < 0.00001); I² = 90%								
Test for overall effect: Z = 3.00 (P = 0.003)								
**Total (95% CI)**			**158**			**128**	**100.0%**	**3.66 [1.27, 6.05]**

Heterogeneity: Tau² = 6.69; Chi² = 40.68, df = 4 (P < 0.00001); I² = 90%
Test for overall effect: Z = 3.00 (P = 0.003)
Test for subgroup differences: Not applicable

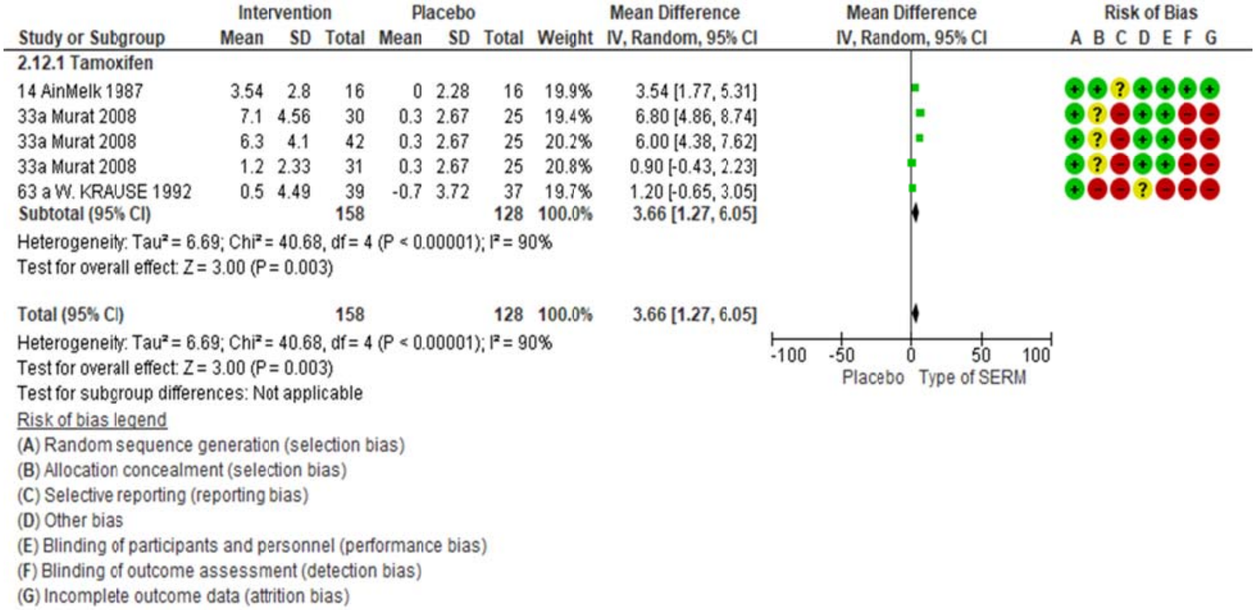

Risk of bias legend
(A) Random sequence generation (selection bias)
(B) Allocation concealment (selection bias)
(C) Selective reporting (reporting bias)
(D) Other bias
(E) Blinding of participants and personnel (performance bias)
(F) Blinding of outcome assessment (detection bias)
(G) Incomplete outcome data (attrition bias)

Supplementary Figure 20: Sub-group meta-analysis (Hormone type based) for total serum FSH (n = 6)

Study or Subgroup	Intervention Mean	Intervention SD	Intervention Total	Placebo Mean	Placebo SD	Placebo Total	Weight	Mean Difference IV, Random, 95% CI
**2.14.1 FSH Dose < 200 IU**								
50a A.Kamischke 1998	3.7	2.67	34	-0.5	3.1	31	18.7%	4.20 [2.79, 5.61]
77a R. Selice 2010	1.6	1.91	70	0.2	2.17	35	20.8%	1.40 [0.55, 2.25]
**Subtotal (95% CI)**			**104**			**66**	**39.5%**	**2.74 [-0.00, 5.48]**
Heterogeneity: Tau² = 3.57; Chi² = 11.10, df = 1 (P = 0.0009); I² = 91%								
Test for overall effect: Z = 1.96 (P = 0.05)								
**2.14.2 FSH Dose >/= 200 IU**								
38a RobertoParadisi 2006	1.7	1.97	15	-0.2	1.61	15	19.2%	1.90 [0.61, 3.19]
78a R. Paradisi 2013	-0.1	1.28	45	-0.2	1.67	15	20.6%	0.10 [-0.82, 1.02]
**Subtotal (95% CI)**			**60**			**30**	**39.8%**	**0.94 [-0.82, 2.70]**
Heterogeneity: Tau² = 1.29; Chi² = 4.96, df = 1 (P = 0.03); I² = 80%								
Test for overall effect: Z = 1.05 (P = 0.29)								
**2.14.3 Testosterone**								
25 Pusch 1988	-0.68	1.59	29	0.43	2.15	28	20.3%	-1.11 [-2.09, -0.13]
**Subtotal (95% CI)**			**29**			**28**	**20.3%**	**-1.11 [-2.09, -0.13]**
Heterogeneity: Not applicable								
Test for overall effect: Z = 2.21 (P = 0.03)								
**2.14.4 Anastrazole**								
5 Helo 2015	4.1	8.92	13	3.5	42.34	13	0.4%	0.60 [-22.92, 24.12]
**Subtotal (95% CI)**			**13**			**13**	**0.4%**	**0.60 [-22.92, 24.12]**
Heterogeneity: Not applicable								
Test for overall effect: Z = 0.05 (P = 0.96)								
**Total (95% CI)**			**206**			**137**	**100.0%**	**1.24 [-0.29, 2.77]**

Heterogeneity: Tau² = 2.75; Chi² = 43.50, df = 5 (P < 0.00001); I² = 89%
Test for overall effect: Z = 1.58 (P = 0.11)
Test for subgroup differences: Chi² = 9.24, df = 3 (P = 0.03), I² = 67.5%

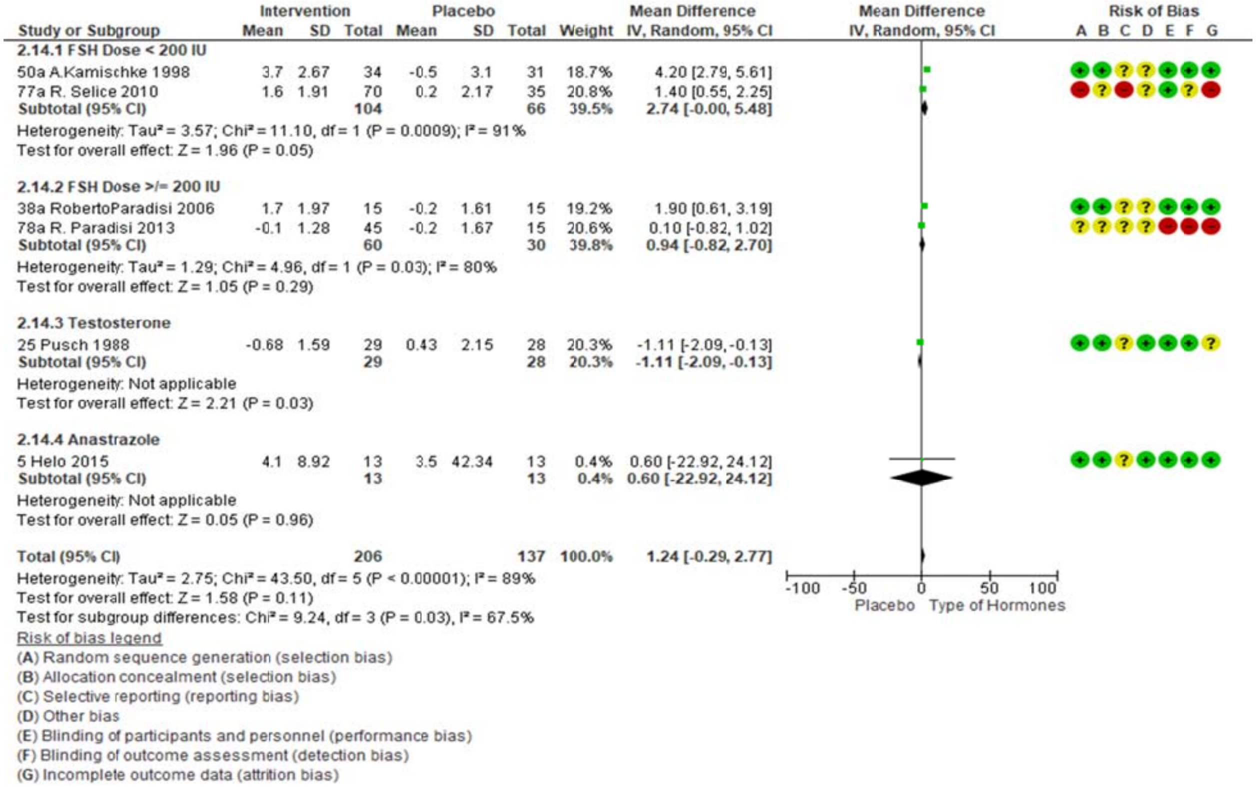

Risk of bias legend
(A) Random sequence generation (selection bias)
(B) Allocation concealment (selection bias)
(C) Selective reporting (reporting bias)
(D) Other bias
(E) Blinding of participants and personnel (performance bias)
(F) Blinding of outcome assessment (detection bias)
(G) Incomplete outcome data (attrition bias)

PRISMA 2009 Checklist

Section/topic	#	Checklist item	Reported on page #
**TITLE**			01
Title	1	Identify the report as a systematic review, meta-analysis, or both.	01
**ABSTRACT**			03-04
Structured summary	2	Provide a structured summary including, as applicable: background; objectives; data sources; study eligibility criteria, participants, and interventions; study appraisal and synthesis methods; results; limitations; conclusions and implications of key findings; systematic review registration number.	03-04
**INTRODUCTION**			03-06
Rationale	3	Describe the rationale for the review in the context of what is already known.	03-05
Objectives	4	Provide an explicit statement of questions being addressed with reference to participants, interventions, comparisons, outcomes, and study design (PICOS).	06
**METHODS**			06-08
Protocol and registration	5	Indicate if a review protocol exists, if and where it can be accessed (e.g., Web address), and, if available, provide registration information including registration number.	08
Eligibility criteria	6	Specify study characteristics (e.g., PICOS, length of follow-up) and report characteristics (e.g., years considered, language, publication status) used as criteria for eligibility, giving rationale.	07
Information sources	7	Describe all information sources (e.g., databases with dates of coverage, contact with study authors to identify additional studies) in the search and date last searched.	06
Search	8	Present full electronic search strategy for at least one database, including any limits used, such that it could be repeated.	06
Study selection	9	State the process for selecting studies (i.e., screening, eligibility, included in systematic review, and, if applicable, included in the meta-analysis).	07
Data collection process	10	Describe method of data extraction from reports (e.g., piloted forms, independently, in duplicate) and any processes for obtaining and confirming data from investigators.	07
Data items	11	List and define all variables for which data were sought (e.g., PICOS, funding sources) and any assumptions and simplifications made.	07
Risk of bias in individual studies	12	Describe methods used for assessing risk of bias of individual studies (including specification of whether this was done at the study or outcome level), and how this information is to be used in any data synthesis.	07
Summary measures	13	State the principal summary measures (e.g., risk ratio, difference in means).	07
Synthesis of results	14	Describe the methods of handling data and combining results of studies, if done, including measures of consistency (e.g., I^2) for each meta-analysis.	07

PRISMA 2009 Checklist

Section/topic	#	Checklist item	Reported on page #
Risk of bias across studies	15	Specify any assessment of risk of bias that may affect the cumulative evidence (e.g., publication bias, selective reporting within studies).	07
Additional analyses	16	Describe methods of additional analyses (e.g., sensitivity or subgroup analyses, meta-regression), if done, indicating which were pre-specified.	07
**RESULTS**			08-15
Study selection	17	Give numbers of studies screened, assessed for eligibility, and included in the review, with reasons for exclusions at each stage, ideally with a flow diagram.	08
Study characteristics	18	For each study, present characteristics for which data were extracted (e.g., study size, PICOS, follow-up period) and provide the citations.	08
Risk of bias within studies	19	Present data on risk of bias of each study and, if available, any outcome level assessment (see item 12).	09
Results of individual studies	20	For all outcomes considered (benefits or harms), present, for each study: (a) simple summary data for each intervention group (b) effect estimates and confidence intervals, ideally with a forest plot.	09
Synthesis of results	21	Present results of each meta-analysis done, including confidence intervals and measures of consistency.	09-13
Risk of bias across studies	22	Present results of any assessment of risk of bias across studies (see Item 15).	09
Additional analysis	23	Give results of additional analyses, if done (e.g., sensitivity or subgroup analyses, meta-regression [see Item 16]).	09-13
**DISCUSSION**			15-18
Summary of evidence	24	Summarize the main findings including the strength of evidence for each main outcome; consider their relevance to key groups (e.g., healthcare providers, users, and policy makers).	15-17
Limitations	25	Discuss limitations at study and outcome level (e.g., risk of bias), and at review-level (e.g., incomplete retrieval of identified research, reporting bias).	17
Conclusions	26	Provide a general interpretation of the results in the context of other evidence, and implications for future research.	17-18
**FUNDING**			18
Funding	27	Describe sources of funding for the systematic review and other support (e.g., supply of data); role of funders for the systematic review.	18

From: Moher D, Liberati A, Tetzlaff J, Altman DG, The PRISMA Group (2009). Preferred Reporting Items for Systematic Reviews and Meta-Analyses: The PRISMA Statement. PLoS Med 6(7): e1000097. doi:10.1371/journal.pmed1000097

For more information, visit: **www.prisma-statement.org**.
